# PFOA Exposure Elicits Quantitative Lipidomic Changes in the Pancreas in a Mouse Model of Pancreatic Cancer

**DOI:** 10.1002/tox.70109

**Published:** 2026-04-24

**Authors:** Barbara A. Hocevar, Lisa M. Kamendulis

**Affiliations:** ^1^ Department of Environmental and Occupational Health School of Public Health, Indiana University Bloomington Indiana USA

**Keywords:** acylcarnitine, ceramide, lipidomics, pancreas, PFOA

## Abstract

Pancreatic cancer is the fourth leading cause of cancer deaths in the US and predicted to rise to second by 2030. Several risk factors have been identified as potential contributors to pancreatic cancer development including lifestyle factors and long‐term exposure to occupational and environmental carcinogens. In mice, exposure to perfluorooctanoic acid (PFOA) leads to diabetic outcomes and pancreatic cancer, while epidemiologic studies have linked PFOA exposure to type 1, type 2, and gestational diabetes, obesity, and pancreatic cancer. While multiple studies have evaluated changes in serum lipid levels in association with PFOA exposure, none to date have evaluated PFOA‐associated lipid alterations in the pancreas. Using the LSL‐KRas^G12D^; Pdx‐1‐Cre (KC) mouse model of pancreatic cancer, we report upregulation of lipid species within the acylcarnitine (CAR) and ceramide (Cer) lipid groups and downregulation of lipids in the phosphatidylcholine, phosphatidylethanolamine (PE), and phosphatidylserine groups following exposure to 1 and 5 ppm PFOA for 6 months. The most upregulated lipids following PFOA exposure included individual Cer and CAR species while PE species comprised the top group of downregulated lipids. Thromboxane B2 (TXB2), a proinflammatory eicosanoid, was the most upregulated lipid in the 1 and 5 ppm KC treatment groups with a fold‐change of ~12‐fold and ~31‐fold in the 1 and 5 ppm KC treatment groups, respectively. As these lipid alterations have been linked to mitochondrial dysfunction, obesity, and inflammation, our results suggest that PFOA‐induced lipid alterations may be involved in adverse health outcomes including diabetes and pancreatic cancer.

## 1 Introduction

Globally, cancer is the second leading cause of death with rates of prevalence, incidence and mortality on the rise. Causes for this increase include lifestyle factors and long‐term exposure to occupational and environmental carcinogens [1]. While pancreatic cancer comprises only 3% of all cancer cases, it is currently the fourth leading cause of cancer death in the United States. Little progress has been made in diagnosing or treating pancreatic cancer, with a median survival time following diagnosis < 6 months, and a 5‐year survival rate of < 9%; thus, a prevention strategy to intervene in pancreatic cancer development and progression would have a large impact in decreasing mortality from this lethal cancer type. Several risk factors have been identified as potential contributors to pancreatic cancer development and progression, including environmental and lifestyle factors, such as smoking, alcohol and consumption of a high fat diet (HFD), and medical conditions such as diabetes, obesity, chronic pancreatitis, and hypertriglyceridemia [2, 3]. Large epidemiologic studies support an association between obesity and increased risk for development of pancreatic ductal carcinoma (PDAC), the most prevalent form of pancreatic cancer [4, 5]. Further, multivariate analysis showed that obesity and high cholesterol were positive predictors of PDAC [6].

Exposure to environmental chemicals has also been linked to the development of obesity, diabetes and pancreatic cancer. Perfluoroalkyl substances (PFAS), a group of ~15 000 environmentally persistent chemicals, exhibit widespread ground and surface water contamination due to their extensive use [7, 8]. Perfluorooctanoic acid (PFOA) and its replacement compound GenX both induce pancreatic acinar cell tumors in rodents [9, 10] and we have previously demonstrated that exposure to PFOA causes a ~2‐fold increase in pancreatic lesion area in the LSL‐KRas^G12D^; Pdx‐1‐Cre (KC) mouse model of pancreatic cancer [11]. In epidemiologic studies, exposure to PFOA, as well as other members of the PFAS group, has been associated with an increased risk for pancreatic cancer [12, 13, 14, 15]. In addition, in vitro and in vivo studies using pancreatic cancer cell lines have shown that exposure to perfluorooctane sulfonate and 6:2 chlorinated polyfluorinated ether sulfonate triggered resistance to chemotherapeutic agents used in the treatment of pancreatic cancer [16, 17].

Multiple studies in humans have shown that PFOA can affect pancreatic function and elicit an obesogenic phenotype. A strong association between PFOA exposure and hyperlipidemia, a cause of both acute and chronic pancreatitis, was observed in occupationally exposed and the general population [18, 19, 20]. In addition, plasma PFOA levels were correlated with risk of type 2 diabetes (T2D) in the Nurse's Health Study II [21] and increased diabetes mortality in occupationally exposed workers [22]. In utero exposure to PFOA resulted in obesity in female offspring, concomitant with elevated serum leptin and insulin levels [23, 24]. Obesity, a known driver of T2D, is characterized by quantitative changes in serum lipid profiles [25, 26, 27]. Increased intrapancreatic fat deposition has been shown to increase the risk for PDAC [28] and elevation of lipid levels is seen in the serum and tissue of pancreatic cancer patients as compared to benign pancreatic hyperplasia and healthy controls [29]. While several studies have evaluated changes in serum lipid levels in association with PFOA exposure, none to date have linked PFOA exposure with lipidomic analysis of pancreatic tissue. Here, we sought to determine if PFOA exposure induces quantitative lipidomic alterations in the pancreas that may play a role in pancreatic lesion promotion that we observe in PFOA‐treated KC mice.

## 2 Methods

### 2.1 Mouse Model and Treatment Design

LSL‐KRas^G12D^ and Pdx‐1 Cre mice (Jackson Laboratory) were bred and maintained as heterozygous lines, and genotyping was performed by PCR analysis as previously described [11]. Beginning at 8 weeks of age, male and female LSL‐KRas^G12D^;Pdx‐1 Cre (KC) mice either received tap water (control) or water containing 1 or 5 ppm PFOA (Sigma 171468, 96% purity). Mice were euthanized by CO_2_ asphyxiation at 6 months of age, at which time tissues were collected and processed as described [11]. The PFOA dose chosen was based on the US EPA 2016 lifetime health advisory level (HAL) guideline concentration for PFOA in drinking water set at 70 ppt, and was derived from a developmental toxicity study in mice in which a dose of 1 mg/kg BW/d PFOA represented the lowest effect dose [30]. To determine equivalence of dosing in water to that of oral gavage, we exposed C57Bl/6 mice to 0, 5, 10, and 20 ppm PFOA in drinking water for 7 days, quantified PFOA levels in serum, and compared the levels to those obtained in our 7 day gavage study [31]. We observed a linear increase of PFOA in serum by both routes of administration (Table S1). Using these data, the gavage dose of 1 mg/kg BW/d used to set the HAL for PFOA is equivalent to approximately 3.1 ppm PFOA in drinking water, which lies between the 1 and 5 ppm concentrations we used.

### 2.2 Ethics Statement

Mice were maintained in accordance with the NIH Guide for the Care and Use of Laboratory Animals and experiments were approved by the Indiana University Bloomington Institutional Animal Care and Use Committee.

### 2.3 Histology Analysis

Alcian Blue (AB) staining was used to quantify PanIN lesions on paraffin sections according to manufacturer's instructions (AB [pH 2.5] staining kit, Vector Labs, Burlingame, CA). Dehydrated stained sections were mounted with Histomount (Thermo Fisher Scientific) and scanned using a whole‐slide imaging system at 20× magnification (Motic EasyScan, British Columbia). AB lesion areas of digitized images were quantified using Aperio ImageScope software (Leica Biosystems, Buffalo Grove, IL) and expressed as the % area occupied by AB‐positive lesions relative to the total area of the pancreatic section. To quantify collagen deposition, Masson's trichrome (MT) staining (Sigma‐Aldrich) was performed following the manufacturer's instructions. Stained sections were dehydrated then mounted with Histomount and digitized using a Motic EasyScan whole slide imaging system at 20× magnification (Motic, British Columbia). TIFF images were analyzed using ImageJ software (NIH, Bethesda, MD) to quantify the relative % of collagen content in each section.

### 2.4 Lipidomic Analysis

#### 2.4.1 Chemicals and Reagents

HPLC‐grade acetonitrile (ACN), methanol (MeOH), isopropanol (IPA), dichloromethane (CH_2_Cl_2_), and tert‐butyl methyl ether (MTBE) were purchased from Merck (Darmstadt, Germany). HPLC‐grade formic acid (FA) and ammonium formate (AmFA) were purchased from Sigma‐Aldrich (St. Louis, MO, USA). Ultrapure water was obtained by Milli‐Q system (Millipore, Billerica, MA). Lipid standards were purchased from Sigma‐Aldrich or Avanti Polar Lipids (Alabaster, AL).

#### 2.4.2 Sample Preparation and Extraction

All lipidomic sample preparation and analysis was performed by Metware Biotechnology Inc. (Woburn, MA). The samples were thawed on ice. Approximately 10 mg of each sample was weighed and homogenized by ball‐mill in 1 mL of extraction solution (MTBE:Methanol, 3:1, v/v) with internal standards, then vortexed for 15 min. Next, 200 μL of water was added to the mixture, then vortexed for 1 min and incubated at 4°C for 10 min. After centrifugation at 12000 rpm for 10 min (4°C), 200 μL of the upper phase was collected then dried at 20°C. The residue was reconstituted using 200 μL of acetonitrile‐isopropanol (1:1, v/v), then vortexed for 3 min and centrifuged at 12000 rpm for 3 min. A 120 μL of the supernatant was used for LC–MS analysis.

#### 2.4.3 HPLC Conditions

The sample extracts were analyzed using an LC‐ESI‐MS/MS system (UPLC, Nexera LC‐40, Shimadzu Scientific Instruments, Columbia, MD; MS, Triple Quad 6500+, SCIEX, Framingham, MA). The analytical conditions were as follows: UPLC: column, Thermo AccucoreC30 (2.6 μm, 2.1 mm × 100 mm i.d.); solvent system, (A) acetonitrile/water (60/40, v/v, 0.1% formic acid, 10 mmol/L ammonium formate), (B) acetonitrile/isopropanol (10/90 v/v, 0.1% formic acid, 10 mmol/L ammonium formate); gradient program, A/B (80:20, v/v) at 0 min, 70:30 v/v at 2.0 min, 40:60 v/v at 4 min, 15:85 v/v at 9 min, 10:90 v/v at 14 min, 5:95 v/v at 15.5 min, 5:95 v/v at 17.3 min, 80:20 v/v at 17.3 min, 80:20 v/v at 20 min; flow rate, 0.35 mL/min; temperature, 45°C; injection volume: 2 μL.

#### 2.4.4 ESI‐MS/MS Conditions

Linear ion trap (LIT) and triple quadrupole (QQQ) scans were acquired on a triple quadrupole‐LIT mass spectrometer (QTRAP), QTRAP 6500+ LC‐MS/MS System, equipped with an ESI Turbo Ion‐Spray interface, operating in positive and negative ion mode and controlled by Analyst 1.6.3 software (Sciex). The ESI source operation parameters were as follows: ion source, turbo spray; source temperature 500°C; ion spray voltage (IS) 5500 V (Positive), −4500 V (Negative); Ion source gas 1 (GS1), gas 2 (GS2), curtain gas (CUR) was set at 45, 55, and 35 psi, respectively. Instrument tuning and mass calibration were performed with 10 and 100 μmol/L polypropylene glycol solutions in QQQ and LIT modes, respectively. QQQ scans were acquired as MRM experiments with collision gas (nitrogen) set to 5 psi. Multiple optimizations for declustering potential (DP) and collision energy (CE) were performed for individual MRM transitions. A specific set of MRM transitions were monitored for each period according to the metabolites eluted within this period. Lipids were annotated based on their retention time and ion‐pair information from MRM transitions using a proprietary database developed by Metware Biotechnology.

#### 2.4.5 Data Collection and Quality Control

Following data acquisition, raw peak intensity data (*.wiff) were extracted from Analyst software (SCIEX, Framingham, MA, USA) using MultiQuant 3.0.3 software (SCIEX, Framingham, MA, USA). Parameters for extracting peaks from the Multiple Reaction Monitoring (MRM) chromatograms were set as follows: Gaussian Smooth Width (0 points), RT Half Windows (30 s), Min.Peak Width (two points), Min.Peak Height (0), Baseline Noise Percentages (70%), Baseline Sub. Window (1.0 min), Peak Splitting (two points), the minimum intensity of chromatographic peak filtration was 1000 cps, the minimum signal‐to‐noise ratio was 5, and the retention time deviation was < 0.2 min. Based on the peak area of the QC samples, the substances with the detection rate of less than 50% and the CV of more than 0.3 were deleted, and the final list of substances was obtained. Internal standard method was used for quantification, with each lipid subclass quantified using 1–2 lipid internal standards. The composition of the internal standards is provided in Table S2. All internal standards were stable response internal standards. A quality control (QC) sample was prepared from a mixture of all sample extracts to examine the reproducibility of the lipidomic process. During data collection, a QC sample was inserted for at least every 10 test samples. The QC data are presented in Figure S1.

#### 2.4.6 Lipidomics Analytical Methods

Mass spectrometric data were processed using Analyst 1.6.3 software (AB Sciex). Unsupervised principal component analysis (PCA) was performed using the statistical function prcomp within R (www.r‐project.org). The data were unit variance scaled prior to performing unsupervised PCA. To identify differentially expressed lipids between groups, orthogonal partial least squares discriminant analysis (OPLS‐DA) was performed using MetaboAnalyst 4.0 software. Log transformation (log2) and mean centering of the data were performed prior to OPLS‐DA. To avoid overfitting, a permutation test (200 permutations) was performed. The OPLS‐DA model validation is shown in Figure S2. Variable importance in projection (VIP) values were extracted from OPLS‐DA modeling. For two‐group analysis, lipids were defined as differentially expressed if the VIP > 1 and *p* value < 0.05. *p* value was calculated using a *t*‐test if the data distribution passes the normality test (Shapiro–Wilk test) and Wilcoxon rank sum test if the data distribution fails the normality test. The fold change was calculated as the means of the test group over the means of the control group, for example, for 1 ppm KC versus CTL KC, 1 ppm KC is the test group and the CTL KC is the control group. A two‐fold change indicates a two‐fold change in 1 ppm KC in relation to CTL KC.

## 3 Results

### 3.1 PanIN Lesion Area in the KC Mouse Model Is Increased by PFOA Exposure

Mutation of the *KRAS* gene is found in 90% of human pancreatic ductal adenocarcinoma (PDAC), the most common form of pancreatic cancer [32, 33]. Targeting constitutively active KRas (KRas^G12D^) to early pancreatic progenitors using the Pdx‐1 or Ptf1a gene promoters in mice results in a histologic progression of pancreatic cancer that closely mimics the changes seen in human PDAC; thus, the LSL‐KRas^G12D^;Pdx‐1 Cre (KC) mouse model is widely used as a representative model for pancreatic cancer development [34]. Using the KC mouse model, we have previously shown that exposure to 5 ppm PFOA in drinking water resulted in a ~2‐fold increase in lesion area and lesion number [11]. As global efforts to reduce PFOA manufacture have resulted in lower exposure levels, in this study, we evaluated a lower dose of PFOA (1 ppm) for its effects on lesion growth and severity in the KC mouse model. Male and female KC mice were provided control tap water or tap water containing 1 ppm PFOA, beginning at 8 weeks of age, then sacrificed at 6 months of age for collection of serum and tissues. Pancreata were processed for histopathologic evaluation (H&E stain), AB staining, a marker to identify pancreatic intraepithelial neoplastic (PanIN) lesion area, and MT staining to identify collagen deposition (Figure 1A). PFOA exposure increased PanIN lesion area from 4.91% ± 1.61% in CTL KC mice to 7.28% ± 2.5% in 1 ppm PFOA KC mice; levels comparable to that elicited by 5 ppm PFOA (7.71% ± 3.01%) (Figure 1B). Analysis of collagen deposition in KC mice revealed that PFOA increased the area of the pancreas occupied by collagen in a dose‐dependent manner from 8.84% ± 4.50% in CTL KC to 14.58% ± 6.69% in 1 ppm PFOA KC and 15.76% ± 6.28% in 5 ppm PFOA KC mice (Figure 1C). We also observed that PFOA exposure led to increased intra‐ and peri‐pancreatic fat deposition (Figure 2), a condition which is termed “fatty pancreas” [28].

**FIGURE 1:**
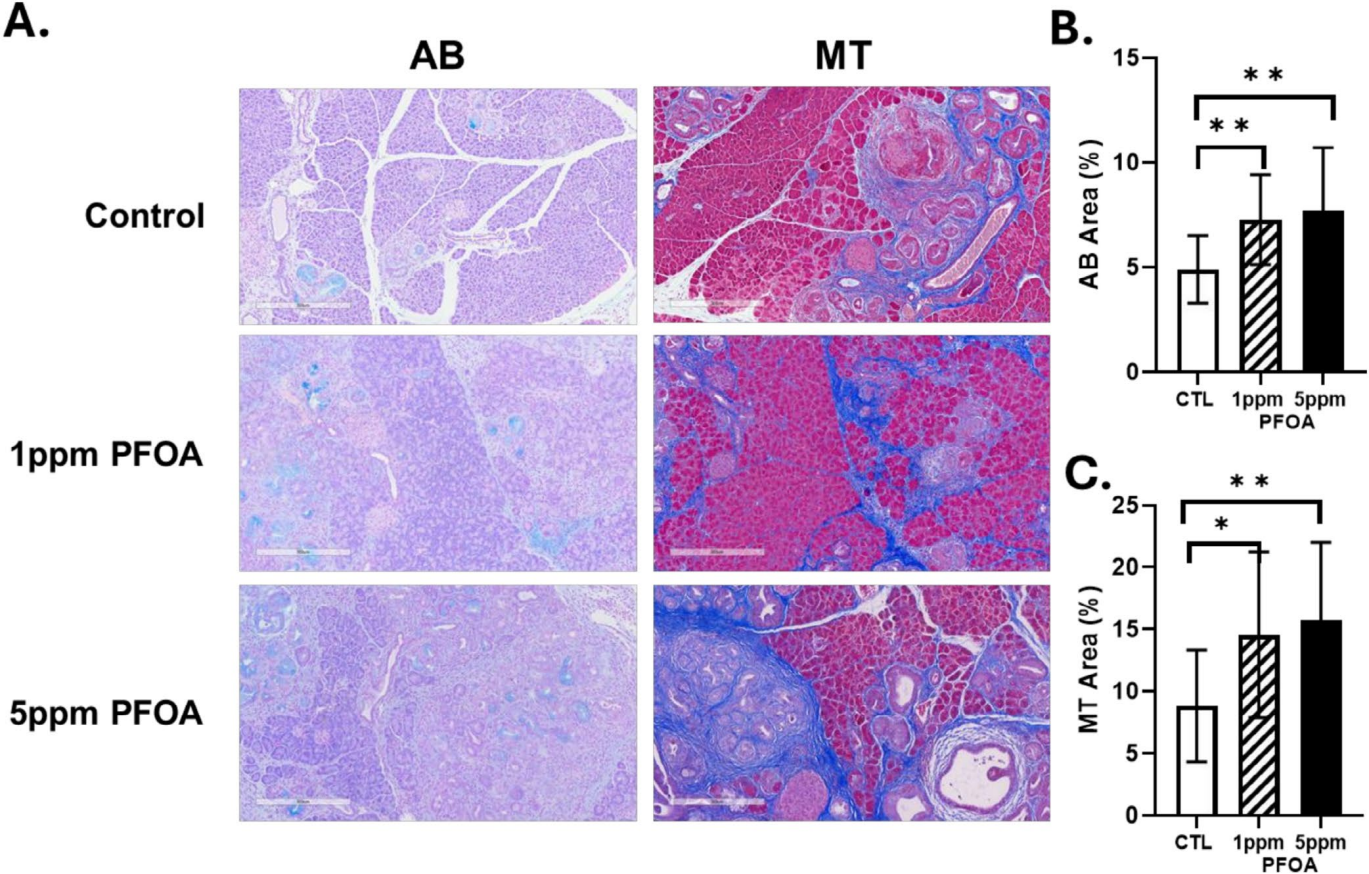
PFOA exposure leads to increased PanIN area and severity. (A) Representative sections of Alcian Blue (AB) and Masson's Trichrome (MT) stained pancreatic sections from 6 month‐old KC mice exposed to tap water, Control (CTL), 1 ppm and 5 ppm PFOA. (B) Lesion area was quantitated from AB‐stained sections and (C) MT‐stained sections and expressed as area of the total pancreas occupied by specific staining. *n* = 9–18 mice/group *Significantly different from CTL KC mice, *p* < 0.05, **p < 0.005.

**FIGURE 2:**
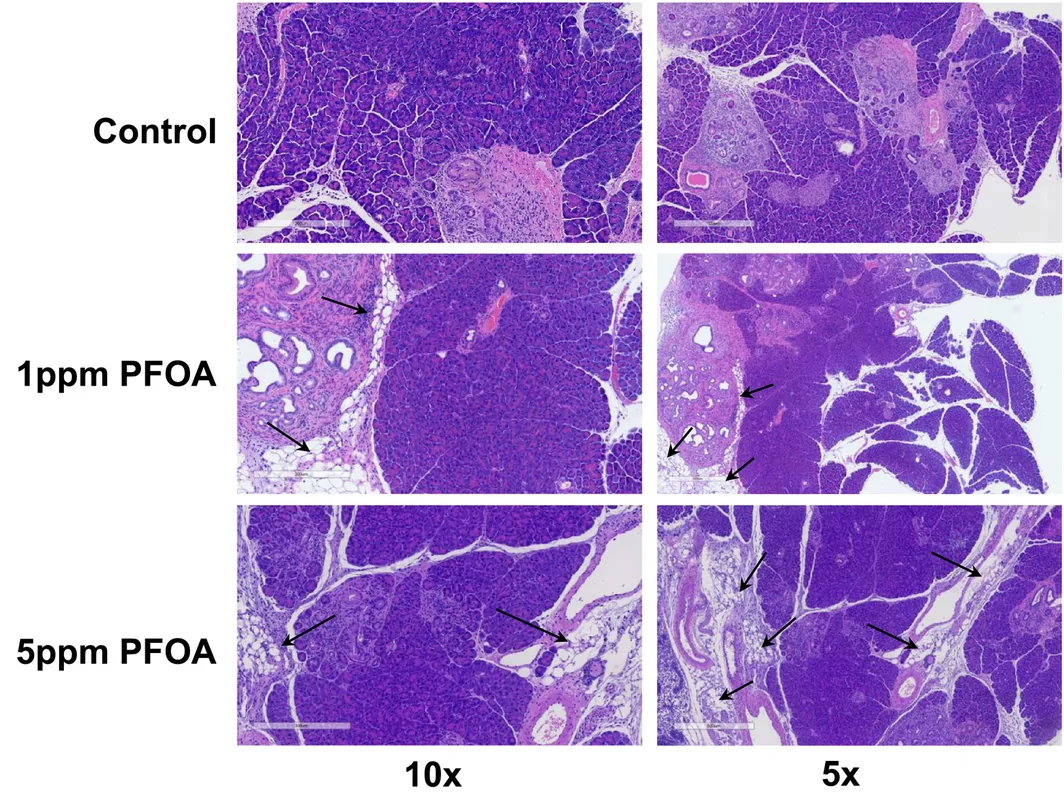
PFOA exposure leads to fat deposition in the pancreas. Representative H&E images at 10× magnification, left image, and 5× magnification, right image. Arrows indicate areas of intra‐ and peri‐pancreatic fat deposition in the pancreas of CTL, 1 and 5 ppm PFOA KC mice. Bar represents 300 μm in 10× images and 500 μm in 5× images.

### 3.2 Alterations in Pancreatic Lipid Classes Following PFOA Exposure

To assess potential linkages between PFOA exposure and lipid metabolism, we performed quantitative lipidomic analysis of pancreata derived from the CTL KC and KC mice exposed to 1 and 5 ppm PFOA. Using ultra‐performance liquid chromatography–tandem mass spectrometry (UPLC‐MS/MS), 1450 lipids comprising 41 lipid classes were detected in pancreatic tissues. While 21 individual lipid classes were significantly different in either 1 or 5 ppm KC mice compared to CTL KC, changes in 17 lipid classes were common to both treatment groups, with five exhibiting increased levels, including acylcarnitine (CAR) and ceramides (Cer‐AP, Cer‐NP, CerP), and 12 with decreased levels including phosphatidylcholine (PC), phosphatidylethanolamine (PE), and phosphatidylserine (PS) (Table 1). Measured values for all lipid classes are shown in Table S3.

**TABLE 1:** Lipid classes significantly different relative to control.

	Number found	Control		1 ppm PFOA		5 ppm PFOA	
		Mean^#^	SD	Mean^#^	SD	Mean^#^	SD
All	1450	78322.9	4768.6	72827.6	12007.6	**70032.6**	**6625.9**
BMP	1	6.5	3.4	**10.6**	**3.0**	5.9	3.0
CAR	58	12.8	2.5	**20.1**	**4.4**	**20.5**	**5.0**
Cer‐AP	33	352.4	40.4	**1477.6**	**1331.2**	**619.0**	**227.7**
Cer‐NP	29	853.7	207.9	**1745.0**	**990.1**	**1386.1**	**441.7**
CerP	5	388.8	65.6	**528.8**	**103.3**	**584.0**	**134.3**
CoQ	3	149.2	26.7	**110.0**	**23.4**	**96.1**	**19.1**
Eicosanoid	19	8.6	6.3	14.1	9.3	**16.7**	**6.2**
LNAPE	15	262.7	35.7	**142.1**	**62.6**	**190.1**	**72.3**
LPA	8	35.5	6.5	**23.5**	**4.2**	**25.1**	**10.5**
LPC‐O	14	6.4	1.6	**13.3**	**4.1**	**12.0**	**5.1**
LPE	29	9477.0	987.0	**7997.8**	**1225.8**	8610.7	2664.7
LPE‐P	5	546.3	115.1	**269.3**	**100.3**	467.4	582.0
LPI	14	213.9	16.2	**138.4**	**59.7**	**150.6**	**30.7**
PC	103	12813.6	1218.1	**8885.8**	**2254.7**	**10470.9**	**1738.9**
PE	115	3472.2	232.9	**2551.5**	**392.8**	**2956.8**	**506.5**
PE‐O	87	947.1	70.2	**685.1**	**118.8**	**746.9**	**136.8**
PE‐P	53	3967.3	931.0	**2425.9**	**668.1**	**2555.4**	**792.8**
PG	95	281.1	16.5	**190.4**	**36.3**	**201.7**	**28.4**
PI	67	1088.6	127.5	**675.4**	**204.0**	**770.9**	**206.8**
PS	110	23988.6	3528.5	**17276.7**	**3967.5**	**19641.3**	**4349.9**
SPH	3	3.1	0.8	**2.1**	**0.3**	**2.3**	**0.5**

*Note:* ^#^Units are nmol/g tissue. Bolded = significantly different from control, *p* < 0.05.

### 3.3 Lipidomic Alterations Induced by 1 and 5 ppm PFOA Exposure

PCA was performed on each pair of sample groups to examine overall differences between each group and variation between samples within a group. As seen in Figures 3 and 2D PCA analysis demonstrates that pancreatic lipid composition in both 1 and 5 ppm PFOA treated KC mice could be distinguished from CTL KC samples (Figure 3A,B). To further refine differential lipid changes between CTL KC and the two treatment groups, partial least squares‐discriminant analysis was combined with orthogonal signal correction (OPLS‐DA) which showed a clear separation between 1 and 5 ppm PFOA treated KC mice versus CTL KC mice (Figure 3C,D). Based on the results of OPLS‐DA, multivariate analysis of VIP was used to identify differentially expressed lipids between the groups. Differentially expressed lipids were further filtered by combining the *p* value/False Discovery Rate (FDR) or fold‐change (FC) values from univariate analysis. Volcano plots were generated for the comparison of 1 ppm KC to CTL KC and 5 ppm KC to CTL KC, using metabolites with a VIP > 1 and *p*‐value < 0.05 as cutoff values designating significantly different expression. As shown in Figure 4A, this analysis identified 533 differentially expressed lipids in the 1 ppm KC versus CTL KC group, with 223 lipids upregulated and 310 lipids downregulated. In comparison, 454 lipids were identified as significantly different in the 5 ppm KC versus CTL KC groups, with 225 lipids upregulated and 229 downregulated (Figure 4B). We next visualized the differential lipids in each subclass using a scatter plot combining both the logarithmic value of the content difference and the VIP value (Figure 5A,B). With this visualization, we observed that most metabolites in some lipid classes, such as CAR and Eicosanoid, were increased in the pancreas in both the 1 and 5 ppm KC mice relative to CTL KC mice, while lipids in other classes, such as PI and lysophosphatidyl inositol (LPI) were largely decreased (Figure 5A,B). In addition, differentially expressed lipid species in some lipid classes, such as PC and PE, were split between an increase or decrease in PFOA treated KC mice versus CTL KC mice. The complete list of significantly differentially expressed lipids between 1 ppm KC and CTL KC and 5 ppm KC and CTL KC is provided in Tables S4 and S5.

**FIGURE 3:**
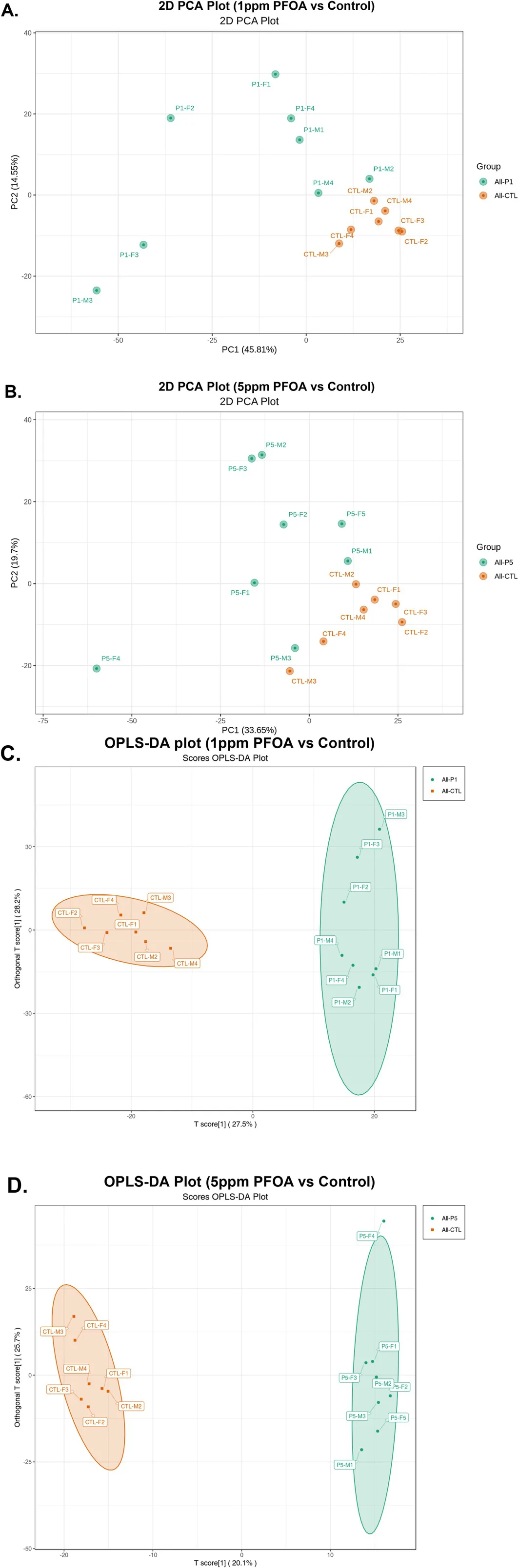
PCA and OPLS‐DA plots. (A and B) 2D PCA plots of 1 ppm PFOA KC mice versus CTL KC mice (A) and 5 ppm PFOA KC mice versus CTL KC mice (B). PC1 represents the first principal component and PC2 represents the second principal component and the percentages on the axis represent the interpretation rate of the principal component to the data set. Each dot represents a sample, with PFOA exposure in green and CTL in orange. (C and D) OPLS‐DA plots of 1 ppm PFOA KC mice versus CTL KC mice (C) and 5 ppm PFOA KC mice versus CTL KC mice (D). The *X*‐axis denotes the predicted principal component and the difference between the groups can be seen in the horizontal direction. The *Y*‐axis represents the orthogonal principal component and the vertical direction shows the difference within the group. Percentage indicates the degree to which the component explains the data set. Each dot represents a sample, with PFOA exposure in green and CTL in orange.

**FIGURE 4:**
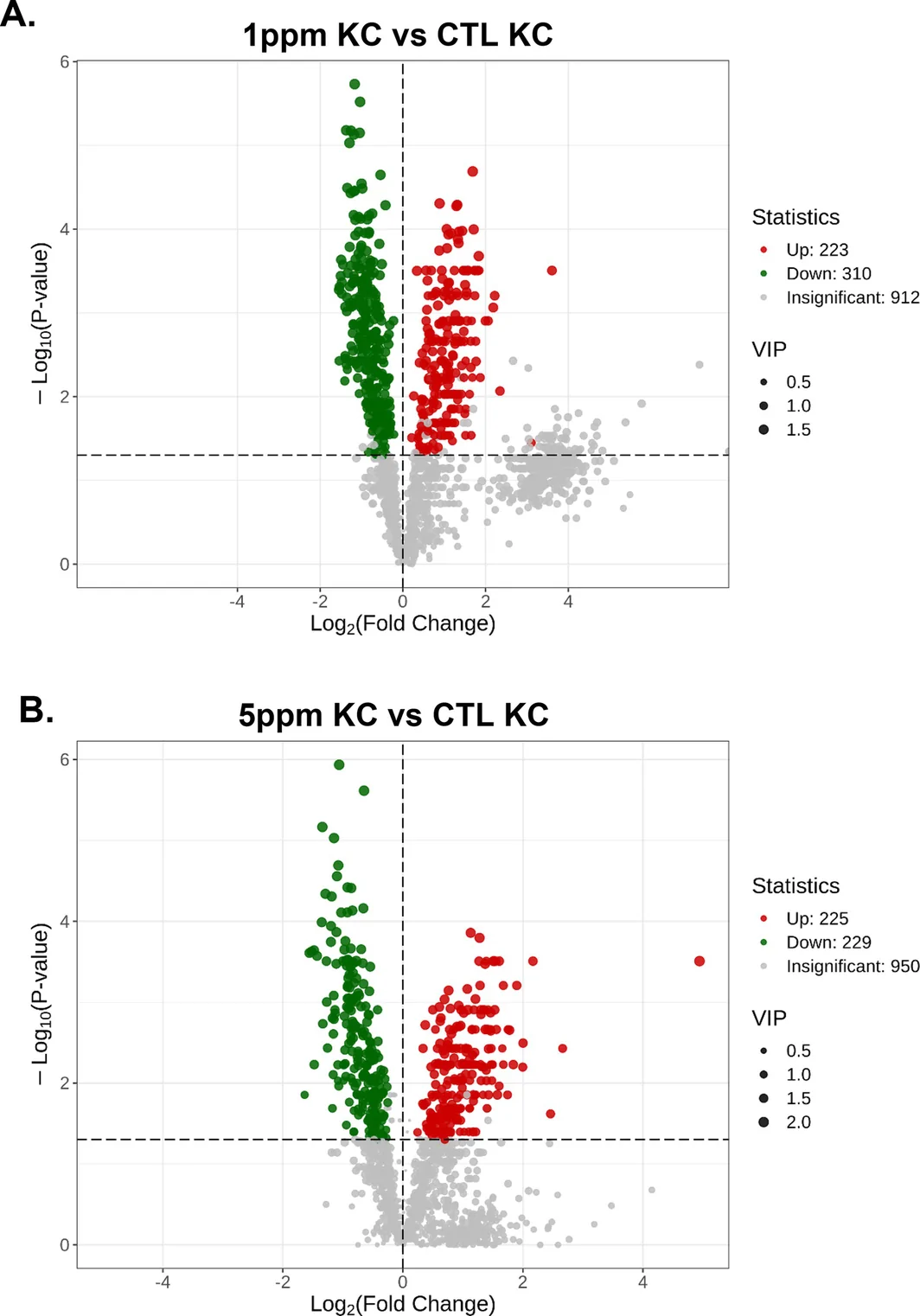
Distribution of differentially expressed lipids following PFOA exposure in KC mice. Volcano plots show the distribution of significantly expressed lipids in the pancreas of KC mice following exposure to 1 ppm (A) and 5 ppm PFOA (B) compared to CTL KC. The horizontal coordinate represents the difference in expression of lipids Log_2_ Fold Change and the vertical coordinate represents the significance level −Log_10_ *p* value. Each point in the diagram represents a lipid; significantly upregulated lipids are indicated in red and significantly downregulated lipids are indicated in green. Size of the dot indicates the VIP value.

**FIGURE 5:**
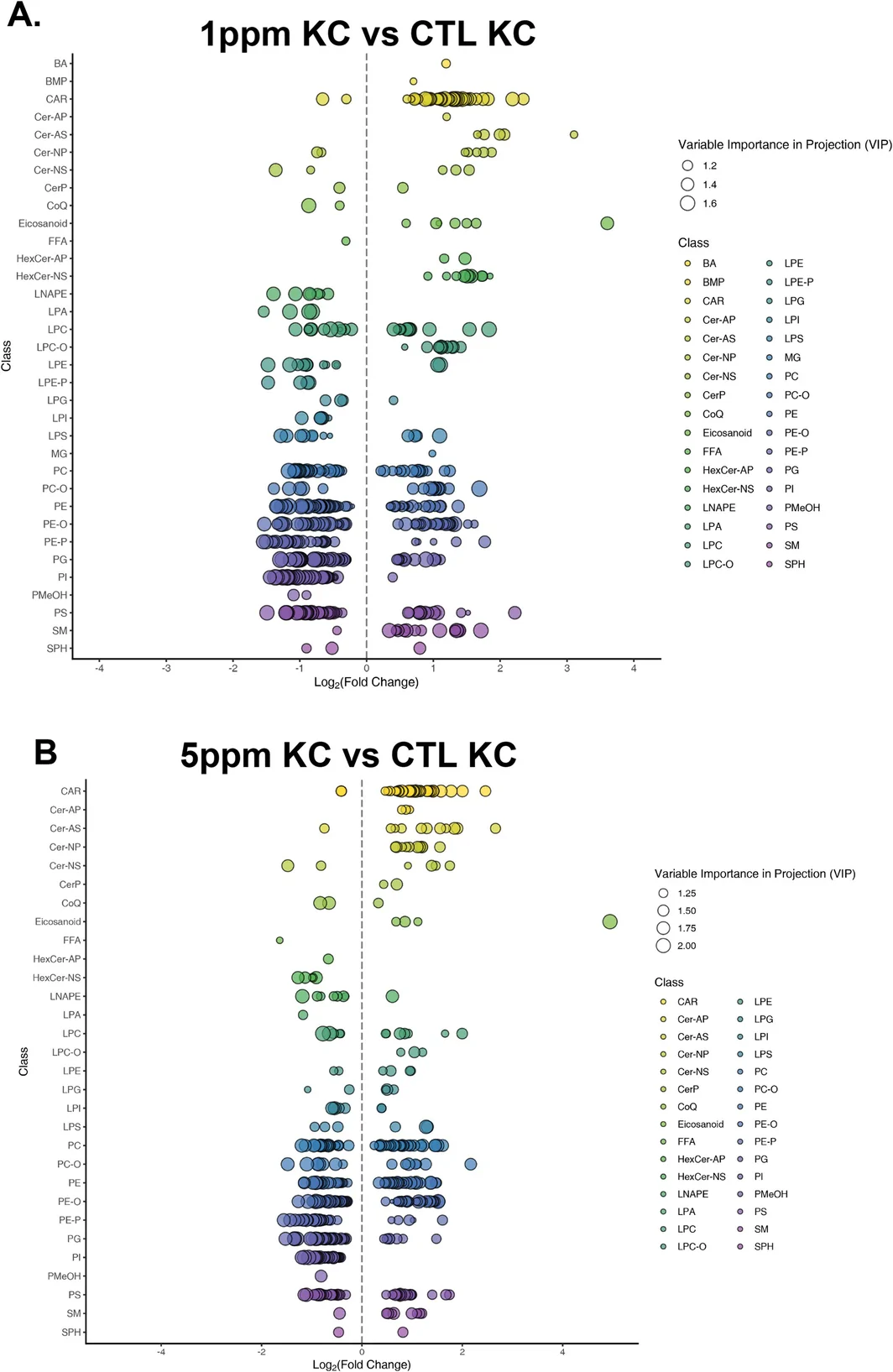
Differential expression of lipid classes and lipid species by PFOA exposure. Scatter plot of significantly expressed lipid classes in the pancreas of KC mice following exposure to 1 ppm (A) and 5 ppm PFOA (B) compared to CTL KC. Each dot in the graph indicates a lipid and different colors indicate different lipid subclasses. The horizontal coordinate indicates the Log_2_ Fold Change and the size of the dots represents the VIP value.

### 3.4 Identification of Individual Lipid Species With Significantly Different Expression in 1 and 5 ppm KC Mice

To compare differential expression of individual lipid species, *Z*‐score standardization was applied to normalize the relative content of the differential lipids. Figure 6 shows the *Z*‐value map of the top 50 differentially expressed lipid species ranked by VIP scores for 1 ppm KC versus CTL KC (Figure 6A) and 5 ppm KC versus CTL KC (Figure 6B). We next determined which differential lipid species were shared or unique between the 1 ppm KC and 5 ppm KC groups versus CTL KC. Figure 7 shows the Venn diagram of differential expression between the groups with 307 lipids in common in both 1 ppm KC and 5 ppm KC versus CTL KC with 226 and 147 lipids unique to the 1 ppm KC and 5 ppm KC groups, respectively (Figure 7). Table 2 lists the top 10 most increased and decreased lipid species in common to both treatment groups while the entire list of differentially expressed lipids common to both groups can be found in Table S6. As can be seen, individual Cer and CAR species were among the most upregulated lipids following PFOA exposure, while PE‐P species comprised the top group of downregulated lipid species (Table 2). The individual lipid species which demonstrated the greatest significant FC in both comparison groups was thromboxane B2 (TXB2), which exhibited dose‐dependent upregulation of ~12‐fold and ~31‐fold versus the CTL KC in the 1 and 5 ppm KC treatment groups, respectively.

**FIGURE 6:**
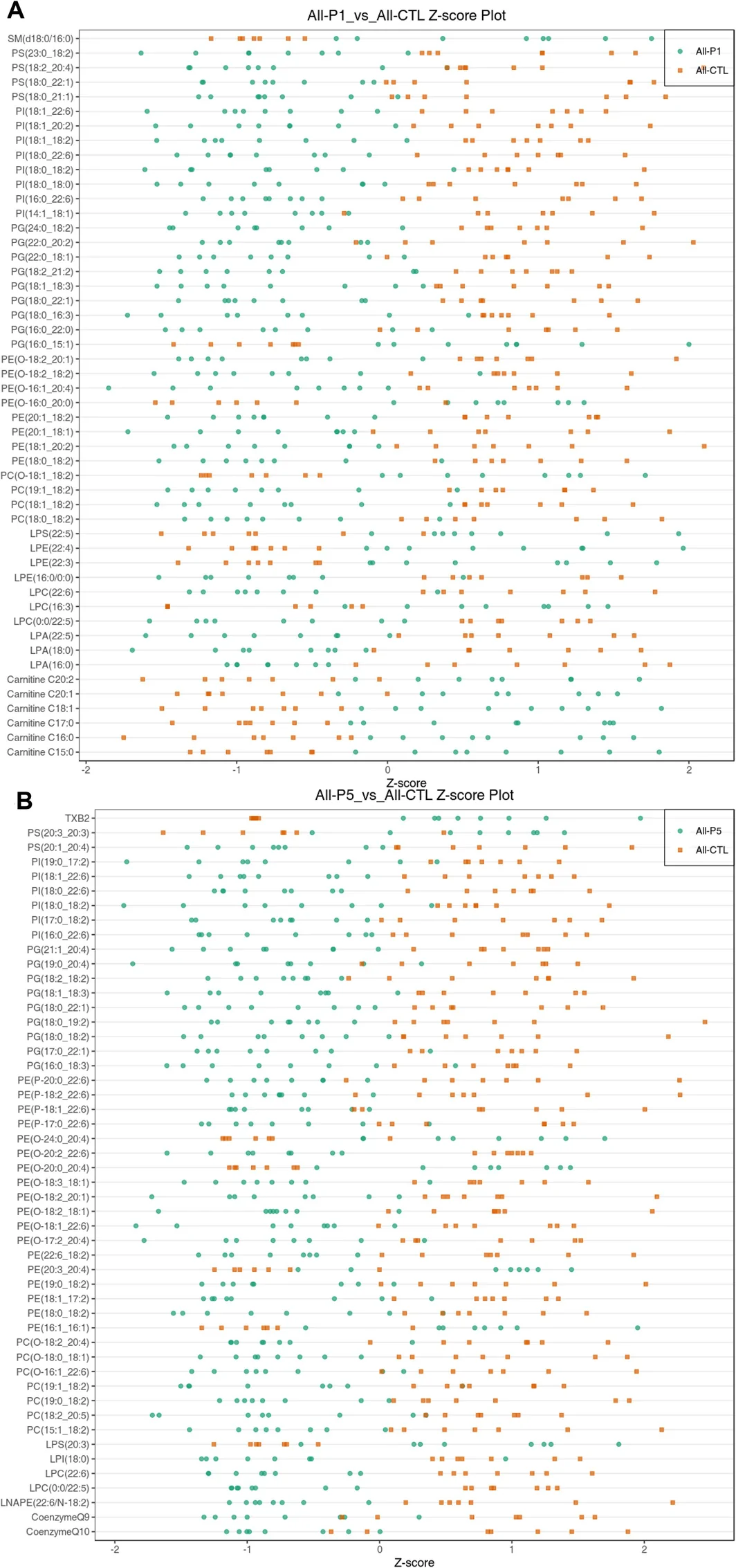
Top 50 differentially expressed lipid species in the pancreas of KC mice exposed to 1 and 5 ppm PFOA. *Z*‐value map of differential lipids in the pancreas of KC mice following exposure to 1 ppm (A) and 5 ppm PFOA (B) versus CTL KC. The *X*‐axis represents the *z*‐score and the *Y*‐axis represents the differential lipids. The colored dots represent individual pancreas samples of the different treatment groups; green circles represent PFOA treatment groups and orange squares represent CTL KC. The top 50 lipids based on VIP score are shown for each treatment group.

**FIGURE 7:**
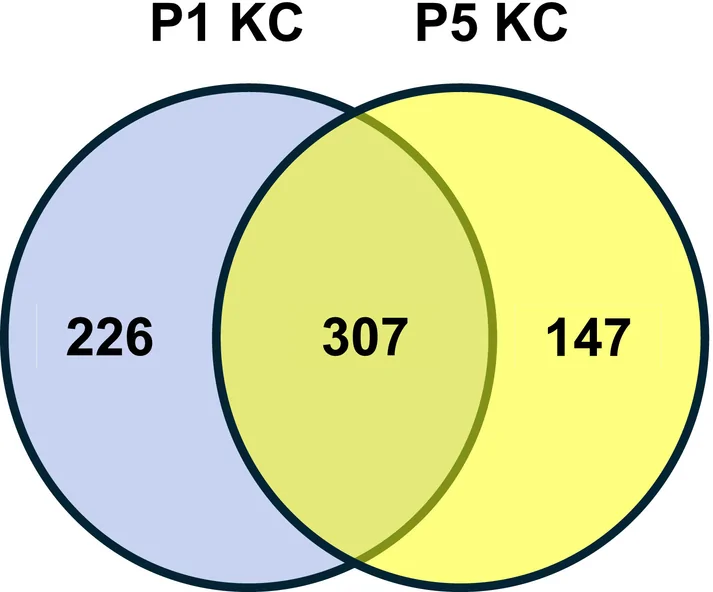
Venn diagram of differential lipid expression. Venn diagram of significantly altered shared and unique lipids in the pancreas of KC mice exposed to 1 and 5 ppm PFOA versus CTL KC. Each circle represents a comparison group; the number in the overlap represents the number of common differential lipids between the two groups, while the number in the non‐overlap area represents the number of unique differential lipids in the comparison groups.

**TABLE 2:** Top 10 lipids significantly increased and decreased in the pancreas by exposure to both 1 and 5 ppm PFOA.

Compounds	Class I	Class II	1 ppm KC v. CTL KC					5 ppm KC v. CTL KC				
			VIP	*p*	FDR	Fold change	Log2FC	VIP	*p*	FDR	Fold change	Log2FC
TXB2	FA	Eicosanoid	1.4229	0.0003	0.0057	12.1561	3.6036	2.037	0.000	0.010	30.767	4.943
Carnitine C22:5	FA	CAR	1.3177	0.0086	0.0342	5.0883	2.3472	1.439	0.024	0.089	5.506	2.461
Carnitine C17:1	FA	CAR	1.4771	0.0009	0.0083	4.5456	2.1845	1.565	0.003	0.030	4.008	2.003
Cer(d20:1/24:1(2OH))	SP	Cer‐AS	1.2846	0.0012	0.0099	4.1744	2.0616	1.542	0.001	0.015	3.727	1.898
Cer(d20:1/24:0(2OH))	SP	Cer‐AS	1.2747	0.0012	0.0099	3.9775	1.9919	1.243	0.006	0.038	3.193	1.675
LPC(16:3)	GP	LPC	1.6362	0.0002	0.0057	3.5628	1.8330	1.448	0.006	0.039	3.993	1.998
PE(P‐15:0_20:3)	GP	PE‐P	1.3359	0.0038	0.0193	3.4077	1.7688	1.373	0.011	0.055	3.043	1.605
Cer(t18:1/25:0)	SP	Cer‐NP	1.1970	0.0003	0.0057	3.3677	1.7518	1.436	0.000	0.010	2.937	1.554
Cer(d20:1/24:2(2OH))	SP	Cer‐AS	1.0005	0.0059	0.0257	3.2041	1.6799	1.243	0.006	0.038	3.193	1.675
Carnitine C22:3	FA	CAR	1.3209	0.0045	0.0216	3.1186	1.6409	1.629	0.002	0.023	3.429	1.778
PE(P‐18:1_18:2)	GP	PE‐P	1.5459	0.0005	0.0070	0.3424	−1.5462	1.504	0.006	0.038	0.472	−1.084
LPA(18:4)	GP	LPA	1.2662	0.0037	0.0193	0.3438	−1.5406	1.292	0.021	0.079	0.443	−1.174
PE(O‐18:3_18:2)	GP	PE‐O	1.5042	0.0005	0.0066	0.3458	−1.5319	1.697	0.001	0.018	0.415	−1.268
PE(P‐18:2_18:2)	GP	PE‐P	1.5581	0.0004	0.0061	0.3526	−1.5040	1.586	0.002	0.025	0.448	−1.158
PE(P‐18:2_20:5)	GP	PE‐P	1.2377	0.0065	0.0275	0.3779	−1.4038	1.409	0.011	0.055	0.477	−1.069
PE(P‐18:3_20:4)	GP	PE‐P	1.4094	0.0032	0.0179	0.3802	−1.3952	1.552	0.004	0.032	0.419	−1.256
PC(O‐16:1_22:6)	GP	PC‐O	1.3010	0.0035	0.0189	0.3819	−1.3887	1.799	0.000	0.010	0.358	−1.480
PE(16:2_18:2)	GP	PE	1.5107	0.0008	0.0079	0.4124	−1.2781	1.636	0.002	0.022	0.451	−1.148
PG(21:1_20:4)	GP	PG	1.5100	0.0037	0.0193	0.4141	−1.2719	2.006	0.000	0.003	0.394	−1.342
PI(18:1_22:6)	GP	PI	1.7203	0.0000	0.0018	0.4194	−1.2537	2.000	0.000	0.003	0.451	−1.148

## 4 Discussion

Obesity has been linked to the development of several cancer types, including pancreatic cancer [5, 35]. Pancreatic cancer is the fourth leading cause of cancer deaths in the US and is predicted to rise to second by 2030 [36]. Several risk factors have been identified as potential contributors to pancreatic cancer development, notably the medical conditions of obesity, diabetes, and chronic pancreatitis. In mice, exposure to PFOA leads to diabetic outcomes and pancreatic cancer [9, 10, 24], and in humans, epidemiologic studies have linked PFOA exposure to type 1, type 2, and gestational diabetes, obesity, and pancreatic cancer [12, 13, 21, 23, 37, 38]. PFOA is detectable in nearly all of the US population, with mean serum levels ~4 ng/mL and a predicted half‐life of ~4 years, and increased levels have been correlated with metabolic perturbations and cancer. Thus, determining the mechanism through which PFOA elicits these effects is an important public health issue. In this study, we report the comprehensive lipidomic analysis of pancreata from untreated KC mice and KC mice exposed to 1 and 5 ppm PFOA. Overall, we observed differential expression of 307 lipids common to both the 1 and 5 ppm PFOA‐exposed KC mice groups, with 137 individual lipid species upregulated and 170 individual lipid species downregulated (Table S6).

In our study, the lipid species that exhibited the greatest fold change in both the 1 and 5 ppm KC group versus CTL KC group was the eicosanoid TXB2. Eicosanoids are fatty acids derived from arachidonic acid (AA) that participate in tumor growth and metastasis through effects on proliferation, survival, differentiation, and inflammation [39]. The synthesis of TXB2 begins with AA generation which occurs by enzymatic cleavage of membrane phospholipids by phospholipase A2 (PLA2). AA is then metabolized by COX‐1 and/or COX‐2 enzymes to generate PGH2 which is metabolized to thromboxane A2 (TXA2), a potent pro‐inflammatory eicosanoid, by thromboxane synthase (TBXA2S) [40]. TXA2 is unstable in aqueous solutions and is spontaneously hydrolyzed to TXB2 (*t*_1/2_ ~ 30 s). As such, TXB2 is often used as a biomarker for TXA2 [41]. While originally identified for its role in platelet aggregation, increasing importance and involvement of AA metabolism and TXA2 in obesity and cancer has been demonstrated. HFD‐induced obesity was accompanied by inflammation, and insulin resistance that was associated with increased secretion of TXA2 in adipose tissue [42]. TXA2 binds to TXA2 receptors (TP), which are G‐protein coupled receptors expressed in most cell types including pancreatic cells [43]. Multiple studies have linked TXA2/TXB2, TBXA2S and TP to critical steps in cancer progression including tumor cell motility, invasion, proliferation, and therapeutic resistance [44]. Further, TXA2 has been implicated in angiogenesis observed during tumor growth and chronic inflammation [45]. TXB2 levels were elevated in pancreatic cancer tissue as was TBXA2S in high‐grade PanIN lesions [46], and high TP levels were associated with poor prognosis and reductions in median disease‐free survival in several cancers including pancreatic cancer [47]. As several of the most downregulated lipid species common to both the 1 and 5 ppm PFOA KC groups contain AA moieties, such as PE(P‐18:3_20:4) and PG(21:1_20:4) (Table 2), our data suggest that the increased TXB2 levels we observe may be derived in part by cleavage of membrane phospholipids by PLA2. In pancreatic cancer patients, expression of group II and IV PLA2s were increased compared to controls [48] and due to high expression and activity in serum and cancer cells, soluble PLA2 has been used as a diagnostic marker in pancreatic cancer [49, 50]. Evaluation of the expression and activity of PLA2 family members in the pancreas of PFOA‐exposed mice may help to further identify the source of increased TXB2 levels and understand its contribution to pancreatic cancer promotion.

Identification of the top upregulated lipids stratified by FC in PFOA‐exposed KC mice revealed increased levels of CAR species, particularly those attached to long‐chain (LCAC, C13‐C20) or very long‐chain FA moieties (> C21) (Tables 2 and S6). We observed upregulation of 22 individual LCACs in common between both 1 and 5 ppm PFOA‐exposed KC mice, with C16:0 and C18:1 exhibiting the highest levels (Table S6). Impaired FA oxidation along with tissue lipid accumulation are key elements of the pathophysiology of obesity, T2D, and insulin resistance [51, 52]. Newly diagnosed T2D patients exhibited increased LCAC levels compared to controls [53]. Additionally, plasma LCACs were increased in both obese nondiabetic and obese T2D compared to lean patients [52].

The main function of LCACs is to facilitate the transport of long‐chain FAs into the mitochondria to undergo β oxidation (FAO). LCACs are synthesized by carnitine palmitoyl transferase 1 (CPT1) on the outer mitochondrial membrane followed by translocation across the inner mitochondrial membrane. LCACs are then converted back to their acyl‐CoA forms by CPT2 allowing them to undergo FAO. Accumulation of LCACs could thus occur through upregulation of CPT1 expression or activity or downregulation of CPT2 expression or activity. In hepatocellular cancer (HCC), accumulation of LCACs in serum and liver was seen in HFD‐fed mice and in human HCC, a finding attributed to the downregulation of CPT2 expression [25, 54]. Further, levels of LCACs (AC14:1 and AC18:1) progressively increased concomitant with fibrosis in patients with non‐alcoholic fatty liver disease (NAFLD) and HCC [55]. CPT1, the rate‐limiting enzyme in FAO, is expressed as three isoforms, CPT1A, B, and C that differ in their tissue abundance and catalytic activity. Overexpression of CPT1A was observed in PDAC tissues and increased expression correlated with shorter survival time [56]. Overexpression of CPT1A in pancreatic cancer cells promoted cell proliferation, migration and glycolytic pathway activity, while downregulation of CPT1A reversed these effects [56]. Upregulation of the CPT1C isoform is seen in multiple cancer types including breast, lung, gastric and pancreatic cancer where it has been shown to promote cell proliferation and survival under conditions of metabolic stress and hypoxia [57, 58, 59, 60].

Systematic reviews of metabolomic studies in humans have shown correlations of PFAS levels with increases in fatty acids, carnitines, and ACs [61, 62]. Workers with occupational PFAS exposure had significantly higher plasma AC18:1 and AC18:2 levels indicative of mitochondrial FAO disruption [63]. In a study of young adults with 82% overweight or obese participants, higher PFOA levels were associated with glucose intolerance and higher AC levels suggesting that altered PFOA‐mediated FAO may contribute to impaired glucose metabolism [38]. In a metabolome‐wide association study of chemical exposures and chronic disease, increased carnitine/AC levels were associated with PFOA exposure [64]. In mice, exposure to PFOA led to liver injury with LCAC accumulation in hepatic tissue, indicating a block in FAO [65, 66]. Whether altered mRNA or protein levels of CPT1A, 1B, 1C or CPT2 or global mitochondrial dysfunction is the mechanism for PFOA‐induced LCAC accumulation in the pancreas of KC mice is currently under evaluation.

Ceramides are sphingolipids that serve both structural and functional roles within cell membranes, contributing to membrane integrity and fluidity, while also playing a crucial signaling role in various metabolic pathways. Ceramides are composed of a sphingoid base attached to a single FA, which, depending on the FA chain length and subcellular distribution, can regulate cell growth, differentiation, or apoptosis, with C6 ceramides promoting cell death and longer chains, such as C16, associated with cell proliferation [67]. In this study, we find that ceramides are among the most upregulated lipid groups (Table 2), with 15 individual long‐chain Cer species upregulated in common in the pancreas of both the 1 and 5 ppm treated KC mice groups (Table S6). In obesity, alterations in endogenous ceramide turnover and deregulation of metabolic pathways that control normal ceramide synthesis lead to accumulation of ceramides in serum and tissues, including liver, muscle, heart, and pancreas [26]. In pancreatic cancer patients with lymph node metastasis, significantly elevated tumor tissue levels of C16:0 and C24:1 ceramide were observed [68], that are also seen in the pancreas of PFOA‐exposed KC mice (Table 2).

We also observe that PFOA exposure leads to increased levels of ceramide‐1‐phosphate (CerP), which is synthesized from ceramide by the action of ceramide kinase (CERK) and exhibits protumorigenic and proinflammatory effects [69]. CerP was shown to regulate the migration and invasion of multiple cancer cell types including pancreatic cancer cells [69] and overexpression of CERK enhanced pancreatic cancer cell migration [70]. PDAC cells also secrete extracellular vesicles that contain CerP, which was shown to promote pancreatic cancer stem cell motility [71].

In conclusion, we report significant changes in lipid expression in the pancreas of KC mice following exposure to 1 and 5 ppm PFOA, with upregulation of lipid species within the CAR and Cer groups and downregulation of lipids in the PC, PE, and PS groups. We have previously shown that PFOA exposure of pancreatic acinar cells triggers ER and oxidative stress in vitro and oxidative stress in the pancreas in vivo [31, 72]. Excess ceramide levels can also induce ER stress, impair mitochondrial function [73], and induce inflammation [74]. CerP has been shown to activate cPLA2 leading to formation of AA and eicosanoid synthesis [75] further connecting the PFOA‐mediated lipid alterations to inflammation. Together, our data show that PFOA elicits lipidomic changes indicative of mitochondrial dysfunction and inflammation that may play a key role in the promotion of pancreatic cancer.

## Author Contributions

Barbara A. Hocevar and Lisa M. Kamendulis conceived and designed the experiments, performed the experiments, and data analysis. Both authors contributed to the article and approved the submitted version.

## Funding

Support for this work was provided, in part, by the Indiana University Simon Comprehensive Cancer Center and the Indiana University School of Public Health—Bloomington.

## Conflicts of Interest

The authors declare no conflicts of interest.

## Supporting information


**Table S1:** Serum PFOA levels following gavage and drinking water exposure.
**Table S2:** Internal standards.
**Table S3:** Lipid classes identified in KC mice.
**Table S4:** Differentially expressed lipids between 1 ppm PFOA and control KC mice, sorted by fold‐change.
**Table S5:** Differentially expressed lipids between 5 ppm PFOA and control KC mice, sorted by fold‐change.
**Table S6:** Differentially expressed lipids common to both 1 and 5 ppm PFOA versus control KC mice.
**Figure S1:** Quality control (QC) metrics.
**Figure S2:** OPLS‐DA model validation.

## Data Availability

The data that support the findings of this study are provided in the article or in Supporting Information of this article. Raw data will be made available upon request.
