# Supplementary material for: PFOA Exposure Elicits Quantitative Lipidomic Changes in the Pancreas in a Mouse Model of Pancreatic Cancer
Source: Environ Toxicol. 2026 Apr 24;41(12):1836–51. doi: 10.1002/tox.70109 (PMC13749286; doi:10.1002/tox.70109)
Supplement: Supplementary file 1 [file TOX-41-1836-s001.docx]

**Supplemental Table S1: Gavage versus Drinking Water PFOA dose determination**

| **Table S1. Serum PFOA Levels Following Gavage and Drinking Water Exposure** | | | |
| --- | --- | --- | --- |
| **Gavage**  **(mg/Kg)** | Serum (µg/ml) | **Water (ppm)** | Serum (µg/ml) |
| 0 | 0.005 | 0 | 0.005 |
| 0.5 | 4.16 | 5 | 17.79 |
| 2.5 | 28.88 | 10 | 31.38 |
| 5.0 | 47.98 | 20 | 64.90 |
| 1.0 | 10.44* | 3.1* | 10.44 |
| *Calculated by solving the equation of the line  n = 4/5 mice per group | | | |

**Supplemental Table S2: Internal Standards**

| **Index** | **Internal Standard Compounds** | **Concentration**  **(uM）** | **Class** |
| --- | --- | --- | --- |
| 9 | GCDCA-d4 | 0.4 | BA |
| 7 | CAR(16:0)-d3 | 0.04 | CAR |
| 8 | CE(18:1)-d7 | 2 | CE |
| 11 | Cer(d18:1-d7/18:0) | 0.1 | Cer |
| 25 | Cer(d18:1/15:0)-d7 | 0.1 | Cer |
| 48 | Cer(d18:1-d7/24:0) | 0.1 | Cer |
| 12 | CerP(d18:1/8:0) | 0.4 | CerP |
| 2 | cholesterol-d7 | 5 | Cho |
| 6 | CoQ10-d9 | 0.4 | CoQ |
| 15 | DG(17:0/17:0)-d5 | 0.5 | DG |
| 1 | 5S-HETE-d8 | 0.04 | Eicosanoid |
| 5 | FFA(16:0)-d31 | 2 | FFA |
| 47 | Arachidonic Acid-d8 | 0.5 | FFA |
| 13 | HexCer(d18:1-d5/18:0) | 0.4 | HexCer |
| 49 | LPA(17:0) | 0.5 | LPA |
| 17 | LPC(16:0)-d31 | 0.2 | LPC |
| 31 | LPC(15:0)-d5 | 0.2 | LPC |
| 32 | LPC(17:0)-d5 | 0.2 | LPC |
| 37 | LPC(18:1)-d7 | 0.2 | LPC |
| 27 | LPE(15:0)-d5 | 0.2 | LPE |
| 41 | LPE(18:1)-d7 | 0.2 | LPE |
| 38 | LPG(17:1) | 0.2 | LPG |
| 42 | LPG(15:0)-d5 | 0.2 | LPG |
| 3 | LPI(17:1) | 0.2 | LPI |
| 18 | LPS(17:1) | 0.2 | LPS |
| 39 | MG(18:1-d7) | 0.5 | MG |
| 50 | PA(17:0/17:0) | 0.5 | PA |
| 19 | PC(16:0-d31/18:1) | 2 | PC |
| 24 | PC(16:0/16:0)-d9 | 0.2 | PC |
| 26 | PC(14:0/14:0)-d9 | 0.2 | PC |
| 43 | PC(15:0/18:1(d7)) | 0.2 | PC |
| 20 | PE(16:0-d31/18:1) | 0.2 | PE |
| 33 | PE(17:0/18:1)-d5 | 0.2 | PE |
| 34 | PE(17:0/14:1)-d5 | 0.2 | PE |
| 40 | PE(17:0-22:4)-d5 | 0.2 | PE |
| 44 | PE(15:0/18:1(d7)) | 0.2 | PE |
| 21 | PG(16:0/d31/18:1) | 0.1 | PG |
| 45 | PG(15:0/18:1(d7)) | 0.1 | PG |
| 4 | PI(16:0-d31/18:1) | 0.2 | PI |
| 51 | PMeOH(16:0/16:0) | 0.5 | PMeOH |
| 22 | PS(16:0(d31)/18:1) | 0.4 | PS |
| 23 | PS(16:0/16:0)-d9 | 0.4 | PS |
| 46 | PS(15:0/18:1(d7)) | 0.4 | PS |
| 14 | SM(d18:1-d9/15:0) | 0.2 | SM |
| 28 | SM(d18:1/20:1)-d9 | 0.2 | SM |
| 29 | SM(d18:1/22:1)-d9 | 0.2 | SM |
| 35 | SM(d18:1/18:1)-d9 | 0.2 | SM |
| 10 | SPH(18:1)-d7 | 0.04 | SPH |
| 16 | TG(17:0/17:1/17:0)-d5 | 2 | TG |
| 30 | TG(16:0-15:1-16:0)-d5 | 0.2 | TG |
| 36 | TG(14:0/16:1/14:0)-d5 | 0.2 | TG |

| **Supplemental Table S3. Lipid Classes Identified in KC Mice** | | | | | | | | | | |
| --- | --- | --- | --- | --- | --- | --- | --- | --- | --- | --- |
|  |  | Control | |  | 1ppm PFOA | |  | 5ppm PFOA | |  |
|  | # Found | Mean | SD |  | Mean | SD |  | Mean | SD |  |
| all | 1450 | 78322.9 | 4768.6 |  | 72827.6 | 12007.6 |  | **70032.6** | **6625.9** |  |
| BA | 8 | 3.4 | 0.7 |  | 4.0 | 1.5 |  | 4.3 | 1.9 |  |
| BMP | 1 | 6.5 | 3.4 |  | **10.6** | **3.0** |  | 5.9 | 3.0 |  |
| CAR | 58 | 12.8 | 2.5 |  | **20.1** | **4.4** |  | **20.5** | **5.0** |  |
| Cer-AP | 33 | 352.4 | 40.4 |  | **1477.6** | **1331.2** |  | **619.0** | **227.7** |  |
| Cer-AS | 105 | 1071.0 | 254.6 |  | 3523.0 | 3153.1 |  | 1505.6 | 560.2 |  |
| Cer-NDS | 10 | 181.8 | 58.9 |  | 176.0 | 35.8 |  | 171.0 | 32.4 |  |
| Cer-NP | 29 | 853.7 | 207.9 |  | **1745.0** | **990.1** |  | **1386.1** | **441.7** |  |
| Cer-NS | 29 | 4636.0 | 1757.4 |  | 3867.9 | 2199.7 |  | 4341.5 | 1238.6 |  |
| CerP | 5 | 388.8 | 65.6 |  | **528.8** | **103.3** |  | **584.0** | **134.3** |  |
| Cholesterol | 1 | 7040.7 | 492.2 |  | 6949.8 | 987.9 |  | 6777.3 | 756.9 |  |
| CoQ | 3 | 149.2 | 26.7 |  | **110.0** | **23.4** |  | **96.1** | **19.1** |  |
| DG | 27 | 268.8 | 342.8 |  | 1518.5 | 2157.4 |  | 349.3 | 492.4 |  |
| DG-O | 1 | 15.6 | 24.9 |  | 97.8 | 144.8 |  | 10.8 | 22.1 |  |
| Eicosanoid | 19 | 8.6 | 6.3 |  | 14.1 | 9.3 |  | **16.7** | **6.2** |  |
| FFA | 43 | 1731.4 | 1350.5 |  | 2665.5 | 2227.4 |  | 2042.6 | 1273.7 |  |
| HexCer-AP | 25 | 1116.6 | 608.9 |  | 1250.0 | 597.2 |  | 1339.5 | 473.9 |  |
| HexCer-NS | 25 | 1778.5 | 713.6 |  | 2374.2 | 904.0 |  | 1670.8 | 807.2 |  |
| LNAPE | 15 | 262.7 | 35.7 |  | **142.1** | **62.6** |  | **190.1** | **72.3** |  |
| LPA | 8 | 35.5 | 6.5 |  | **23.5** | **4.2** |  | **25.1** | **10.5** |  |
| LPC | 44 | 439.1 | 61.2 |  | 404.5 | 58.3 |  | 530.1 | 122.6 |  |
| LPC-O | 14 | 6.4 | 1.6 |  | **13.3** | **4.1** |  | **12.0** | **5.1** |  |
| LPE | 29 | 9477.0 | 987.0 |  | **7997.8** | **1225.8** |  | 8610.7 | 2664.7 |  |
| LPE-P | 5 | 546.3 | 115.1 |  | **269.3** | **100.3** |  | 467.4 | 582.0 |  |
| LPG | 13 | 9.2 | 0.4 |  | 7.9 | 2.0 |  | 9.5 | 3.7 |  |
| LPI | 14 | 213.9 | 16.2 |  | **138.4** | **59.7** |  | **150.6** | **30.7** |  |
| LPS | 13 | 70.9 | 16.1 |  | 39.7 | 15.3 |  | 44.6 | 29.4 |  |
| MG | 7 | 42.1 | 32.0 |  | 68.0 | 57.7 |  | 48.5 | 20.9 |  |
| MGDG | 2 | 26.2 | 7.7 |  | 17.6 | 8.5 |  | 20.2 | 6.4 |  |
| PC | 103 | 12813.6 | 1218.1 |  | **8885.8** | **2254.7** |  | **10470.9** | **1738.9** |  |
| PC-O | 24 | 259.7 | 20.8 |  | 219.8 | 32.3 |  | 232.0 | 48.4 |  |
| PE | 115 | 3472.2 | 232.9 |  | **2551.5** | **392.8** |  | **2956.8** | **506.5** |  |
| PE-O | 87 | 947.1 | 70.2 |  | **685.1** | **118.8** |  | **746.9** | **136.8** |  |
| PE-P | 53 | 3967.3 | 931.0 |  | **2425.9** | **668.1** |  | **2555.4** | **792.8** |  |
| PG | 95 | 281.1 | 16.5 |  | **190.4** | **36.3** |  | **201.7** | **28.4** |  |
| PI | 67 | 1088.6 | 127.5 |  | **675.4** | **204.0** |  | **770.9** | **206.8** |  |
| PMeOH | 5 | 5.0 | 0.2 |  | 4.9 | 0.2 |  | 5.1 | 0.6 |  |
| PS | 110 | 23988.6 | 3528.5 |  | **17276.7** | **3967.5** |  | **19641.3** | **4349.9** |  |
| SHexCer | 2 | 75.7 | 65.9 |  | 136.7 | 132.9 |  | 52.1 | 34.4 |  |
| SM | 31 | 122.3 | 11.5 |  | 130.0 | 15.8 |  | 123.5 | 9.6 |  |
| SPH | 3 | 3.1 | 0.8 |  | **2.1** | **0.3** |  | **2.3** | **0.5** |  |
| TG | 169 | 553.5 | 722.5 |  | 4188.1 | 5312.6 |  | 1223.8 | 1881.3 |  |
| Units are expressed as nmol/g tissue. Bolded = significantly different from control, p < 0.05 | | | | | | | | | |  |

| **Supplemental Table S4** | |  |  |  |  | |  | |  | |  | | |  |
| --- | --- | --- | --- | --- | --- | --- | --- | --- | --- | --- | --- | --- | --- | --- |
| **Differentially Expressed Lipids Between 1 ppm PFOA and Control KC Mice, sorted by Fold-Change** | | | | | | | | | | | | |  |  |
|  |  |  |  |  |  | |  | |  | |  | | |  |
| Index | Compounds | Class I | Class II | VIP | | Pvalue | | FDR | | Fold_Change | | Log2FC | | |
| LIPID-N-0063 | TXB2 | FA | Eicosanoid | 1.4229 | 0.0003 | | 0.0057 | | 12.1561 | | 3.6036 | | |  |
| LIPID-P-2597 | Cer(d25:3/34:0(2OH)) | SP | Cer-AS | 1.0665 | 0.0356 | | 0.0922 | | 8.6042 | | 3.1050 | | |  |
| LIPID-P-1305 | Carnitine C22:5 | FA | CAR | 1.3177 | 0.0086 | | 0.0342 | | 5.0883 | | 2.3472 | | |  |
| LIPID-N-0560 | PS(16:0_16:0) | GP | PS | 1.3940 | 0.0006 | | 0.0070 | | 4.6549 | | 2.2188 | | |  |
| LIPID-P-1290 | Carnitine C17:1 | FA | CAR | 1.4771 | 0.0009 | | 0.0083 | | 4.5456 | | 2.1845 | | |  |
| LIPID-P-2556 | Cer(d20:1/24:1(2OH)) | SP | Cer-AS | 1.2846 | 0.0012 | | 0.0099 | | 4.1744 | | 2.0616 | | |  |
| LIPID-P-2555 | Cer(d20:1/24:0(2OH)) | SP | Cer-AS | 1.2747 | 0.0012 | | 0.0099 | | 3.9775 | | 1.9919 | | |  |
| LIPID-P-2433 | Cer(t17:2/24:1) | SP | Cer-NP | 1.1561 | 0.0059 | | 0.0257 | | 3.6598 | | 1.8718 | | |  |
| LIPID-P-2266 | HexCer(d20:2/24:0) | SP | HexCer-NS | 1.0207 | 0.0003 | | 0.0057 | | 3.5970 | | 1.8468 | | |  |
| LIPID-P-1931 | LPC(16:3) | GP | LPC | 1.6362 | 0.0002 | | 0.0057 | | 3.5628 | | 1.8330 | | |  |
| LIPID-P-0045 | Carnitine C18:1-OH | FA | CAR | 1.4028 | 0.0003 | | 0.0057 | | 3.5236 | | 1.8171 | | |  |
| LIPID-P-0646 | PE(P-15:0_20:3) | GP | PE-P | 1.3359 | 0.0038 | | 0.0193 | | 3.4077 | | 1.7688 | | |  |
| LIPID-P-2552 | Cer(d20:1/22:0(2OH)) | SP | Cer-AS | 1.2214 | 0.0022 | | 0.0133 | | 3.3875 | | 1.7602 | | |  |
| LIPID-P-2453 | Cer(t18:1/25:0) | SP | Cer-NP | 1.1970 | 0.0003 | | 0.0057 | | 3.3677 | | 1.7518 | | |  |
| LIPID-P-0051 | Carnitine C18:2-OH | FA | CAR | 1.4060 | 0.0003 | | 0.0057 | | 3.3435 | | 1.7414 | | |  |
| LIPID-P-2255 | HexCer(d19:2/24:0) | SP | HexCer-NS | 1.1893 | 0.0006 | | 0.0070 | | 3.3391 | | 1.7394 | | |  |
| LIPID-P-2262 | HexCer(d20:1/24:0) | SP | HexCer-NS | 1.2430 | 0.0003 | | 0.0057 | | 3.3094 | | 1.7266 | | |  |
| LIPID-P-0730 | SM(d18:0/16:0) | SP | SM | 1.6351 | 0.0001 | | 0.0046 | | 3.2733 | | 1.7108 | | |  |
| LIPID-P-0550 | PC(O-18:1_18:2) | GP | PC-O | 1.7215 | 0.0000 | | 0.0036 | | 3.2220 | | 1.6880 | | |  |
| LIPID-P-2557 | Cer(d20:1/24:2(2OH)) | SP | Cer-AS | 1.0005 | 0.0059 | | 0.0257 | | 3.2041 | | 1.6799 | | |  |
| LIPID-P-2553 | Cer(d20:1/23:0(2OH)) | SP | Cer-AS | 1.0795 | 0.0012 | | 0.0099 | | 3.1580 | | 1.6590 | | |  |
| LIPID-P-2422 | Cer(t17:2/16:0) | SP | Cer-NP | 1.1116 | 0.0289 | | 0.0788 | | 3.1376 | | 1.6497 | | |  |
| LIPID-P-1301 | Carnitine C22:3 | FA | CAR | 1.3209 | 0.0045 | | 0.0216 | | 3.1186 | | 1.6409 | | |  |
| LIPID-N-0055 | PGD2 | FA | Eicosanoid | 1.1878 | 0.0022 | | 0.0133 | | 3.1168 | | 1.6401 | | |  |
| LIPID-N-0812 | PE(O-16:0_22:5) | GP | PE-O | 1.0758 | 0.0093 | | 0.0358 | | 3.0584 | | 1.6128 | | |  |
| LIPID-P-0306 | HexCer(d18:1/24:1) | SP | HexCer-NS | 1.4863 | 0.0003 | | 0.0057 | | 2.9655 | | 1.5683 | | |  |
| LIPID-P-1298 | Carnitine C16:3 | FA | CAR | 1.4059 | 0.0012 | | 0.0099 | | 2.9298 | | 1.5508 | | |  |
| LIPID-P-2260 | HexCer(d20:1/23:0) | SP | HexCer-NS | 1.2428 | 0.0003 | | 0.0057 | | 2.9290 | | 1.5504 | | |  |
| LIPID-P-0365 | LPC(26:0) | GP | LPC | 1.5064 | 0.0006 | | 0.0070 | | 2.9086 | | 1.5403 | | |  |
| LIPID-P-2113 | Cer(d20:2/24:0) | SP | Cer-NS | 1.2318 | 0.0006 | | 0.0070 | | 2.8978 | | 1.5349 | | |  |
| LIPID-P-2423 | Cer(t17:2/16:1) | SP | Cer-NP | 1.1300 | 0.0140 | | 0.0483 | | 2.8673 | | 1.5197 | | |  |
| LIPID-N-1352 | PS(20:2_22:4) | GP | PS | 1.0033 | 0.0037 | | 0.0193 | | 2.8626 | | 1.5173 | | |  |
| LIPID-N-0793 | PE(O-18:0_20:4) | GP | PE-O | 1.1047 | 0.0289 | | 0.0788 | | 2.8554 | | 1.5137 | | |  |
| LIPID-P-1303 | Carnitine C22:4 | FA | CAR | 1.3659 | 0.0003 | | 0.0057 | | 2.8482 | | 1.5100 | | |  |
| LIPID-P-0302 | HexCer(d18:1/16:1) | SP | HexCer-NS | 1.5174 | 0.0005 | | 0.0066 | | 2.8357 | | 1.5037 | | |  |
| LIPID-N-0020 | 13-HOTrE | FA | Eicosanoid | 1.1640 | 0.0159 | | 0.0538 | | 2.8241 | | 1.4978 | | |  |
| LIPID-P-0046 | Carnitine C10:2 | FA | CAR | 1.2936 | 0.0022 | | 0.0133 | | 2.7968 | | 1.4838 | | |  |
| LIPID-P-0040 | Carnitine C16:1-OH | FA | CAR | 1.3503 | 0.0003 | | 0.0057 | | 2.7894 | | 1.4799 | | |  |
| LIPID-P-2442 | Cer(t18:0/16:0) | SP | Cer-NP | 1.0814 | 0.0059 | | 0.0257 | | 2.7788 | | 1.4745 | | |  |
| LIPID-P-2143 | HexCer(t17:2/16:0(2OH)) | SP | HexCer-AP | 1.3573 | 0.0017 | | 0.0121 | | 2.7758 | | 1.4729 | | |  |
| LIPID-P-2258 | HexCer(d20:1/22:0) | SP | HexCer-NS | 1.3692 | 0.0003 | | 0.0057 | | 2.7537 | | 1.4614 | | |  |
| LIPID-P-1282 | Carnitine C17:0 | FA | CAR | 1.6010 | 0.0001 | | 0.0046 | | 2.6990 | | 1.4324 | | |  |
| LIPID-P-0037 | Carnitine C14:1 | FA | CAR | 1.3373 | 0.0042 | | 0.0207 | | 2.6829 | | 1.4238 | | |  |
| LIPID-N-1326 | PS(20:0_22:4) | GP | PS | 1.1471 | 0.0093 | | 0.0358 | | 2.6675 | | 1.4155 | | |  |
| LIPID-P-0404 | LPC(O-20:2) | GP | LPC-O | 1.2883 | 0.0022 | | 0.0133 | | 2.6508 | | 1.4064 | | |  |
| LIPID-P-0732 | SM(d18:0/18:0) | SP | SM | 1.3046 | 0.0012 | | 0.0099 | | 2.6206 | | 1.3899 | | |  |
| LIPID-P-0764 | SM(d18:2/23:1) | SP | SM | 1.4363 | 0.0022 | | 0.0133 | | 2.6160 | | 1.3873 | | |  |
| LIPID-N-0392 | PE(18:0_22:4) | GP | PE | 1.3825 | 0.0012 | | 0.0099 | | 2.5862 | | 1.3708 | | |  |
| LIPID-P-0042 | Carnitine C18:1 | FA | CAR | 1.6197 | 0.0001 | | 0.0046 | | 2.5611 | | 1.3568 | | |  |
| LIPID-P-0731 | SM(d18:0/17:0) | SP | SM | 1.4249 | 0.0012 | | 0.0099 | | 2.5582 | | 1.3551 | | |  |
| LIPID-P-0038 | Carnitine C14:1-OH | FA | CAR | 1.2245 | 0.0140 | | 0.0483 | | 2.5559 | | 1.3538 | | |  |
| LIPID-P-0748 | SM(d20:0/24:1) | SP | SM | 1.5644 | 0.0001 | | 0.0054 | | 2.5418 | | 1.3459 | | |  |
| LIPID-P-2263 | HexCer(d20:1/24:1) | SP | HexCer-NS | 1.1238 | 0.0093 | | 0.0358 | | 2.5376 | | 1.3435 | | |  |
| LIPID-P-1316 | PE(P-15:0_18:1) | GP | PE-P | 1.1797 | 0.0022 | | 0.0133 | | 2.5345 | | 1.3417 | | |  |
| LIPID-P-2099 | Cer(d19:2/24:0) | SP | Cer-NS | 1.2061 | 0.0022 | | 0.0133 | | 2.5273 | | 1.3376 | | |  |
| LIPID-P-0043 | Carnitine C20:1 | FA | CAR | 1.6201 | 0.0001 | | 0.0050 | | 2.5154 | | 1.3308 | | |  |
| LIPID-P-0739 | SM(d18:1/17:0) | SP | SM | 1.2372 | 0.0006 | | 0.0070 | | 2.5116 | | 1.3286 | | |  |
| LIPID-N-0050 | 14(S)-HDHA | FA | Eicosanoid | 1.2038 | 0.0109 | | 0.0412 | | 2.5042 | | 1.3243 | | |  |
| LIPID-P-0022 | Carnitine C16-OH | FA | CAR | 1.3857 | 0.0012 | | 0.0099 | | 2.5032 | | 1.3238 | | |  |
| LIPID-N-0732 | PE(O-16:0_20:0) | GP | PE-O | 1.6532 | 0.0001 | | 0.0040 | | 2.4867 | | 1.3142 | | |  |
| LIPID-P-0018 | Carnitine C15:0 | FA | CAR | 1.6297 | 0.0001 | | 0.0040 | | 2.4519 | | 1.2939 | | |  |
| LIPID-P-0039 | Carnitine C16:1 | FA | CAR | 1.5206 | 0.0006 | | 0.0070 | | 2.4492 | | 1.2923 | | |  |
| LIPID-P-0403 | LPC(O-18:2) | GP | LPC-O | 1.4113 | 0.0020 | | 0.0133 | | 2.4461 | | 1.2905 | | |  |
| LIPID-P-0400 | LPC(O-18:1) | GP | LPC-O | 1.3476 | 0.0052 | | 0.0241 | | 2.4183 | | 1.2740 | | |  |
| LIPID-N-0823 | PE(O-16:1_24:4) | GP | PE-O | 1.4762 | 0.0011 | | 0.0095 | | 2.4131 | | 1.2709 | | |  |
| LIPID-P-0013 | Carnitine C10:0 | FA | CAR | 1.3591 | 0.0023 | | 0.0142 | | 2.4020 | | 1.2642 | | |  |
| LIPID-N-0797 | PE(O-20:0_20:4) | GP | PE-O | 1.1718 | 0.0093 | | 0.0358 | | 2.3957 | | 1.2604 | | |  |
| LIPID-P-0035 | Carnitine C12:1 | FA | CAR | 1.1245 | 0.0205 | | 0.0631 | | 2.3892 | | 1.2565 | | |  |
| LIPID-N-0796 | PE(O-18:0_22:4) | GP | PE-O | 1.3023 | 0.0022 | | 0.0133 | | 2.3767 | | 1.2489 | | |  |
| LIPID-N-1042 | PC(15:1_22:4) | GP | PC | 1.2990 | 0.0003 | | 0.0057 | | 2.3705 | | 1.2452 | | |  |
| LIPID-N-0729 | PE(O-16:0_16:0) | GP | PE-O | 1.2325 | 0.0140 | | 0.0483 | | 2.3456 | | 1.2300 | | |  |
| LIPID-P-0538 | PC(O-18:1_16:0) | GP | PC-O | 1.2945 | 0.0012 | | 0.0099 | | 2.3452 | | 1.2297 | | |  |
| LIPID-N-0763 | PE(O-18:1_22:1) | GP | PE-O | 1.3295 | 0.0012 | | 0.0099 | | 2.3407 | | 1.2269 | | |  |
| LIPID-N-0816 | PE(O-17:1_22:4) | GP | PE-O | 1.3143 | 0.0093 | | 0.0358 | | 2.3399 | | 1.2265 | | |  |
| LIPID-P-0014 | Carnitine C12:0 | FA | CAR | 1.3504 | 0.0033 | | 0.0179 | | 2.3219 | | 1.2153 | | |  |
| LIPID-P-0395 | LPC(O-18:0) | GP | LPC-O | 1.3693 | 0.0032 | | 0.0176 | | 2.3058 | | 1.2053 | | |  |
| LIPID-P-1513 | Cer(t18:0/24:0(2OH)) | SP | Cer-AP | 1.0701 | 0.0140 | | 0.0483 | | 2.2950 | | 1.1985 | | |  |
| LIPID-P-0307 | HexCer(d18:1/26:1) | SP | HexCer-NS | 1.0880 | 0.0337 | | 0.0881 | | 2.2919 | | 1.1965 | | |  |
| LIPID-N-0017 | Taurocholicacid | ST | BA | 1.1208 | 0.0205 | | 0.0631 | | 2.2817 | | 1.1901 | | |  |
| LIPID-N-0792 | PE(O-16:0_22:4) | GP | PE-O | 1.4522 | 0.0006 | | 0.0070 | | 2.2797 | | 1.1888 | | |  |
| LIPID-N-0788 | PE(O-16:0_20:4) | GP | PE-O | 1.1588 | 0.0140 | | 0.0483 | | 2.2771 | | 1.1872 | | |  |
| LIPID-P-0050 | Carnitine C20:2 | FA | CAR | 1.6270 | 0.0001 | | 0.0046 | | 2.2474 | | 1.1683 | | |  |
| LIPID-N-0202 | PC(14:0_14:0) | GP | PC | 1.2373 | 0.0140 | | 0.0483 | | 2.2449 | | 1.1667 | | |  |
| LIPID-P-2145 | HexCer(t17:2/22:2(2OH)) | SP | HexCer-AP | 1.1539 | 0.0093 | | 0.0358 | | 2.2365 | | 1.1613 | | |  |
| LIPID-N-0742 | PE(O-18:0_20:1) | GP | PE-O | 1.3055 | 0.0062 | | 0.0265 | | 2.2027 | | 1.1393 | | |  |
| LIPID-P-2102 | Cer(d20:1/23:1) | SP | Cer-NS | 1.1190 | 0.0289 | | 0.0788 | | 2.2025 | | 1.1392 | | |  |
| LIPID-P-0394 | LPC(O-16:0) | GP | LPC-O | 1.4981 | 0.0007 | | 0.0074 | | 2.1686 | | 1.1168 | | |  |
| LIPID-P-0393 | LPC(O-14:0) | GP | LPC-O | 1.5095 | 0.0004 | | 0.0064 | | 2.1673 | | 1.1159 | | |  |
| LIPID-P-0397 | LPC(O-22:0) | GP | LPC-O | 1.2728 | 0.0048 | | 0.0228 | | 2.1659 | | 1.1150 | | |  |
| LIPID-P-1300 | Carnitine C20:3 | FA | CAR | 1.4530 | 0.0012 | | 0.0099 | | 2.1648 | | 1.1142 | | |  |
| LIPID-P-0033 | Carnitine C10:1 | FA | CAR | 1.3515 | 0.0040 | | 0.0201 | | 2.1552 | | 1.1078 | | |  |
| LIPID-N-0342 | PE(24:0_16:1) | GP | PE | 1.3754 | 0.0057 | | 0.0257 | | 2.1489 | | 1.1036 | | |  |
| LIPID-P-0433 | LPE(22:4) | GP | LPE | 1.6266 | 0.0001 | | 0.0046 | | 2.1462 | | 1.1018 | | |  |
| LIPID-N-1181 | PG(19:0_20:3) | GP | PG | 1.2087 | 0.0059 | | 0.0257 | | 2.1455 | | 1.1013 | | |  |
| LIPID-N-0345 | PE(24:0_18:1) | GP | PE | 1.3318 | 0.0072 | | 0.0301 | | 2.1361 | | 1.0950 | | |  |
| LIPID-P-0729 | SM(d18:0/14:0) | SP | SM | 1.5451 | 0.0005 | | 0.0070 | | 2.1356 | | 1.0947 | | |  |
| LIPID-N-0200 | LPS(22:5) | GP | LPS | 1.5893 | 0.0004 | | 0.0064 | | 2.1336 | | 1.0933 | | |  |
| LIPID-P-0566 | PC(O-18:0_22:6) | GP | PC-O | 1.4724 | 0.0018 | | 0.0126 | | 2.1249 | | 1.0874 | | |  |
| LIPID-N-0026 | 9,10-DiHOME | FA | Eicosanoid | 1.0140 | 0.0289 | | 0.0788 | | 2.1176 | | 1.0824 | | |  |
| LIPID-P-0401 | LPC(O-20:1) | GP | LPC-O | 1.2448 | 0.0205 | | 0.0631 | | 2.1123 | | 1.0788 | | |  |
| LIPID-N-0817 | PE(O-18:0_22:5) | GP | PE-O | 1.3203 | 0.0093 | | 0.0358 | | 2.1066 | | 1.0749 | | |  |
| LIPID-P-0551 | PC(O-18:0_20:3) | GP | PC-O | 1.3378 | 0.0050 | | 0.0235 | | 2.1039 | | 1.0731 | | |  |
| LIPID-P-0017 | Carnitine C14:0 | FA | CAR | 1.3228 | 0.0042 | | 0.0207 | | 2.1038 | | 1.0730 | | |  |
| LIPID-P-0431 | LPE(22:3) | GP | LPE | 1.6028 | 0.0002 | | 0.0055 | | 2.0963 | | 1.0679 | | |  |
| LIPID-P-0532 | PC(O-16:0_16:0) | GP | PC-O | 1.4731 | 0.0015 | | 0.0110 | | 2.0925 | | 1.0652 | | |  |
| LIPID-N-1324 | PS(20:0_20:4) | GP | PS | 1.0562 | 0.0205 | | 0.0631 | | 2.0923 | | 1.0651 | | |  |
| LIPID-P-0020 | Carnitine C14-OH | FA | CAR | 1.2882 | 0.0059 | | 0.0257 | | 2.0877 | | 1.0619 | | |  |
| LIPID-N-0561 | PS(18:0_16:0) | GP | PS | 1.5848 | 0.0001 | | 0.0046 | | 2.0829 | | 1.0586 | | |  |
| LIPID-N-0749 | PE(O-16:0_18:2) | GP | PE-O | 1.2659 | 0.0167 | | 0.0555 | | 2.0785 | | 1.0555 | | |  |
| LIPID-N-0759 | PE(O-16:1_22:1) | GP | PE-O | 1.3499 | 0.0042 | | 0.0206 | | 2.0654 | | 1.0464 | | |  |
| LIPID-N-0041 | 11(S)-HETE | FA | Eicosanoid | 1.2635 | 0.0093 | | 0.0358 | | 2.0574 | | 1.0408 | | |  |
| LIPID-N-1198 | PG(21:0_20:4) | GP | PG | 1.2662 | 0.0062 | | 0.0265 | | 2.0506 | | 1.0361 | | |  |
| LIPID-N-1108 | PE(22:5_22:6) | GP | PE | 1.2174 | 0.0205 | | 0.0631 | | 2.0350 | | 1.0250 | | |  |
| LIPID-P-0554 | PC(O-16:0_20:4) | GP | PC-O | 1.4832 | 0.0022 | | 0.0133 | | 2.0269 | | 1.0193 | | |  |
| LIPID-P-0533 | PC(O-18:0_16:0) | GP | PC-O | 1.5246 | 0.0006 | | 0.0070 | | 2.0197 | | 1.0142 | | |  |
| LIPID-N-0598 | PS(18:0_22:6) | GP | PS | 1.3928 | 0.0093 | | 0.0358 | | 2.0101 | | 1.0073 | | |  |
| LIPID-N-1118 | PG(16:0_21:0) | GP | PG | 1.4583 | 0.0006 | | 0.0070 | | 2.0069 | | 1.0050 | | |  |
| LIPID-P-1376 | PE(P-16:0_24:5) | GP | PE-P | 1.0361 | 0.0205 | | 0.0631 | | 2.0041 | | 1.0029 | | |  |
| LIPID-P-0555 | PC(O-18:0_20:4) | GP | PC-O | 1.3179 | 0.0289 | | 0.0788 | | 1.9959 | | 0.9970 | | |  |
| LIPID-P-0561 | PC(O-20:1_20:4) | GP | PC-O | 1.3323 | 0.0037 | | 0.0193 | | 1.9843 | | 0.9886 | | |  |
| LIPID-P-0275 | MG(17:0) | GL | MG | 1.0414 | 0.0205 | | 0.0631 | | 1.9789 | | 0.9847 | | |  |
| LIPID-P-0542 | PC(O-18:0_24:1) | GP | PC-O | 1.2384 | 0.0022 | | 0.0133 | | 1.9673 | | 0.9762 | | |  |
| LIPID-N-0751 | PE(O-17:1_18:1) | GP | PE-O | 1.1621 | 0.0012 | | 0.0099 | | 1.9633 | | 0.9733 | | |  |
| LIPID-P-0031 | Carnitine C8:1 | FA | CAR | 1.2008 | 0.0022 | | 0.0133 | | 1.9495 | | 0.9631 | | |  |
| LIPID-P-0015 | Carnitine C13:0 | FA | CAR | 1.4371 | 0.0013 | | 0.0100 | | 1.9401 | | 0.9561 | | |  |
| LIPID-P-0531 | PC(O-16:0_14:0) | GP | PC-O | 1.5510 | 0.0004 | | 0.0064 | | 1.9353 | | 0.9526 | | |  |
| LIPID-P-0025 | Carnitine C18-OH | FA | CAR | 1.3838 | 0.0029 | | 0.0163 | | 1.9252 | | 0.9450 | | |  |
| LIPID-P-1930 | LPC(24:2) | GP | LPC | 1.4954 | 0.0003 | | 0.0057 | | 1.9195 | | 0.9408 | | |  |
| LIPID-N-1057 | PE(18:0_22:1) | GP | PE | 1.3129 | 0.0059 | | 0.0257 | | 1.9188 | | 0.9402 | | |  |
| LIPID-N-0344 | PE(22:1_18:0) | GP | PE | 1.3627 | 0.0037 | | 0.0193 | | 1.9164 | | 0.9384 | | |  |
| LIPID-N-1262 | PS(19:0_16:1) | GP | PS | 1.3098 | 0.0037 | | 0.0193 | | 1.8938 | | 0.9213 | | |  |
| LIPID-P-0036 | Carnitine C12:1-OH | FA | CAR | 1.1507 | 0.0239 | | 0.0708 | | 1.8912 | | 0.9193 | | |  |
| LIPID-P-2252 | HexCer(d19:1/24:0) | SP | HexCer-NS | 1.0958 | 0.0201 | | 0.0631 | | 1.8885 | | 0.9172 | | |  |
| LIPID-P-1297 | Carnitine C22:2 | FA | CAR | 1.4402 | 0.0013 | | 0.0104 | | 1.8834 | | 0.9134 | | |  |
| LIPID-N-0214 | PC(14:0_16:1) | GP | PC | 1.1819 | 0.0289 | | 0.0788 | | 1.8793 | | 0.9102 | | |  |
| LIPID-P-0406 | LPC(O-20:3) | GP | LPC-O | 1.2752 | 0.0065 | | 0.0276 | | 1.8740 | | 0.9061 | | |  |
| LIPID-N-1133 | PG(16:0_15:1) | GP | PG | 1.6802 | 0.0000 | | 0.0040 | | 1.8496 | | 0.8872 | | |  |
| LIPID-P-0021 | Carnitine C16:0 | FA | CAR | 1.5964 | 0.0002 | | 0.0057 | | 1.8408 | | 0.8803 | | |  |
| LIPID-N-0862 | PE(O-18:3_22:4) | GP | PE-O | 1.3998 | 0.0022 | | 0.0133 | | 1.8406 | | 0.8802 | | |  |
| LIPID-N-0343 | PE(22:0_18:1) | GP | PE | 1.2603 | 0.0093 | | 0.0358 | | 1.8330 | | 0.8742 | | |  |
| LIPID-N-1252 | PS(18:0_24:0) | GP | PS | 1.4298 | 0.0014 | | 0.0104 | | 1.8329 | | 0.8742 | | |  |
| LIPID-P-0569 | PC(O-18:1_22:6) | GP | PC-O | 1.2777 | 0.0401 | | 0.0994 | | 1.8053 | | 0.8522 | | |  |
| LIPID-N-0730 | PE(O-18:0_16:0) | GP | PE-O | 1.5499 | 0.0008 | | 0.0082 | | 1.7994 | | 0.8475 | | |  |
| LIPID-N-0228 | PC(16:1_16:1) | GP | PC | 1.3026 | 0.0125 | | 0.0450 | | 1.7815 | | 0.8331 | | |  |
| LIPID-P-0736 | SM(d18:1/14:0) | SP | SM | 1.2583 | 0.0071 | | 0.0297 | | 1.7616 | | 0.8169 | | |  |
| LIPID-N-0854 | PE(O-15:1_22:6) | GP | PE-O | 1.1862 | 0.0059 | | 0.0257 | | 1.7556 | | 0.8119 | | |  |
| LIPID-N-1019 | PC(15:1_18:1) | GP | PC | 1.1650 | 0.0059 | | 0.0257 | | 1.7531 | | 0.8099 | | |  |
| LIPID-N-0570 | PS(18:0_18:1) | GP | PS | 1.5301 | 0.0006 | | 0.0070 | | 1.7458 | | 0.8039 | | |  |
| LIPID-N-0562 | PS(17:0_17:0) | GP | PS | 1.1545 | 0.0208 | | 0.0638 | | 1.7403 | | 0.7993 | | |  |
| LIPID-P-0094 | SPH(d18:0) | SP | SPH | 1.3502 | 0.0076 | | 0.0311 | | 1.7385 | | 0.7979 | | |  |
| LIPID-N-0820 | PE(O-18:1_22:4) | GP | PE-O | 1.1204 | 0.0205 | | 0.0631 | | 1.7339 | | 0.7940 | | |  |
| LIPID-N-1283 | PS(21:0_24:1) | GP | PS | 1.4714 | 0.0021 | | 0.0133 | | 1.7325 | | 0.7928 | | |  |
| LIPID-N-0316 | PC(20:3_20:4) | GP | PC | 1.4341 | 0.0006 | | 0.0070 | | 1.7260 | | 0.7874 | | |  |
| LIPID-N-1231 | PS(14:0_19:0) | GP | PS | 1.2174 | 0.0059 | | 0.0257 | | 1.7205 | | 0.7828 | | |  |
| LIPID-N-0595 | PS(18:0_22:5) | GP | PS | 1.3360 | 0.0055 | | 0.0253 | | 1.7095 | | 0.7736 | | |  |
| LIPID-N-1083 | PE(24:0_20:4) | GP | PE | 1.0606 | 0.0431 | | 0.1039 | | 1.7061 | | 0.7707 | | |  |
| LIPID-P-0638 | PE(P-16:0_22:1) | GP | PE-P | 1.0250 | 0.0289 | | 0.0788 | | 1.7023 | | 0.7675 | | |  |
| LIPID-P-1302 | Carnitine C20:4 | FA | CAR | 1.1846 | 0.0093 | | 0.0358 | | 1.7023 | | 0.7675 | | |  |
| LIPID-N-1028 | PC(14:1_20:4) | GP | PC | 1.4363 | 0.0043 | | 0.0207 | | 1.6989 | | 0.7646 | | |  |
| LIPID-N-0201 | LPS(22:6) | GP | LPS | 1.2430 | 0.0119 | | 0.0434 | | 1.6874 | | 0.7548 | | |  |
| LIPID-N-0783 | PE(O-18:1_22:2) | GP | PE-O | 1.0396 | 0.0205 | | 0.0631 | | 1.6819 | | 0.7501 | | |  |
| LIPID-N-0731 | PE(O-20:0_16:0) | GP | PE-O | 1.2776 | 0.0076 | | 0.0312 | | 1.6721 | | 0.7417 | | |  |
| LIPID-N-0203 | PC(16:0_14:0) | GP | PC | 1.2162 | 0.0110 | | 0.0415 | | 1.6717 | | 0.7413 | | |  |
| LIPID-P-1315 | PE(P-14:0_18:1) | GP | PE-P | 1.0692 | 0.0205 | | 0.0631 | | 1.6595 | | 0.7307 | | |  |
| LIPID-P-0024 | Carnitine C20:0 | FA | CAR | 1.2188 | 0.0119 | | 0.0434 | | 1.6566 | | 0.7282 | | |  |
| LIPID-N-0738 | PE(O-18:0_18:1) | GP | PE-O | 1.1106 | 0.0362 | | 0.0935 | | 1.6559 | | 0.7276 | | |  |
| LIPID-P-0734 | SM(d18:0/22:0) | SP | SM | 1.2587 | 0.0137 | | 0.0483 | | 1.6529 | | 0.7250 | | |  |
| LIPID-N-0192 | LPS(18:1) | GP | LPS | 1.3937 | 0.0022 | | 0.0133 | | 1.6501 | | 0.7225 | | |  |
| LIPID-P-0023 | Carnitine C18:0 | FA | CAR | 1.3792 | 0.0029 | | 0.0163 | | 1.6453 | | 0.7183 | | |  |
| LIPID-N-1132 | PG(15:0_16:1) | GP | PG | 1.3868 | 0.0006 | | 0.0070 | | 1.6437 | | 0.7170 | | |  |
| LIPID-P-1578 | BMP(22:6_22:6) | GP | BMP | 1.0389 | 0.0275 | | 0.0778 | | 1.6264 | | 0.7017 | | |  |
| LIPID-P-0537 | PC(O-16:0_16:1) | GP | PC-O | 1.2941 | 0.0048 | | 0.0226 | | 1.6185 | | 0.6947 | | |  |
| LIPID-N-0243 | PC(18:1_24:1) | GP | PC | 1.3764 | 0.0003 | | 0.0057 | | 1.6080 | | 0.6852 | | |  |
| LIPID-N-0389 | PE(16:0_22:4) | GP | PE | 1.3925 | 0.0022 | | 0.0133 | | 1.6058 | | 0.6833 | | |  |
| LIPID-P-0048 | Carnitine C16:2 | FA | CAR | 1.0444 | 0.0059 | | 0.0257 | | 1.5922 | | 0.6710 | | |  |
| LIPID-P-0388 | LPC(22:4/0:0) | GP | LPC | 1.4449 | 0.0017 | | 0.0124 | | 1.5737 | | 0.6541 | | |  |
| LIPID-N-1239 | PS(15:0_21:0) | GP | PS | 1.2095 | 0.0149 | | 0.0509 | | 1.5538 | | 0.6358 | | |  |
| LIPID-P-0364 | LPC(24:0) | GP | LPC | 1.5070 | 0.0018 | | 0.0126 | | 1.5523 | | 0.6344 | | |  |
| LIPID-N-0462 | PG(18:0_22:6) | GP | PG | 1.0299 | 0.0391 | | 0.0994 | | 1.5434 | | 0.6261 | | |  |
| LIPID-N-0403 | PE(18:0_22:5) | GP | PE | 1.1767 | 0.0059 | | 0.0257 | | 1.5431 | | 0.6258 | | |  |
| LIPID-P-0351 | LPC(0:0/15:0) | GP | LPC | 1.4740 | 0.0021 | | 0.0133 | | 1.5412 | | 0.6241 | | |  |
| LIPID-N-0599 | PS(18:1_22:6) | GP | PS | 1.2973 | 0.0063 | | 0.0267 | | 1.5405 | | 0.6234 | | |  |
| LIPID-N-0197 | LPS(20:3) | GP | LPS | 1.3460 | 0.0061 | | 0.0263 | | 1.5360 | | 0.6191 | | |  |
| LIPID-P-0761 | SM(d18:2/18:1) | SP | SM | 1.0175 | 0.0259 | | 0.0750 | | 1.5258 | | 0.6095 | | |  |
| LIPID-P-0049 | Carnitine C18:2 | FA | CAR | 1.0929 | 0.0006 | | 0.0070 | | 1.5217 | | 0.6057 | | |  |
| LIPID-P-0387 | LPC(0:0/22:4) | GP | LPC | 1.4588 | 0.0015 | | 0.0115 | | 1.5149 | | 0.5993 | | |  |
| LIPID-P-0765 | SM(d18:2/24:1) | SP | SM | 1.4994 | 0.0004 | | 0.0064 | | 1.5109 | | 0.5954 | | |  |
| LIPID-N-0035 | (±)15-HETE | FA | Eicosanoid | 1.1058 | 0.0240 | | 0.0708 | | 1.5076 | | 0.5923 | | |  |
| LIPID-N-1079 | PE(15:0_22:4) | GP | PE | 1.0737 | 0.0233 | | 0.0694 | | 1.4987 | | 0.5837 | | |  |
| LIPID-N-0741 | PE(O-20:0_18:1) | GP | PE-O | 1.4590 | 0.0009 | | 0.0087 | | 1.4972 | | 0.5822 | | |  |
| LIPID-N-0326 | PE(17:0_17:0) | GP | PE | 1.0236 | 0.0425 | | 0.1034 | | 1.4965 | | 0.5816 | | |  |
| LIPID-P-0402 | LPC(O-22:1) | GP | LPC-O | 1.0232 | 0.0458 | | 0.1084 | | 1.4860 | | 0.5715 | | |  |
| LIPID-N-1153 | PG(15:0_18:2) | GP | PG | 1.2869 | 0.0026 | | 0.0154 | | 1.4784 | | 0.5640 | | |  |
| LIPID-P-1567 | SM(d18:2/24:2) | SP | SM | 1.3724 | 0.0037 | | 0.0193 | | 1.4777 | | 0.5633 | | |  |
| LIPID-N-0439 | PG(17:0_18:1) | GP | PG | 1.5034 | 0.0003 | | 0.0057 | | 1.4767 | | 0.5624 | | |  |
| LIPID-N-0906 | PC(15:0_18:1) | GP | PC | 1.3186 | 0.0012 | | 0.0099 | | 1.4753 | | 0.5610 | | |  |
| LIPID-N-1117 | PG(15:0_22:0) | GP | PG | 1.0739 | 0.0276 | | 0.0778 | | 1.4621 | | 0.5481 | | |  |
| LIPID-N-0609 | CerP(d18:1/18:1) | SP | CerP | 1.2448 | 0.0042 | | 0.0207 | | 1.4564 | | 0.5424 | | |  |
| LIPID-N-0362 | PE(18:1_22:1) | GP | PE | 1.2461 | 0.0115 | | 0.0426 | | 1.4474 | | 0.5335 | | |  |
| LIPID-P-0373 | LPC(0:0/20:1) | GP | LPC | 1.2239 | 0.0190 | | 0.0615 | | 1.4298 | | 0.5158 | | |  |
| LIPID-N-0359 | PE(22:2_18:0) | GP | PE | 1.0611 | 0.0401 | | 0.0994 | | 1.4252 | | 0.5112 | | |  |
| LIPID-N-0276 | PC(22:4_16:0) | GP | PC | 1.2690 | 0.0040 | | 0.0202 | | 1.4204 | | 0.5063 | | |  |
| LIPID-P-0363 | LPC(22:0) | GP | LPC | 1.2632 | 0.0168 | | 0.0557 | | 1.4111 | | 0.4969 | | |  |
| LIPID-N-0468 | PG(20:3_22:6) | GP | PG | 1.2809 | 0.0108 | | 0.0410 | | 1.3962 | | 0.4815 | | |  |
| LIPID-N-0459 | PG(18:1_20:4) | GP | PG | 1.2045 | 0.0245 | | 0.0721 | | 1.3922 | | 0.4774 | | |  |
| LIPID-N-0995 | PC(14:0_17:0) | GP | PC | 1.0537 | 0.0430 | | 0.1039 | | 1.3921 | | 0.4773 | | |  |
| LIPID-N-0764 | PE(O-22:0_18:2) | GP | PE-O | 1.2246 | 0.0158 | | 0.0534 | | 1.3854 | | 0.4703 | | |  |
| LIPID-P-0737 | SM(d18:1/15:0) | SP | SM | 1.3819 | 0.0030 | | 0.0169 | | 1.3847 | | 0.4696 | | |  |
| LIPID-P-0753 | SM(d18:1/19:1) | SP | SM | 1.1222 | 0.0114 | | 0.0425 | | 1.3782 | | 0.4628 | | |  |
| LIPID-P-0357 | LPC(0:0/18:0) | GP | LPC | 1.1113 | 0.0276 | | 0.0779 | | 1.3755 | | 0.4600 | | |  |
| LIPID-N-1130 | PG(14:0_16:1) | GP | PG | 1.2229 | 0.0107 | | 0.0407 | | 1.3691 | | 0.4533 | | |  |
| LIPID-P-0747 | SM(d18:1/25:0) | SP | SM | 1.1605 | 0.0225 | | 0.0683 | | 1.3551 | | 0.4384 | | |  |
| LIPID-N-1080 | PE(15:1_22:3) | GP | PE | 1.2628 | 0.0140 | | 0.0483 | | 1.3498 | | 0.4328 | | |  |
| LIPID-N-1086 | PE(15:1_22:4) | GP | PE | 1.2489 | 0.0401 | | 0.0994 | | 1.3331 | | 0.4147 | | |  |
| LIPID-P-1929 | LPC(16:2) | GP | LPC | 1.4463 | 0.0039 | | 0.0198 | | 1.3221 | | 0.4029 | | |  |
| LIPID-N-0172 | LPG(20:4) | GP | LPG | 1.1142 | 0.0379 | | 0.0973 | | 1.3220 | | 0.4027 | | |  |
| LIPID-N-1433 | PI(16:0_22:4) | GP | PI | 1.1538 | 0.0275 | | 0.0778 | | 1.3104 | | 0.3900 | | |  |
| LIPID-N-0216 | PC(16:0_16:1) | GP | PC | 1.1704 | 0.0303 | | 0.0814 | | 1.3083 | | 0.3877 | | |  |
| LIPID-N-0340 | PE(20:0_18:1) | GP | PE | 1.1195 | 0.0319 | | 0.0841 | | 1.2890 | | 0.3662 | | |  |
| LIPID-N-1077 | PE(14:0_22:4) | GP | PE | 1.0716 | 0.0463 | | 0.1094 | | 1.2744 | | 0.3498 | | |  |
| LIPID-P-0738 | SM(d18:1/16:0) | SP | SM | 1.5618 | 0.0003 | | 0.0057 | | 1.2660 | | 0.3402 | | |  |
| LIPID-N-0217 | PC(14:0_18:1) | GP | PC | 1.3071 | 0.0098 | | 0.0375 | | 1.2014 | | 0.2647 | | |  |
| LIPID-N-0206 | PC(16:0_16:0) | GP | PC | 1.1740 | 0.0308 | | 0.0823 | | 1.1584 | | 0.2122 | | |  |
| LIPID-N-0336 | PE(17:0_18:1) | GP | PE | 1.0017 | 0.0283 | | 0.0788 | | 0.8618 | | -0.2146 | | |  |
| LIPID-P-0360 | LPC(19:0/0:0) | GP | LPC | 1.3932 | 0.0012 | | 0.0099 | | 0.8544 | | -0.2269 | | |  |
| LIPID-N-0413 | PE(22:6_16:0) | GP | PE | 1.1773 | 0.0208 | | 0.0637 | | 0.8192 | | -0.2877 | | |  |
| LIPID-P-0028 | Carnitine C5:1 | FA | CAR | 1.1621 | 0.0169 | | 0.0558 | | 0.8104 | | -0.3032 | | |  |
| LIPID-N-1179 | PG(18:1_21:2) | GP | PG | 1.1887 | 0.0240 | | 0.0708 | | 0.8090 | | -0.3059 | | |  |
| LIPID-N-1201 | PG(24:0_20:4) | GP | PG | 1.1857 | 0.0132 | | 0.0474 | | 0.8084 | | -0.3069 | | |  |
| LIPID-N-0098 | FFA(20:4) | FA | FFA | 1.0958 | 0.0228 | | 0.0686 | | 0.8069 | | -0.3096 | | |  |
| LIPID-N-1096 | PE(20:2_20:4) | GP | PE | 1.1964 | 0.0205 | | 0.0631 | | 0.8038 | | -0.3152 | | |  |
| LIPID-N-0814 | PE(O-18:1_20:4) | GP | PE-O | 1.3994 | 0.0014 | | 0.0106 | | 0.8034 | | -0.3159 | | |  |
| LIPID-N-1212 | PG(19:2_20:4) | GP | PG | 1.0834 | 0.0284 | | 0.0788 | | 0.8015 | | -0.3192 | | |  |
| LIPID-P-0358 | LPC(18:0/0:0) | GP | LPC | 1.2687 | 0.0113 | | 0.0425 | | 0.7980 | | -0.3256 | | |  |
| LIPID-N-0794 | PE(O-18:1_20:3) | GP | PE-O | 1.3609 | 0.0018 | | 0.0126 | | 0.7927 | | -0.3352 | | |  |
| LIPID-N-0438 | PG(16:0_18:1) | GP | PG | 1.3052 | 0.0060 | | 0.0258 | | 0.7925 | | -0.3354 | | |  |
| LIPID-N-1122 | PG(18:0_22:0) | GP | PG | 1.5011 | 0.0024 | | 0.0142 | | 0.7861 | | -0.3473 | | |  |
| LIPID-N-0170 | LPG(18:1) | GP | LPG | 1.2352 | 0.0120 | | 0.0435 | | 0.7834 | | -0.3522 | | |  |
| LIPID-N-0734 | PE(O-16:1_16:0) | GP | PE-O | 1.3252 | 0.0062 | | 0.0265 | | 0.7828 | | -0.3533 | | |  |
| LIPID-N-1155 | PG(16:0_17:2) | GP | PG | 1.1563 | 0.0163 | | 0.0547 | | 0.7824 | | -0.3540 | | |  |
| LIPID-N-1000 | PC(15:1_16:1) | GP | PC | 1.1148 | 0.0232 | | 0.0693 | | 0.7769 | | -0.3641 | | |  |
| LIPID-N-1242 | PS(16:0_21:0) | GP | PS | 1.1572 | 0.0188 | | 0.0611 | | 0.7763 | | -0.3654 | | |  |
| LIPID-P-0380 | LPC(0:0/20:2) | GP | LPC | 1.1956 | 0.0165 | | 0.0552 | | 0.7694 | | -0.3782 | | |  |
| LIPID-N-0171 | LPG(18:2) | GP | LPG | 1.3759 | 0.0020 | | 0.0133 | | 0.7645 | | -0.3875 | | |  |
| LIPID-P-0372 | LPC(19:1) | GP | LPC | 1.0025 | 0.0487 | | 0.1130 | | 0.7591 | | -0.3976 | | |  |
| LIPID-N-0455 | PG(18:0_20:4) | GP | PG | 1.1338 | 0.0174 | | 0.0571 | | 0.7568 | | -0.4020 | | |  |
| LIPID-P-0160 | CoenzymeQ9 | PR | CoQ | 1.1265 | 0.0275 | | 0.0778 | | 0.7563 | | -0.4029 | | |  |
| LIPID-N-1161 | PG(16:0_22:2) | GP | PG | 1.2044 | 0.0067 | | 0.0282 | | 0.7559 | | -0.4037 | | |  |
| LIPID-N-0606 | CerP(d18:1/24:0) | SP | CerP | 1.2764 | 0.0124 | | 0.0449 | | 0.7542 | | -0.4070 | | |  |
| LIPID-N-1200 | PG(22:0_20:4) | GP | PG | 1.1564 | 0.0260 | | 0.0752 | | 0.7536 | | -0.4080 | | |  |
| LIPID-N-0758 | PE(O-18:1_20:1) | GP | PE-O | 1.0624 | 0.0259 | | 0.0750 | | 0.7534 | | -0.4085 | | |  |
| LIPID-N-0773 | PE(O-18:1_18:2) | GP | PE-O | 1.1779 | 0.0075 | | 0.0307 | | 0.7516 | | -0.4120 | | |  |
| LIPID-N-0810 | PE(O-20:5_16:0) | GP | PE-O | 1.0217 | 0.0269 | | 0.0769 | | 0.7516 | | -0.4120 | | |  |
| LIPID-N-0303 | PC(16:0_22:6) | GP | PC | 1.2247 | 0.0272 | | 0.0775 | | 0.7503 | | -0.4144 | | |  |
| LIPID-N-0426 | PE(22:6_18:1) | GP | PE | 1.1423 | 0.0291 | | 0.0788 | | 0.7489 | | -0.4172 | | |  |
| LIPID-P-0392 | LPC(22:6) | GP | LPC | 1.6360 | 0.0001 | | 0.0040 | | 0.7473 | | -0.4202 | | |  |
| LIPID-N-1114 | PG(17:0_18:0) | GP | PG | 1.3755 | 0.0029 | | 0.0164 | | 0.7473 | | -0.4203 | | |  |
| LIPID-N-0260 | PC(20:2_18:1) | GP | PC | 1.3074 | 0.0186 | | 0.0604 | | 0.7460 | | -0.4228 | | |  |
| LIPID-N-0760 | PE(O-20:1_18:1) | GP | PE-O | 1.0493 | 0.0396 | | 0.0994 | | 0.7458 | | -0.4231 | | |  |
| LIPID-N-1162 | PG(18:0_20:2) | GP | PG | 1.0768 | 0.0470 | | 0.1100 | | 0.7438 | | -0.4270 | | |  |
| LIPID-N-1321 | PS(18:2_18:2) | GP | PS | 1.1562 | 0.0197 | | 0.0631 | | 0.7378 | | -0.4387 | | |  |
| LIPID-N-1380 | PI(16:0_19:0) | GP | PI | 1.3872 | 0.0093 | | 0.0358 | | 0.7376 | | -0.4391 | | |  |
| LIPID-P-0756 | SM(d18:1/22:1) | SP | SM | 1.1124 | 0.0269 | | 0.0769 | | 0.7355 | | -0.4432 | | |  |
| LIPID-P-0419 | LPE(0:0/20:1) | GP | LPE | 1.0090 | 0.0253 | | 0.0741 | | 0.7326 | | -0.4489 | | |  |
| LIPID-N-0218 | PC(16:0_18:1) | GP | PC | 1.3789 | 0.0059 | | 0.0257 | | 0.7301 | | -0.4539 | | |  |
| LIPID-P-0409 | LPE(17:0) | GP | LPE | 1.1130 | 0.0164 | | 0.0549 | | 0.7299 | | -0.4542 | | |  |
| LIPID-N-1315 | PS(21:0_18:3) | GP | PS | 1.0812 | 0.0339 | | 0.0885 | | 0.7263 | | -0.4614 | | |  |
| LIPID-N-0422 | PE(16:1_22:6) | GP | PE | 1.1573 | 0.0228 | | 0.0686 | | 0.7257 | | -0.4626 | | |  |
| LIPID-N-1059 | PE(15:0_18:2) | GP | PE | 1.2804 | 0.0039 | | 0.0198 | | 0.7252 | | -0.4635 | | |  |
| LIPID-N-1070 | PE(18:0_18:3) | GP | PE | 1.1023 | 0.0204 | | 0.0631 | | 0.7239 | | -0.4661 | | |  |
| LIPID-N-1185 | PG(22:0_20:3) | GP | PG | 1.1527 | 0.0148 | | 0.0508 | | 0.7236 | | -0.4667 | | |  |
| LIPID-N-1398 | PI(19:0_17:1) | GP | PI | 1.0062 | 0.0210 | | 0.0640 | | 0.7233 | | -0.4674 | | |  |
| LIPID-N-1145 | PG(19:0_20:1) | GP | PG | 1.2860 | 0.0066 | | 0.0278 | | 0.7212 | | -0.4716 | | |  |
| LIPID-N-1296 | PS(19:0_18:2) | GP | PS | 1.0256 | 0.0500 | | 0.1150 | | 0.7189 | | -0.4761 | | |  |
| LIPID-P-0664 | PE(P-18:1_22:6) | GP | PE-P | 1.2110 | 0.0227 | | 0.0686 | | 0.7170 | | -0.4799 | | |  |
| LIPID-N-1422 | PI(14:1_24:2) | GP | PI | 1.0754 | 0.0269 | | 0.0769 | | 0.7149 | | -0.4843 | | |  |
| LIPID-N-0522 | PI(16:0_20:4) | GP | PI | 1.0093 | 0.0274 | | 0.0778 | | 0.7130 | | -0.4881 | | |  |
| LIPID-N-0238 | PC(18:1_19:1) | GP | PC | 1.3335 | 0.0119 | | 0.0434 | | 0.7109 | | -0.4923 | | |  |
| LIPID-N-0569 | PS(18:0_17:1) | GP | PS | 1.3694 | 0.0036 | | 0.0193 | | 0.7107 | | -0.4927 | | |  |
| LIPID-N-0375 | PE(18:2_22:1) | GP | PE | 1.0989 | 0.0158 | | 0.0534 | | 0.7097 | | -0.4946 | | |  |
| LIPID-N-1301 | PS(21:0_18:2) | GP | PS | 1.1875 | 0.0408 | | 0.1010 | | 0.7083 | | -0.4977 | | |  |
| LIPID-N-0440 | PG(18:0_18:1) | GP | PG | 1.2639 | 0.0056 | | 0.0257 | | 0.7075 | | -0.4991 | | |  |
| LIPID-N-0383 | PE(16:0_20:4) | GP | PE | 1.3185 | 0.0053 | | 0.0243 | | 0.7066 | | -0.5011 | | |  |
| LIPID-N-1120 | PG(16:0_22:0) | GP | PG | 1.6120 | 0.0003 | | 0.0057 | | 0.7027 | | -0.5089 | | |  |
| LIPID-N-1307 | PS(16:0_20:3) | GP | PS | 1.1089 | 0.0294 | | 0.0791 | | 0.7007 | | -0.5131 | | |  |
| LIPID-P-0096 | SPH(d18:1) | SP | SPH | 1.3775 | 0.0012 | | 0.0099 | | 0.6988 | | -0.5171 | | |  |
| LIPID-N-1195 | PG(18:2_22:2) | GP | PG | 1.0537 | 0.0263 | | 0.0756 | | 0.6936 | | -0.5278 | | |  |
| LIPID-N-0873 | PE(O-20:2_22:6) | GP | PE-O | 1.4691 | 0.0140 | | 0.0483 | | 0.6931 | | -0.5289 | | |  |
| LIPID-N-0994 | PC(13:0_18:0) | GP | PC | 1.0144 | 0.0232 | | 0.0693 | | 0.6884 | | -0.5388 | | |  |
| LIPID-P-0390 | LPC(0:0/22:5) | GP | LPC | 1.6666 | 0.0000 | | 0.0036 | | 0.6878 | | -0.5400 | | |  |
| LIPID-N-0196 | LPS(18:2) | GP | LPS | 1.0058 | 0.0380 | | 0.0974 | | 0.6842 | | -0.5475 | | |  |
| LIPID-N-1338 | PS(18:1_24:4) | GP | PS | 1.4329 | 0.0021 | | 0.0133 | | 0.6837 | | -0.5486 | | |  |
| LIPID-N-0361 | PE(20:1_20:1) | GP | PE | 1.1950 | 0.0148 | | 0.0508 | | 0.6833 | | -0.5493 | | |  |
| LIPID-N-0577 | PS(18:0_18:2) | GP | PS | 1.0922 | 0.0252 | | 0.0737 | | 0.6830 | | -0.5500 | | |  |
| LIPID-N-0543 | PI(20:4_20:1) | GP | PI | 1.1924 | 0.0115 | | 0.0426 | | 0.6819 | | -0.5524 | | |  |
| LIPID-N-1183 | PG(22:1_18:2) | GP | PG | 1.5326 | 0.0004 | | 0.0061 | | 0.6787 | | -0.5592 | | |  |
| LIPID-N-0235 | PC(18:1_18:1) | GP | PC | 1.5262 | 0.0010 | | 0.0090 | | 0.6781 | | -0.5603 | | |  |
| LIPID-N-0175 | LPI(16:0) | GP | LPI | 1.0204 | 0.0395 | | 0.0994 | | 0.6765 | | -0.5639 | | |  |
| LIPID-N-0339 | PE(20:1_18:0) | GP | PE | 1.5191 | 0.0011 | | 0.0098 | | 0.6760 | | -0.5650 | | |  |
| LIPID-N-0809 | PE(O-16:1_20:4) | GP | PE-O | 1.6263 | 0.0001 | | 0.0054 | | 0.6744 | | -0.5684 | | |  |
| LIPID-N-1322 | PS(18:1_20:3) | GP | PS | 1.2170 | 0.0139 | | 0.0483 | | 0.6737 | | -0.5697 | | |  |
| LIPID-N-1277 | PS(21:0_18:1) | GP | PS | 1.4376 | 0.0027 | | 0.0156 | | 0.6733 | | -0.5707 | | |  |
| LIPID-N-1194 | PG(18:1_22:3) | GP | PG | 1.2466 | 0.0157 | | 0.0534 | | 0.6727 | | -0.5719 | | |  |
| LIPID-N-1295 | PS(18:0_19:2) | GP | PS | 1.1867 | 0.0184 | | 0.0599 | | 0.6715 | | -0.5745 | | |  |
| LIPID-N-0544 | PI(18:0_22:5) | GP | PI | 1.0338 | 0.0203 | | 0.0631 | | 0.6710 | | -0.5757 | | |  |
| LIPID-N-0334 | PE(16:0_18:1) | GP | PE | 1.4341 | 0.0008 | | 0.0082 | | 0.6706 | | -0.5764 | | |  |
| LIPID-N-0524 | PI(18:0_20:4) | GP | PI | 1.2780 | 0.0053 | | 0.0243 | | 0.6700 | | -0.5777 | | |  |
| LIPID-N-0959 | LNAPE(16:0/N-18:1) | GP | LNAPE | 1.3143 | 0.0029 | | 0.0163 | | 0.6698 | | -0.5782 | | |  |
| LIPID-N-1102 | PE(22:3_20:4) | GP | PE | 1.2862 | 0.0140 | | 0.0483 | | 0.6684 | | -0.5811 | | |  |
| LIPID-N-1376 | PI(16:0_16:0) | GP | PI | 1.0300 | 0.0228 | | 0.0686 | | 0.6669 | | -0.5844 | | |  |
| LIPID-N-0482 | PI(18:0_18:1) | GP | PI | 1.3437 | 0.0021 | | 0.0133 | | 0.6653 | | -0.5879 | | |  |
| LIPID-N-1381 | PI(17:0_18:0) | GP | PI | 1.2564 | 0.0113 | | 0.0425 | | 0.6588 | | -0.6020 | | |  |
| LIPID-P-1399 | PE(P-20:3_20:4) | GP | PE-P | 1.1969 | 0.0149 | | 0.0509 | | 0.6577 | | -0.6045 | | |  |
| LIPID-N-1397 | PI(15:0_21:1) | GP | PI | 1.4201 | 0.0011 | | 0.0095 | | 0.6572 | | -0.6056 | | |  |
| LIPID-P-1373 | PE(P-16:1_20:4) | GP | PE-P | 1.1685 | 0.0332 | | 0.0870 | | 0.6571 | | -0.6057 | | |  |
| LIPID-P-0412 | LPE(20:0) | GP | LPE | 1.0579 | 0.0166 | | 0.0555 | | 0.6568 | | -0.6065 | | |  |
| LIPID-N-0166 | LPG(16:0) | GP | LPG | 1.2617 | 0.0078 | | 0.0317 | | 0.6528 | | -0.6154 | | |  |
| LIPID-N-0400 | PE(18:0_20:5) | GP | PE | 1.1970 | 0.0079 | | 0.0319 | | 0.6520 | | -0.6171 | | |  |
| LIPID-N-0384 | PE(18:3_18:1) | GP | PE | 1.1377 | 0.0161 | | 0.0543 | | 0.6463 | | -0.6297 | | |  |
| LIPID-N-1423 | PI(18:0_20:3) | GP | PI | 1.3427 | 0.0027 | | 0.0158 | | 0.6461 | | -0.6301 | | |  |
| LIPID-N-0905 | PC(18:1_20:5) | GP | PC | 1.0257 | 0.0288 | | 0.0788 | | 0.6439 | | -0.6351 | | |  |
| LIPID-N-0357 | PE(20:0_18:2) | GP | PE | 1.5121 | 0.0020 | | 0.0133 | | 0.6439 | | -0.6351 | | |  |
| LIPID-P-0354 | LPC(16:0/0:0) | GP | LPC | 1.3220 | 0.0035 | | 0.0191 | | 0.6435 | | -0.6360 | | |  |
| LIPID-N-0755 | PE(O-16:1_20:1) | GP | PE-O | 1.5308 | 0.0008 | | 0.0082 | | 0.6434 | | -0.6362 | | |  |
| LIPID-N-0250 | PC(16:1_18:2) | GP | PC | 1.2220 | 0.0080 | | 0.0323 | | 0.6432 | | -0.6368 | | |  |
| LIPID-P-0652 | PE(P-16:0_20:4) | GP | PE-P | 1.3706 | 0.0049 | | 0.0231 | | 0.6425 | | -0.6383 | | |  |
| LIPID-N-0466 | PG(18:2_22:6) | GP | PG | 1.0245 | 0.0140 | | 0.0483 | | 0.6404 | | -0.6429 | | |  |
| LIPID-N-0777 | PE(O-20:1_18:2) | GP | PE-O | 1.1904 | 0.0172 | | 0.0567 | | 0.6403 | | -0.6432 | | |  |
| LIPID-P-0428 | LPE(18:3) | GP | LPE | 1.0993 | 0.0168 | | 0.0557 | | 0.6401 | | -0.6436 | | |  |
| LIPID-N-0538 | PI(20:4_18:1) | GP | PI | 1.2902 | 0.0027 | | 0.0158 | | 0.6400 | | -0.6438 | | |  |
| LIPID-N-1095 | PE(20:1_20:5) | GP | PE | 1.0569 | 0.0289 | | 0.0788 | | 0.6393 | | -0.6455 | | |  |
| LIPID-N-0190 | LPS(24:0) | GP | LPS | 1.0479 | 0.0369 | | 0.0952 | | 0.6391 | | -0.6458 | | |  |
| LIPID-P-1322 | PE(P-20:0_18:1) | GP | PE-P | 1.2176 | 0.0093 | | 0.0358 | | 0.6390 | | -0.6461 | | |  |
| LIPID-N-0416 | PE(18:1_22:5) | GP | PE | 1.0275 | 0.0246 | | 0.0721 | | 0.6382 | | -0.6479 | | |  |
| LIPID-N-1366 | PS(20:3_20:5) | GP | PS | 1.0206 | 0.0491 | | 0.1135 | | 0.6381 | | -0.6481 | | |  |
| LIPID-N-0502 | PI(18:0_20:2) | GP | PI | 1.4541 | 0.0017 | | 0.0121 | | 0.6377 | | -0.6491 | | |  |
| LIPID-P-0539 | PC(O-18:0_18:1) | GP | PC-O | 1.1917 | 0.0113 | | 0.0425 | | 0.6363 | | -0.6521 | | |  |
| LIPID-N-0352 | PE(18:1_18:1) | GP | PE | 1.5095 | 0.0008 | | 0.0081 | | 0.6360 | | -0.6529 | | |  |
| LIPID-N-1335 | PS(18:1_22:4) | GP | PS | 1.3144 | 0.0035 | | 0.0189 | | 0.6353 | | -0.6545 | | |  |
| LIPID-N-0451 | PG(18:2_18:1) | GP | PG | 1.4674 | 0.0010 | | 0.0089 | | 0.6339 | | -0.6577 | | |  |
| LIPID-P-0003 | Carnitine C3:0 | FA | CAR | 1.3898 | 0.0017 | | 0.0121 | | 0.6338 | | -0.6579 | | |  |
| LIPID-N-0860 | PE(O-20:3_20:4) | GP | PE-O | 1.5485 | 0.0006 | | 0.0070 | | 0.6330 | | -0.6597 | | |  |
| LIPID-N-0176 | LPI(18:0) | GP | LPI | 1.4071 | 0.0027 | | 0.0158 | | 0.6330 | | -0.6598 | | |  |
| LIPID-P-1329 | PE(P-18:1_16:1) | GP | PE-P | 1.2150 | 0.0114 | | 0.0425 | | 0.6323 | | -0.6614 | | |  |
| LIPID-N-1099 | PE(20:2_20:5) | GP | PE | 1.1895 | 0.0057 | | 0.0257 | | 0.6322 | | -0.6616 | | |  |
| LIPID-N-1367 | PS(20:4_20:4) | GP | PS | 1.2037 | 0.0204 | | 0.0631 | | 0.6318 | | -0.6625 | | |  |
| LIPID-N-1441 | PI(18:1_20:4) | GP | PI | 1.4739 | 0.0007 | | 0.0074 | | 0.6311 | | -0.6639 | | |  |
| LIPID-P-0663 | PE(P-20:0_22:6) | GP | PE-P | 1.1461 | 0.0473 | | 0.1102 | | 0.6291 | | -0.6685 | | |  |
| LIPID-N-1011 | PC(15:1_17:1) | GP | PC | 1.1238 | 0.0263 | | 0.0756 | | 0.6263 | | -0.6752 | | |  |
| LIPID-P-2418 | Cer(t17:1/22:1) | SP | Cer-NP | 1.1324 | 0.0400 | | 0.0994 | | 0.6256 | | -0.6768 | | |  |
| LIPID-N-1208 | PG(22:0_20:5) | GP | PG | 1.0811 | 0.0219 | | 0.0666 | | 0.6241 | | -0.6803 | | |  |
| LIPID-N-0685 | LPI(20:1) | GP | LPI | 1.4356 | 0.0017 | | 0.0124 | | 0.6238 | | -0.6807 | | |  |
| LIPID-N-1178 | PG(20:1_18:2) | GP | PG | 1.4906 | 0.0022 | | 0.0133 | | 0.6223 | | -0.6842 | | |  |
| LIPID-N-0430 | PE(22:6_18:2) | GP | PE | 1.4897 | 0.0012 | | 0.0099 | | 0.6199 | | -0.6898 | | |  |
| LIPID-N-0686 | LPI(20:2) | GP | LPI | 1.3790 | 0.0047 | | 0.0225 | | 0.6180 | | -0.6944 | | |  |
| LIPID-N-0968 | LNAPE(20:4/N-18:2) | GP | LNAPE | 1.1493 | 0.0202 | | 0.0631 | | 0.6179 | | -0.6946 | | |  |
| LIPID-N-1081 | PE(18:2_20:2) | GP | PE | 1.5444 | 0.0003 | | 0.0059 | | 0.6152 | | -0.7009 | | |  |
| LIPID-N-1205 | PG(16:0_20:5) | GP | PG | 1.3315 | 0.0036 | | 0.0193 | | 0.6124 | | -0.7075 | | |  |
| LIPID-N-0984 | PC(13:0_16:0) | GP | PC | 1.0384 | 0.0229 | | 0.0687 | | 0.6122 | | -0.7078 | | |  |
| LIPID-N-0356 | PE(20:2_18:0) | GP | PE | 1.5438 | 0.0009 | | 0.0088 | | 0.6118 | | -0.7089 | | |  |
| LIPID-N-1331 | PS(18:1_20:4) | GP | PS | 1.3634 | 0.0046 | | 0.0219 | | 0.6114 | | -0.7098 | | |  |
| LIPID-N-0590 | PS(19:0_20:4) | GP | PS | 1.2844 | 0.0117 | | 0.0432 | | 0.6101 | | -0.7128 | | |  |
| LIPID-N-0358 | PE(20:1_18:1) | GP | PE | 1.6010 | 0.0002 | | 0.0057 | | 0.6098 | | -0.7136 | | |  |
| LIPID-N-0354 | PE(20:2_16:0) | GP | PE | 1.5335 | 0.0006 | | 0.0070 | | 0.6093 | | -0.7149 | | |  |
| LIPID-N-0770 | PE(O-17:1_18:2) | GP | PE-O | 1.3821 | 0.0038 | | 0.0193 | | 0.6086 | | -0.7165 | | |  |
| LIPID-N-1323 | PS(18:2_20:2) | GP | PS | 1.3992 | 0.0033 | | 0.0183 | | 0.6074 | | -0.7193 | | |  |
| LIPID-P-1323 | PE(P-18:0_20:1) | GP | PE-P | 1.1781 | 0.0258 | | 0.0749 | | 0.6016 | | -0.7332 | | |  |
| LIPID-N-1021 | PC(15:1_18:2) | GP | PC | 1.4935 | 0.0019 | | 0.0133 | | 0.6015 | | -0.7333 | | |  |
| LIPID-N-0976 | LNAPE(20:4/N-22:4) | GP | LNAPE | 1.2793 | 0.0042 | | 0.0206 | | 0.6006 | | -0.7356 | | |  |
| LIPID-N-1159 | PG(18:0_19:2) | GP | PG | 1.3684 | 0.0093 | | 0.0358 | | 0.5981 | | -0.7416 | | |  |
| LIPID-P-2419 | Cer(t17:1/22:2) | SP | Cer-NP | 1.2531 | 0.0210 | | 0.0640 | | 0.5979 | | -0.7420 | | |  |
| LIPID-N-1148 | PG(22:0_18:1) | GP | PG | 1.7019 | 0.0001 | | 0.0043 | | 0.5977 | | -0.7424 | | |  |
| LIPID-P-1362 | PE(P-19:0_20:4) | GP | PE-P | 1.1557 | 0.0149 | | 0.0509 | | 0.5956 | | -0.7476 | | |  |
| LIPID-N-1098 | PE(18:2_22:5) | GP | PE | 1.1783 | 0.0079 | | 0.0319 | | 0.5955 | | -0.7479 | | |  |
| LIPID-N-0449 | PG(20:1_18:1) | GP | PG | 1.4735 | 0.0005 | | 0.0070 | | 0.5890 | | -0.7637 | | |  |
| LIPID-N-0494 | PI(17:0_18:2) | GP | PI | 1.2288 | 0.0080 | | 0.0322 | | 0.5890 | | -0.7638 | | |  |
| LIPID-N-1382 | PI(15:0_21:0) | GP | PI | 1.2209 | 0.0059 | | 0.0257 | | 0.5876 | | -0.7672 | | |  |
| LIPID-N-0469 | PI(16:0_18:0) | GP | PI | 1.3742 | 0.0019 | | 0.0129 | | 0.5839 | | -0.7762 | | |  |
| LIPID-N-1063 | PE(17:0_18:2) | GP | PE | 1.3757 | 0.0018 | | 0.0126 | | 0.5832 | | -0.7780 | | |  |
| LIPID-N-0399 | PE(18:1_20:4) | GP | PE | 1.3923 | 0.0038 | | 0.0193 | | 0.5821 | | -0.7807 | | |  |
| LIPID-N-0774 | PE(O-18:2_18:1) | GP | PE-O | 1.5381 | 0.0002 | | 0.0057 | | 0.5821 | | -0.7808 | | |  |
| LIPID-N-1392 | PI(15:0_19:1) | GP | PI | 1.5035 | 0.0003 | | 0.0057 | | 0.5815 | | -0.7821 | | |  |
| LIPID-N-0412 | PE(20:5_18:1) | GP | PE | 1.1550 | 0.0117 | | 0.0432 | | 0.5797 | | -0.7865 | | |  |
| LIPID-N-1169 | PG(20:1_22:1) | GP | PG | 1.5244 | 0.0002 | | 0.0057 | | 0.5791 | | -0.7880 | | |  |
| LIPID-N-1170 | PG(22:0_20:2) | GP | PG | 1.6656 | 0.0004 | | 0.0061 | | 0.5789 | | -0.7886 | | |  |
| LIPID-N-0189 | LPS(22:0) | GP | LPS | 1.1594 | 0.0205 | | 0.0631 | | 0.5725 | | -0.8048 | | |  |
| LIPID-N-0837 | PE(O-18:2_20:4) | GP | PE-O | 1.3909 | 0.0022 | | 0.0133 | | 0.5712 | | -0.8080 | | |  |
| LIPID-N-0366 | PE(16:1_18:2) | GP | PE | 1.1385 | 0.0138 | | 0.0483 | | 0.5700 | | -0.8111 | | |  |
| LIPID-N-0284 | PC(16:0_20:5) | GP | PC | 1.2001 | 0.0078 | | 0.0317 | | 0.5694 | | -0.8125 | | |  |
| LIPID-N-0188 | LPS(20:0) | GP | LPS | 1.3147 | 0.0067 | | 0.0282 | | 0.5686 | | -0.8146 | | |  |
| LIPID-N-0471 | PI(18:0_18:0) | GP | PI | 1.5863 | 0.0001 | | 0.0046 | | 0.5675 | | -0.8172 | | |  |
| LIPID-N-1332 | PS(18:2_20:3) | GP | PS | 1.4809 | 0.0013 | | 0.0101 | | 0.5674 | | -0.8176 | | |  |
| LIPID-N-0615 | LPA(16:0) | GP | LPA | 1.7017 | 0.0001 | | 0.0043 | | 0.5670 | | -0.8185 | | |  |
| LIPID-N-1303 | PS(23:0_18:2) | GP | PS | 1.6321 | 0.0001 | | 0.0046 | | 0.5645 | | -0.8250 | | |  |
| LIPID-P-0378 | LPC(0:0/18:2) | GP | LPC | 1.4453 | 0.0008 | | 0.0079 | | 0.5641 | | -0.8259 | | |  |
| LIPID-N-0581 | PS(18:2_18:1) | GP | PS | 1.3324 | 0.0068 | | 0.0287 | | 0.5617 | | -0.8321 | | |  |
| LIPID-N-0237 | PC(19:0_18:2) | GP | PC | 1.3731 | 0.0019 | | 0.0130 | | 0.5612 | | -0.8335 | | |  |
| LIPID-P-2048 | Cer(d16:1/26:1) | SP | Cer-NS | 1.0802 | 0.0458 | | 0.1084 | | 0.5597 | | -0.8373 | | |  |
| LIPID-P-1333 | PE(P-20:0_18:2) | GP | PE-P | 1.2570 | 0.0171 | | 0.0564 | | 0.5595 | | -0.8377 | | |  |
| LIPID-P-0379 | LPC(18:2/0:0) | GP | LPC | 1.4565 | 0.0006 | | 0.0070 | | 0.5590 | | -0.8390 | | |  |
| LIPID-N-0969 | LNAPE(20:5/N-18:1) | GP | LNAPE | 1.2245 | 0.0074 | | 0.0307 | | 0.5564 | | -0.8458 | | |  |
| LIPID-N-1337 | PS(21:0_20:5) | GP | PS | 1.2075 | 0.0097 | | 0.0374 | | 0.5553 | | -0.8488 | | |  |
| LIPID-N-1311 | PS(19:1_18:2) | GP | PS | 1.5506 | 0.0006 | | 0.0070 | | 0.5544 | | -0.8511 | | |  |
| LIPID-N-0975 | LNAPE(22:6/N-18:2) | GP | LNAPE | 1.4432 | 0.0029 | | 0.0164 | | 0.5531 | | -0.8543 | | |  |
| LIPID-N-0659 | LPA(22:5) | GP | LPA | 1.6548 | 0.0001 | | 0.0043 | | 0.5514 | | -0.8589 | | |  |
| LIPID-P-0438 | LPE(P-16:0) | GP | LPE-P | 1.5027 | 0.0004 | | 0.0064 | | 0.5510 | | -0.8599 | | |  |
| LIPID-N-0996 | PC(13:0_18:1) | GP | PC | 1.2754 | 0.0049 | | 0.0228 | | 0.5493 | | -0.8642 | | |  |
| LIPID-P-0161 | CoenzymeQ10 | PR | CoQ | 1.5687 | 0.0005 | | 0.0066 | | 0.5489 | | -0.8654 | | |  |
| LIPID-N-0844 | PE(O-20:1_20:5) | GP | PE-O | 1.1151 | 0.0137 | | 0.0483 | | 0.5481 | | -0.8676 | | |  |
| LIPID-P-0389 | LPC(20:5) | GP | LPC | 1.1173 | 0.0115 | | 0.0426 | | 0.5472 | | -0.8700 | | |  |
| LIPID-N-1393 | PI(19:0_15:1) | GP | PI | 1.5069 | 0.0003 | | 0.0057 | | 0.5465 | | -0.8718 | | |  |
| LIPID-N-1330 | PS(17:1_20:4) | GP | PS | 1.3343 | 0.0073 | | 0.0302 | | 0.5436 | | -0.8793 | | |  |
| LIPID-N-1072 | PE(18:1_20:2) | GP | PE | 1.6481 | 0.0002 | | 0.0057 | | 0.5431 | | -0.8807 | | |  |
| LIPID-N-1356 | PS(18:2_20:5) | GP | PS | 1.1397 | 0.0118 | | 0.0434 | | 0.5430 | | -0.8810 | | |  |
| LIPID-N-0782 | PE(O-22:1_18:2) | GP | PE-O | 1.3966 | 0.0027 | | 0.0157 | | 0.5429 | | -0.8813 | | |  |
| LIPID-P-0440 | LPE(P-18:0) | GP | LPE-P | 1.4882 | 0.0004 | | 0.0064 | | 0.5425 | | -0.8824 | | |  |
| LIPID-P-0424 | LPE(18:2/0:0) | GP | LPE | 1.3285 | 0.0023 | | 0.0142 | | 0.5405 | | -0.8877 | | |  |
| LIPID-N-1312 | PS(18:1_20:2) | GP | PS | 1.4745 | 0.0012 | | 0.0099 | | 0.5395 | | -0.8904 | | |  |
| LIPID-N-0232 | PC(16:0_18:2) | GP | PC | 1.5662 | 0.0004 | | 0.0061 | | 0.5385 | | -0.8930 | | |  |
| LIPID-N-0368 | PE(18:1_18:2) | GP | PE | 1.4940 | 0.0009 | | 0.0087 | | 0.5379 | | -0.8946 | | |  |
| LIPID-N-0252 | PC(17:1_18:2) | GP | PC | 1.3233 | 0.0047 | | 0.0225 | | 0.5373 | | -0.8961 | | |  |
| LIPID-N-0589 | PS(18:0_20:4) | GP | PS | 1.4997 | 0.0010 | | 0.0093 | | 0.5367 | | -0.8978 | | |  |
| LIPID-P-0097 | SPH(d18:2) | SP | SPH | 1.1575 | 0.0121 | | 0.0438 | | 0.5361 | | -0.8993 | | |  |
| LIPID-N-0892 | PMeOH(18:0_22:4) | GP | PMeOH | 1.1334 | 0.0084 | | 0.0335 | | 0.5359 | | -0.8999 | | |  |
| LIPID-P-0410 | LPE(0:0/18:0) | GP | LPE | 1.4166 | 0.0011 | | 0.0097 | | 0.5359 | | -0.9000 | | |  |
| LIPID-N-1267 | PS(16:0_21:1) | GP | PS | 1.5507 | 0.0005 | | 0.0066 | | 0.5359 | | -0.9001 | | |  |
| LIPID-P-0411 | LPE(18:0/0:0) | GP | LPE | 1.4306 | 0.0010 | | 0.0089 | | 0.5355 | | -0.9010 | | |  |
| LIPID-N-0807 | PE(O-16:0_20:5) | GP | PE-O | 1.3001 | 0.0037 | | 0.0193 | | 0.5346 | | -0.9036 | | |  |
| LIPID-N-1343 | PS(18:3_20:3) | GP | PS | 1.5471 | 0.0003 | | 0.0057 | | 0.5319 | | -0.9107 | | |  |
| LIPID-N-0478 | PI(16:0_18:1) | GP | PI | 1.5759 | 0.0001 | | 0.0046 | | 0.5288 | | -0.9193 | | |  |
| LIPID-N-0287 | PC(18:2_18:3) | GP | PC | 1.3160 | 0.0069 | | 0.0290 | | 0.5271 | | -0.9237 | | |  |
| LIPID-N-0855 | PE(O-18:3_20:4) | GP | PE-O | 1.3098 | 0.0039 | | 0.0196 | | 0.5261 | | -0.9266 | | |  |
| LIPID-N-1287 | PS(16:0_17:2) | GP | PS | 1.5585 | 0.0004 | | 0.0064 | | 0.5248 | | -0.9302 | | |  |
| LIPID-N-0790 | PE(O-18:3_18:1) | GP | PE-O | 1.4428 | 0.0013 | | 0.0101 | | 0.5236 | | -0.9334 | | |  |
| LIPID-P-0423 | LPE(0:0/18:2) | GP | LPE | 1.3626 | 0.0015 | | 0.0115 | | 0.5217 | | -0.9388 | | |  |
| LIPID-N-0292 | PC(20:5_18:0) | GP | PC | 1.3778 | 0.0016 | | 0.0120 | | 0.5206 | | -0.9416 | | |  |
| LIPID-N-0187 | LPS(18:0) | GP | LPS | 1.4535 | 0.0014 | | 0.0109 | | 0.5198 | | -0.9441 | | |  |
| LIPID-N-1379 | PI(15:0_19:0) | GP | PI | 1.4357 | 0.0010 | | 0.0089 | | 0.5173 | | -0.9509 | | |  |
| LIPID-P-1331 | PE(P-18:0_18:2) | GP | PE-P | 1.3983 | 0.0021 | | 0.0133 | | 0.5160 | | -0.9545 | | |  |
| LIPID-P-0559 | PC(O-16:1_20:4) | GP | PC-O | 1.5262 | 0.0005 | | 0.0070 | | 0.5152 | | -0.9568 | | |  |
| LIPID-N-1068 | PE(18:1_17:2) | GP | PE | 1.4789 | 0.0007 | | 0.0076 | | 0.5146 | | -0.9584 | | |  |
| LIPID-N-0350 | PE(18:2_16:0) | GP | PE | 1.4748 | 0.0008 | | 0.0081 | | 0.5139 | | -0.9603 | | |  |
| LIPID-N-1087 | PE(17:2_20:3) | GP | PE | 1.5426 | 0.0008 | | 0.0080 | | 0.5121 | | -0.9655 | | |  |
| LIPID-N-1090 | PE(20:2_18:3) | GP | PE | 1.3409 | 0.0025 | | 0.0147 | | 0.5116 | | -0.9668 | | |  |
| LIPID-N-0178 | LPI(18:1) | GP | LPI | 1.4347 | 0.0025 | | 0.0149 | | 0.5098 | | -0.9719 | | |  |
| LIPID-N-0836 | PE(O-17:2_20:4) | GP | PE-O | 1.4928 | 0.0005 | | 0.0070 | | 0.5084 | | -0.9760 | | |  |
| LIPID-N-1193 | PG(19:0_20:4) | GP | PG | 1.5736 | 0.0005 | | 0.0066 | | 0.5074 | | -0.9787 | | |  |
| LIPID-N-1314 | PS(18:1_21:2) | GP | PS | 1.4305 | 0.0026 | | 0.0155 | | 0.5070 | | -0.9799 | | |  |
| LIPID-N-1143 | PG(17:0_22:1) | GP | PG | 1.4171 | 0.0024 | | 0.0145 | | 0.5068 | | -0.9804 | | |  |
| LIPID-N-0353 | PE(18:0_18:2) | GP | PE | 1.6888 | 0.0000 | | 0.0036 | | 0.5065 | | -0.9813 | | |  |
| LIPID-N-0194 | LPS(22:1) | GP | LPS | 1.3730 | 0.0032 | | 0.0178 | | 0.5057 | | -0.9837 | | |  |
| LIPID-P-0441 | LPE(P-20:0) | GP | LPE-P | 1.4799 | 0.0008 | | 0.0082 | | 0.5026 | | -0.9925 | | |  |
| LIPID-P-0565 | PC(O-18:2_20:4) | GP | PC-O | 1.4785 | 0.0004 | | 0.0064 | | 0.5004 | | -0.9990 | | |  |
| LIPID-N-1192 | PG(18:2_21:2) | GP | PG | 1.6838 | 0.0000 | | 0.0036 | | 0.5003 | | -0.9990 | | |  |
| LIPID-N-1286 | PS(15:0_18:2) | GP | PS | 1.5023 | 0.0009 | | 0.0083 | | 0.4992 | | -1.0022 | | |  |
| LIPID-N-1175 | PG(18:0_16:3) | GP | PG | 1.6013 | 0.0002 | | 0.0055 | | 0.4986 | | -1.0040 | | |  |
| LIPID-N-0236 | PC(18:0_18:2) | GP | PC | 1.6426 | 0.0002 | | 0.0057 | | 0.4957 | | -1.0125 | | |  |
| LIPID-N-1189 | PG(18:1_18:3) | GP | PG | 1.6023 | 0.0002 | | 0.0055 | | 0.4952 | | -1.0139 | | |  |
| LIPID-N-0447 | PG(18:0_18:2) | GP | PG | 1.4432 | 0.0014 | | 0.0106 | | 0.4951 | | -1.0141 | | |  |
| LIPID-N-0270 | PC(18:2_18:2) | GP | PC | 1.5000 | 0.0016 | | 0.0120 | | 0.4950 | | -1.0144 | | |  |
| LIPID-N-1271 | PS(17:0_21:1) | GP | PS | 1.4989 | 0.0013 | | 0.0101 | | 0.4943 | | -1.0165 | | |  |
| LIPID-N-0490 | PI(18:2_16:0) | GP | PI | 1.5740 | 0.0001 | | 0.0043 | | 0.4933 | | -1.0193 | | |  |
| LIPID-P-1405 | PE(P-18:2_22:6) | GP | PE-P | 1.4433 | 0.0022 | | 0.0135 | | 0.4917 | | -1.0242 | | |  |
| LIPID-P-1534 | LPE(19:0) | GP | LPE | 1.3268 | 0.0037 | | 0.0193 | | 0.4895 | | -1.0307 | | |  |
| LIPID-N-0551 | PI(18:0_22:6) | GP | PI | 1.7282 | 0.0000 | | 0.0018 | | 0.4885 | | -1.0335 | | |  |
| LIPID-N-0234 | PC(17:0_18:2) | GP | PC | 1.5281 | 0.0006 | | 0.0071 | | 0.4872 | | -1.0375 | | |  |
| LIPID-N-0834 | PE(O-17:1_20:5) | GP | PE-O | 1.4076 | 0.0007 | | 0.0074 | | 0.4867 | | -1.0389 | | |  |
| LIPID-N-1168 | PG(24:0_18:2) | GP | PG | 1.7468 | 0.0000 | | 0.0018 | | 0.4849 | | -1.0443 | | |  |
| LIPID-N-1275 | PS(18:0_21:1) | GP | PS | 1.6051 | 0.0003 | | 0.0057 | | 0.4839 | | -1.0473 | | |  |
| LIPID-P-0658 | PE(P-18:1_20:4) | GP | PE-P | 1.4184 | 0.0019 | | 0.0133 | | 0.4833 | | -1.0489 | | |  |
| LIPID-N-0539 | PI(18:0_20:5) | GP | PI | 1.1276 | 0.0092 | | 0.0358 | | 0.4805 | | -1.0575 | | |  |
| LIPID-N-0964 | LNAPE(18:1/N-18:2) | GP | LNAPE | 1.5311 | 0.0004 | | 0.0064 | | 0.4782 | | -1.0642 | | |  |
| LIPID-P-0371 | LPC(18:1/0:0) | GP | LPC | 1.4675 | 0.0020 | | 0.0133 | | 0.4782 | | -1.0645 | | |  |
| LIPID-N-1407 | PI(15:0_19:2) | GP | PI | 1.5839 | 0.0001 | | 0.0046 | | 0.4772 | | -1.0672 | | |  |
| LIPID-N-1140 | PG(16:0_22:1) | GP | PG | 1.5819 | 0.0003 | | 0.0057 | | 0.4769 | | -1.0683 | | |  |
| LIPID-N-1173 | PG(16:0_18:3) | GP | PG | 1.5723 | 0.0002 | | 0.0057 | | 0.4764 | | -1.0697 | | |  |
| LIPID-N-0254 | PC(18:1_18:2) | GP | PC | 1.6682 | 0.0001 | | 0.0043 | | 0.4760 | | -1.0711 | | |  |
| LIPID-N-1041 | PC(18:2_19:2) | GP | PC | 1.4367 | 0.0027 | | 0.0157 | | 0.4755 | | -1.0725 | | |  |
| LIPID-P-1400 | PE(P-18:3_22:4) | GP | PE-P | 1.3915 | 0.0045 | | 0.0217 | | 0.4754 | | -1.0729 | | |  |
| LIPID-N-0314 | PC(18:2_20:5) | GP | PC | 1.4271 | 0.0037 | | 0.0193 | | 0.4719 | | -1.0834 | | |  |
| LIPID-N-0881 | PMeOH(16:0_18:2) | GP | PMeOH | 1.3132 | 0.0060 | | 0.0258 | | 0.4687 | | -1.0932 | | |  |
| LIPID-N-1410 | PI(10:0_26:2) | GP | PI | 1.5353 | 0.0003 | | 0.0057 | | 0.4641 | | -1.1075 | | |  |
| LIPID-P-1375 | PE(P-18:0_20:5) | GP | PE-P | 1.2410 | 0.0041 | | 0.0205 | | 0.4563 | | -1.1318 | | |  |
| LIPID-N-1411 | PI(19:0_17:2) | GP | PI | 1.5553 | 0.0002 | | 0.0057 | | 0.4526 | | -1.1436 | | |  |
| LIPID-N-0616 | LPA(18:0) | GP | LPA | 1.6537 | 0.0001 | | 0.0043 | | 0.4514 | | -1.1474 | | |  |
| LIPID-N-0453 | PG(18:2_18:2) | GP | PG | 1.5200 | 0.0007 | | 0.0074 | | 0.4513 | | -1.1477 | | |  |
| LIPID-P-0408 | LPE(16:0/0:0) | GP | LPE | 1.5950 | 0.0001 | | 0.0046 | | 0.4511 | | -1.1486 | | |  |
| LIPID-P-0564 | PC(O-16:1_20:5) | GP | PC-O | 1.4812 | 0.0004 | | 0.0064 | | 0.4473 | | -1.1606 | | |  |
| LIPID-N-0257 | PC(19:1_18:2) | GP | PC | 1.6810 | 0.0000 | | 0.0036 | | 0.4457 | | -1.1660 | | |  |
| LIPID-N-0373 | PE(20:1_18:2) | GP | PE | 1.7612 | 0.0000 | | 0.0018 | | 0.4455 | | -1.1665 | | |  |
| LIPID-N-0548 | PI(16:0_22:6) | GP | PI | 1.7521 | 0.0000 | | 0.0018 | | 0.4411 | | -1.1808 | | |  |
| LIPID-P-1388 | PE(P-18:2_20:4) | GP | PE-P | 1.3850 | 0.0038 | | 0.0193 | | 0.4411 | | -1.1808 | | |  |
| LIPID-N-1336 | PS(20:1_20:4) | GP | PS | 1.5480 | 0.0004 | | 0.0061 | | 0.4397 | | -1.1856 | | |  |
| LIPID-N-0495 | PI(18:0_18:2) | GP | PI | 1.6173 | 0.0001 | | 0.0043 | | 0.4389 | | -1.1881 | | |  |
| LIPID-P-0657 | PE(P-16:0_20:5) | GP | PE-P | 1.4358 | 0.0008 | | 0.0082 | | 0.4379 | | -1.1913 | | |  |
| LIPID-N-1404 | PI(14:1_18:1) | GP | PI | 1.6474 | 0.0000 | | 0.0036 | | 0.4368 | | -1.1950 | | |  |
| LIPID-N-0424 | PE(20:5_18:2) | GP | PE | 1.3638 | 0.0014 | | 0.0106 | | 0.4362 | | -1.1971 | | |  |
| LIPID-N-0195 | LPS(24:1) | GP | LPS | 1.4390 | 0.0014 | | 0.0104 | | 0.4358 | | -1.1982 | | |  |
| LIPID-N-1278 | PS(18:0_22:1) | GP | PS | 1.5905 | 0.0004 | | 0.0064 | | 0.4344 | | -1.2028 | | |  |
| LIPID-N-0370 | PE(18:2_19:1) | GP | PE | 1.4165 | 0.0028 | | 0.0162 | | 0.4337 | | -1.2052 | | |  |
| LIPID-N-1285 | PS(14:1_19:1) | GP | PS | 1.5643 | 0.0008 | | 0.0079 | | 0.4324 | | -1.2095 | | |  |
| LIPID-P-0640 | PE(P-16:0_18:2) | GP | PE-P | 1.5207 | 0.0017 | | 0.0124 | | 0.4319 | | -1.2113 | | |  |
| LIPID-N-0831 | PE(O-16:1_20:5) | GP | PE-O | 1.4522 | 0.0004 | | 0.0064 | | 0.4307 | | -1.2153 | | |  |
| LIPID-N-1085 | PE(18:2_18:3) | GP | PE | 1.5554 | 0.0004 | | 0.0064 | | 0.4302 | | -1.2169 | | |  |
| LIPID-P-1387 | PE(P-18:1_20:5) | GP | PE-P | 1.4226 | 0.0009 | | 0.0082 | | 0.4206 | | -1.2494 | | |  |
| LIPID-N-0856 | PE(O-18:2_20:5) | GP | PE-O | 1.2948 | 0.0025 | | 0.0147 | | 0.4202 | | -1.2508 | | |  |
| LIPID-N-0556 | PI(18:1_22:6) | GP | PI | 1.7203 | 0.0000 | | 0.0018 | | 0.4194 | | -1.2537 | | |  |
| LIPID-N-1437 | PI(18:2_20:2) | GP | PI | 1.4880 | 0.0003 | | 0.0057 | | 0.4180 | | -1.2583 | | |  |
| LIPID-N-0778 | PE(O-18:2_20:1) | GP | PE-O | 1.6355 | 0.0000 | | 0.0036 | | 0.4167 | | -1.2630 | | |  |
| LIPID-N-1207 | PG(21:1_20:4) | GP | PG | 1.5100 | 0.0037 | | 0.0193 | | 0.4141 | | -1.2719 | | |  |
| LIPID-N-1075 | PE(16:2_18:2) | GP | PE | 1.5107 | 0.0008 | | 0.0079 | | 0.4124 | | -1.2781 | | |  |
| LIPID-N-0193 | LPS(20:1) | GP | LPS | 1.5103 | 0.0005 | | 0.0066 | | 0.4104 | | -1.2850 | | |  |
| LIPID-N-0791 | PE(O-18:2_18:2) | GP | PE-O | 1.5860 | 0.0002 | | 0.0055 | | 0.4103 | | -1.2851 | | |  |
| LIPID-N-1147 | PG(18:0_22:1) | GP | PG | 1.7696 | 0.0000 | | 0.0019 | | 0.4089 | | -1.2902 | | |  |
| LIPID-N-0382 | PE(18:2_18:2) | GP | PE | 1.5783 | 0.0003 | | 0.0060 | | 0.3968 | | -1.3335 | | |  |
| LIPID-N-0869 | PE(O-18:3_20:5) | GP | PE-O | 1.2215 | 0.0047 | | 0.0225 | | 0.3950 | | -1.3400 | | |  |
| LIPID-N-0515 | PI(18:1_20:2) | GP | PI | 1.6496 | 0.0000 | | 0.0036 | | 0.3944 | | -1.3424 | | |  |
| LIPID-N-1071 | PE(18:1_19:2) | GP | PE | 1.5034 | 0.0007 | | 0.0072 | | 0.3916 | | -1.3524 | | |  |
| LIPID-P-1339 | PE(P-18:2_16:1) | GP | PE-P | 1.4446 | 0.0010 | | 0.0089 | | 0.3908 | | -1.3557 | | |  |
| LIPID-P-0115 | Cer(d18:1/22:0) | SP | Cer-NS | 1.4527 | 0.0041 | | 0.0205 | | 0.3893 | | -1.3611 | | |  |
| LIPID-N-0508 | PI(18:1_18:2) | GP | PI | 1.7029 | 0.0000 | | 0.0018 | | 0.3867 | | -1.3707 | | |  |
| LIPID-P-0568 | PC(O-16:1_22:6) | GP | PC-O | 1.3010 | 0.0035 | | 0.0189 | | 0.3819 | | -1.3887 | | |  |
| LIPID-N-0965 | LNAPE(18:2/N-18:2) | GP | LNAPE | 1.5156 | 0.0008 | | 0.0082 | | 0.3805 | | -1.3942 | | |  |
| LIPID-P-1397 | PE(P-18:3_20:4) | GP | PE-P | 1.4094 | 0.0032 | | 0.0179 | | 0.3802 | | -1.3952 | | |  |
| LIPID-P-1396 | PE(P-18:2_20:5) | GP | PE-P | 1.2377 | 0.0065 | | 0.0275 | | 0.3779 | | -1.4038 | | |  |
| LIPID-N-0549 | PI(18:1_20:5) | GP | PI | 1.4610 | 0.0003 | | 0.0057 | | 0.3662 | | -1.4493 | | |  |
| LIPID-P-0445 | LPE(P-18:1) | GP | LPE-P | 1.4490 | 0.0006 | | 0.0070 | | 0.3598 | | -1.4746 | | |  |
| LIPID-P-0418 | LPE(18:1/0:0) | GP | LPE | 1.5520 | 0.0004 | | 0.0064 | | 0.3596 | | -1.4754 | | |  |
| LIPID-N-1342 | PS(18:2_20:4) | GP | PS | 1.5983 | 0.0002 | | 0.0057 | | 0.3556 | | -1.4916 | | |  |
| LIPID-P-1353 | PE(P-18:2_18:2) | GP | PE-P | 1.5581 | 0.0004 | | 0.0061 | | 0.3526 | | -1.5040 | | |  |
| LIPID-N-0808 | PE(O-18:3_18:2) | GP | PE-O | 1.5042 | 0.0005 | | 0.0066 | | 0.3458 | | -1.5319 | | |  |
| LIPID-N-0657 | LPA(18:4) | GP | LPA | 1.2662 | 0.0037 | | 0.0193 | | 0.3438 | | -1.5406 | | |  |
| LIPID-P-0647 | PE(P-18:1_18:2) | GP | PE-P | 1.5459 | 0.0005 | | 0.0070 | | 0.3424 | | -1.5462 | | |  |
|  |  |  |  |  |  | |  | |  | |  | | |  |
|  |  |  |  |  |  | |  | |  | |  | | |  |
|  |  |  |  |  |  | |  | |  | |  | | |  |
|  |  |  |  |  |  | |  | |  | |  | | |  |
|  |  |  |  |  |  | |  | |  | |  | | |  |
|  |  |  |  |  |  | |  | |  | |  | | |  |
|  |  |  |  |  |  | |  | |  | |  | | |  |
|  |  |  |  |  |  | |  | |  | |  | | |  |
|  |  |  |  |  |  | |  | |  | |  | | |  |
|  |  |  |  |  |  | |  | |  | |  | | |  |
|  |  |  |  |  |  | |  | |  | |  | | |  |
|  |  |  |  |  |  | |  | |  | |  | | |  |
|  |  |  |  |  |  | |  | |  | |  | | |  |
|  |  |  |  |  |  | |  | |  | |  | | |  |

| **Supplemental Table S5** | |  |  |  |  |  |  |  |
| --- | --- | --- | --- | --- | --- | --- | --- | --- |
| **Differentially Expressed Lipids Between 5 ppm PFOA and Control KC Mice, sorted by Fold-Change** | | | | | | | |  |
| Index | Compounds | Class I | Class II | VIP | Pvalue | FDR | Fold_Change | Log2FC |
| LIPID-N-0063 | TXB2 | FA | Eicosanoid | 2.037 | 0.000 | 0.010 | 30.767 | 4.943 |
| LIPID-P-2579 | Cer(d24:1/18:0(2OH)) | SP | Cer-AS | 1.367 | 0.004 | 0.032 | 6.328 | 2.662 |
| LIPID-P-1305 | Carnitine C22:5 | FA | CAR | 1.439 | 0.024 | 0.089 | 5.506 | 2.461 |
| LIPID-P-0550 | PC(O-18:1_18:2) | GP | PC-O | 1.586 | 0.000 | 0.010 | 4.494 | 2.168 |
| LIPID-P-1290 | Carnitine C17:1 | FA | CAR | 1.565 | 0.003 | 0.030 | 4.008 | 2.003 |
| LIPID-P-1931 | LPC(16:3) | GP | LPC | 1.448 | 0.006 | 0.039 | 3.993 | 1.998 |
| LIPID-P-2556 | Cer(d20:1/24:1(2OH)) | SP | Cer-AS | 1.542 | 0.001 | 0.015 | 3.727 | 1.898 |
| LIPID-P-2505 | Cer(d18:1/17:0(2OH)) | SP | Cer-AS | 1.581 | 0.006 | 0.038 | 3.580 | 1.840 |
| LIPID-P-1301 | Carnitine C22:3 | FA | CAR | 1.629 | 0.002 | 0.023 | 3.429 | 1.778 |
| LIPID-P-2113 | Cer(d20:2/24:0) | SP | Cer-NS | 1.306 | 0.002 | 0.023 | 3.369 | 1.752 |
| LIPID-N-0560 | PS(16:0_16:0) | GP | PS | 1.342 | 0.014 | 0.061 | 3.350 | 1.744 |
| LIPID-P-2557 | Cer(d20:1/24:2(2OH)) | SP | Cer-AS | 1.243 | 0.006 | 0.038 | 3.193 | 1.675 |
| LIPID-N-1262 | PS(19:0_16:1) | GP | PS | 1.417 | 0.001 | 0.015 | 3.190 | 1.674 |
| LIPID-P-0365 | LPC(26:0) | GP | LPC | 1.117 | 0.004 | 0.032 | 3.153 | 1.657 |
| LIPID-N-0202 | PC(14:0_14:0) | GP | PC | 1.620 | 0.000 | 0.010 | 3.045 | 1.607 |
| LIPID-P-0646 | PE(P-15:0_20:3) | GP | PE-P | 1.373 | 0.011 | 0.055 | 3.043 | 1.605 |
| LIPID-P-1303 | Carnitine C22:4 | FA | CAR | 1.641 | 0.014 | 0.061 | 2.980 | 1.575 |
| LIPID-P-2555 | Cer(d20:1/24:0(2OH)) | SP | Cer-AS | 1.490 | 0.002 | 0.023 | 2.944 | 1.558 |
| LIPID-P-2453 | Cer(t18:1/25:0) | SP | Cer-NP | 1.436 | 0.000 | 0.010 | 2.937 | 1.554 |
| LIPID-N-0823 | PE(O-16:1_24:4) | GP | PE-O | 1.674 | 0.001 | 0.018 | 2.888 | 1.530 |
| LIPID-N-0797 | PE(O-20:0_20:4) | GP | PE-O | 1.963 | 0.000 | 0.010 | 2.863 | 1.517 |
| LIPID-N-0228 | PC(16:1_16:1) | GP | PC | 1.728 | 0.000 | 0.010 | 2.847 | 1.509 |
| LIPID-N-1076 | PE(15:0_20:4) | GP | PE | 1.293 | 0.006 | 0.038 | 2.818 | 1.495 |
| LIPID-N-1181 | PG(19:0_20:3) | GP | PG | 1.297 | 0.009 | 0.049 | 2.797 | 1.484 |
| LIPID-P-2099 | Cer(d19:2/24:0) | SP | Cer-NS | 1.259 | 0.006 | 0.038 | 2.770 | 1.470 |
| LIPID-N-0342 | PE(24:0_16:1) | GP | PE | 1.445 | 0.004 | 0.032 | 2.770 | 1.470 |
| LIPID-N-0214 | PC(14:0_16:1) | GP | PC | 1.626 | 0.002 | 0.024 | 2.757 | 1.463 |
| LIPID-N-1035 | PC(16:1_20:3) | GP | PC | 1.710 | 0.001 | 0.019 | 2.752 | 1.461 |
| LIPID-N-0729 | PE(O-16:0_16:0) | GP | PE-O | 1.362 | 0.002 | 0.023 | 2.752 | 1.460 |
| LIPID-N-0817 | PE(O-18:0_22:5) | GP | PE-O | 1.454 | 0.014 | 0.061 | 2.689 | 1.427 |
| LIPID-P-0040 | Carnitine C16:1-OH | FA | CAR | 1.546 | 0.004 | 0.032 | 2.687 | 1.426 |
| LIPID-N-0763 | PE(O-18:1_22:1) | GP | PE-O | 1.386 | 0.001 | 0.018 | 2.671 | 1.418 |
| LIPID-P-1300 | Carnitine C20:3 | FA | CAR | 1.644 | 0.002 | 0.023 | 2.660 | 1.412 |
| LIPID-N-1231 | PS(14:0_19:0) | GP | PS | 1.290 | 0.021 | 0.079 | 2.641 | 1.401 |
| LIPID-P-0045 | Carnitine C18:1-OH | FA | CAR | 1.375 | 0.009 | 0.049 | 2.627 | 1.393 |
| LIPID-N-0759 | PE(O-16:1_22:1) | GP | PE-O | 1.312 | 0.000 | 0.010 | 2.624 | 1.392 |
| LIPID-P-2102 | Cer(d20:1/23:1) | SP | Cer-NS | 1.466 | 0.007 | 0.042 | 2.620 | 1.389 |
| LIPID-N-1108 | PE(22:5_22:6) | GP | PE | 1.700 | 0.002 | 0.023 | 2.593 | 1.375 |
| LIPID-N-0347 | PE(16:1_16:1) | GP | PE | 1.818 | 0.000 | 0.010 | 2.585 | 1.370 |
| LIPID-P-0039 | Carnitine C16:1 | FA | CAR | 1.485 | 0.006 | 0.039 | 2.550 | 1.350 |
| LIPID-N-0812 | PE(O-16:0_22:5) | GP | PE-O | 1.610 | 0.001 | 0.018 | 2.549 | 1.350 |
| LIPID-N-0732 | PE(O-16:0_20:0) | GP | PE-O | 1.133 | 0.014 | 0.061 | 2.524 | 1.336 |
| LIPID-P-0042 | Carnitine C18:1 | FA | CAR | 1.474 | 0.006 | 0.039 | 2.489 | 1.316 |
| LIPID-P-0051 | Carnitine C18:2-OH | FA | CAR | 1.449 | 0.009 | 0.049 | 2.469 | 1.304 |
| LIPID-N-0200 | LPS(22:5) | GP | LPS | 1.675 | 0.001 | 0.020 | 2.466 | 1.302 |
| LIPID-P-0018 | Carnitine C15:0 | FA | CAR | 1.522 | 0.001 | 0.018 | 2.457 | 1.297 |
| LIPID-P-2552 | Cer(d20:1/22:0(2OH)) | SP | Cer-AS | 1.458 | 0.006 | 0.038 | 2.451 | 1.293 |
| LIPID-N-0792 | PE(O-16:0_22:4) | GP | PE-O | 1.592 | 0.001 | 0.015 | 2.439 | 1.286 |
| LIPID-N-0197 | LPS(20:3) | GP | LPS | 1.878 | 0.000 | 0.010 | 2.426 | 1.278 |
| LIPID-P-0531 | PC(O-16:0_14:0) | GP | PC-O | 1.586 | 0.000 | 0.010 | 2.410 | 1.269 |
| LIPID-N-1010 | PC(14:1_18:1) | GP | PC | 1.151 | 0.006 | 0.038 | 2.331 | 1.221 |
| LIPID-N-0796 | PE(O-18:0_22:4) | GP | PE-O | 1.280 | 0.040 | 0.123 | 2.324 | 1.217 |
| LIPID-P-2423 | Cer(t17:2/16:1) | SP | Cer-NP | 1.228 | 0.002 | 0.023 | 2.315 | 1.211 |
| LIPID-N-0316 | PC(20:3_20:4) | GP | PC | 1.725 | 0.001 | 0.018 | 2.313 | 1.210 |
| LIPID-P-0393 | LPC(O-14:0) | GP | LPC-O | 1.202 | 0.014 | 0.061 | 2.309 | 1.207 |
| LIPID-P-2428 | Cer(t17:2/20:2) | SP | Cer-NP | 1.354 | 0.014 | 0.061 | 2.309 | 1.207 |
| LIPID-P-2433 | Cer(t17:2/24:1) | SP | Cer-NP | 1.570 | 0.004 | 0.034 | 2.291 | 1.196 |
| LIPID-P-0731 | SM(d18:0/17:0) | SP | SM | 1.356 | 0.006 | 0.038 | 2.285 | 1.192 |
| LIPID-P-2553 | Cer(d20:1/23:0(2OH)) | SP | Cer-AS | 1.402 | 0.001 | 0.018 | 2.271 | 1.183 |
| LIPID-N-0788 | PE(O-16:0_20:4) | GP | PE-O | 1.399 | 0.017 | 0.070 | 2.260 | 1.176 |
| LIPID-N-1042 | PC(15:1_22:4) | GP | PC | 1.554 | 0.014 | 0.061 | 2.255 | 1.173 |
| LIPID-N-0392 | PE(18:0_22:4) | GP | PE | 1.423 | 0.040 | 0.123 | 2.246 | 1.167 |
| LIPID-P-0043 | Carnitine C20:1 | FA | CAR | 1.512 | 0.004 | 0.035 | 2.236 | 1.161 |
| LIPID-P-0022 | Carnitine C16-OH | FA | CAR | 1.530 | 0.002 | 0.023 | 2.224 | 1.153 |
| LIPID-P-0748 | SM(d20:0/24:1) | SP | SM | 1.373 | 0.002 | 0.023 | 2.224 | 1.153 |
| LIPID-P-2422 | Cer(t17:2/16:0) | SP | Cer-NP | 1.501 | 0.008 | 0.047 | 2.223 | 1.153 |
| LIPID-P-1282 | Carnitine C17:0 | FA | CAR | 1.343 | 0.006 | 0.038 | 2.205 | 1.141 |
| LIPID-N-0803 | PE(O-24:0_20:4) | GP | PE-O | 1.851 | 0.000 | 0.010 | 2.189 | 1.130 |
| LIPID-N-0793 | PE(O-18:0_20:4) | GP | PE-O | 1.241 | 0.009 | 0.049 | 2.168 | 1.116 |
| LIPID-N-0055 | PGD2 | FA | Eicosanoid | 1.177 | 0.021 | 0.079 | 2.167 | 1.115 |
| LIPID-N-0345 | PE(24:0_18:1) | GP | PE | 1.279 | 0.021 | 0.079 | 2.162 | 1.112 |
| LIPID-P-2399 | Cer(t14:2/26:2) | SP | Cer-NP | 1.542 | 0.005 | 0.038 | 2.153 | 1.107 |
| LIPID-P-0532 | PC(O-16:0_16:0) | GP | PC-O | 1.237 | 0.040 | 0.123 | 2.144 | 1.101 |
| LIPID-P-0732 | SM(d18:0/18:0) | SP | SM | 1.285 | 0.006 | 0.038 | 2.138 | 1.097 |
| LIPID-N-0751 | PE(O-17:1_18:1) | GP | PE-O | 1.142 | 0.004 | 0.032 | 2.127 | 1.089 |
| LIPID-N-1100 | PE(20:3_20:4) | GP | PE | 1.829 | 0.001 | 0.018 | 2.118 | 1.083 |
| LIPID-P-0017 | Carnitine C14:0 | FA | CAR | 1.723 | 0.001 | 0.015 | 2.105 | 1.074 |
| LIPID-P-0566 | PC(O-18:0_22:6) | GP | PC-O | 1.416 | 0.014 | 0.061 | 2.096 | 1.068 |
| LIPID-P-0050 | Carnitine C20:2 | FA | CAR | 1.517 | 0.004 | 0.034 | 2.079 | 1.056 |
| LIPID-N-1106 | PE(20:3_22:6) | GP | PE | 1.613 | 0.004 | 0.032 | 2.073 | 1.052 |
| LIPID-P-0038 | Carnitine C14:1-OH | FA | CAR | 1.455 | 0.008 | 0.047 | 2.066 | 1.047 |
| LIPID-P-0394 | LPC(O-16:0) | GP | LPC-O | 1.497 | 0.004 | 0.032 | 2.063 | 1.045 |
| LIPID-N-1028 | PC(14:1_20:4) | GP | PC | 1.522 | 0.006 | 0.038 | 2.050 | 1.036 |
| LIPID-P-0638 | PE(P-16:0_22:1) | GP | PE-P | 1.042 | 0.040 | 0.123 | 2.029 | 1.021 |
| LIPID-P-0021 | Carnitine C16:0 | FA | CAR | 1.655 | 0.001 | 0.019 | 2.020 | 1.014 |
| LIPID-N-1036 | PC(14:1_22:4) | GP | PC | 1.189 | 0.009 | 0.049 | 2.001 | 1.001 |
| LIPID-N-1363 | PS(20:3_22:4) | GP | PS | 1.299 | 0.006 | 0.038 | 1.999 | 0.999 |
| LIPID-P-0730 | SM(d18:0/16:0) | SP | SM | 1.519 | 0.006 | 0.038 | 1.989 | 0.992 |
| LIPID-P-1291 | Carnitine C19:1 | FA | CAR | 1.372 | 0.011 | 0.057 | 1.987 | 0.990 |
| LIPID-P-0433 | LPE(22:4) | GP | LPE | 1.279 | 0.009 | 0.049 | 1.965 | 0.974 |
| LIPID-N-0389 | PE(16:0_22:4) | GP | PE | 1.720 | 0.001 | 0.018 | 1.962 | 0.973 |
| LIPID-N-1310 | PS(19:0_18:3) | GP | PS | 1.479 | 0.009 | 0.049 | 1.958 | 0.969 |
| LIPID-P-0020 | Carnitine C14-OH | FA | CAR | 1.623 | 0.002 | 0.023 | 1.952 | 0.965 |
| LIPID-P-1298 | Carnitine C16:3 | FA | CAR | 1.414 | 0.029 | 0.098 | 1.948 | 0.962 |
| LIPID-N-1252 | PS(18:0_24:0) | GP | PS | 1.424 | 0.021 | 0.079 | 1.946 | 0.961 |
| LIPID-N-0203 | PC(16:0_14:0) | GP | PC | 1.547 | 0.004 | 0.032 | 1.944 | 0.959 |
| LIPID-P-0431 | LPE(22:3) | GP | LPE | 1.289 | 0.014 | 0.061 | 1.940 | 0.956 |
| LIPID-P-1513 | Cer(t18:0/24:0(2OH)) | SP | Cer-AP | 1.155 | 0.040 | 0.123 | 1.934 | 0.952 |
| LIPID-N-0742 | PE(O-18:0_20:1) | GP | PE-O | 1.232 | 0.021 | 0.079 | 1.931 | 0.949 |
| LIPID-P-2442 | Cer(t18:0/16:0) | SP | Cer-NP | 1.350 | 0.021 | 0.080 | 1.922 | 0.942 |
| LIPID-P-1376 | PE(P-16:0_24:5) | GP | PE-P | 1.431 | 0.006 | 0.038 | 1.920 | 0.941 |
| LIPID-N-0764 | PE(O-22:0_18:2) | GP | PE-O | 1.049 | 0.009 | 0.049 | 1.917 | 0.939 |
| LIPID-N-0801 | PE(O-20:0_22:4) | GP | PE-O | 1.110 | 0.009 | 0.049 | 1.915 | 0.937 |
| LIPID-P-0014 | Carnitine C12:0 | FA | CAR | 1.616 | 0.002 | 0.023 | 1.912 | 0.935 |
| LIPID-P-0563 | PC(O-24:1_20:4) | GP | PC-O | 1.715 | 0.001 | 0.018 | 1.906 | 0.931 |
| LIPID-P-2105 | Cer(d20:1/24:2) | SP | Cer-NS | 1.094 | 0.029 | 0.098 | 1.888 | 0.917 |
| LIPID-P-1930 | LPC(24:2) | GP | LPC | 1.252 | 0.009 | 0.049 | 1.887 | 0.916 |
| LIPID-N-0986 | PC(13:0_16:1) | GP | PC | 1.461 | 0.009 | 0.049 | 1.885 | 0.915 |
| LIPID-N-0343 | PE(22:0_18:1) | GP | PE | 1.291 | 0.029 | 0.098 | 1.882 | 0.913 |
| LIPID-N-1283 | PS(21:0_24:1) | GP | PS | 1.398 | 0.015 | 0.065 | 1.876 | 0.907 |
| LIPID-N-0731 | PE(O-20:0_16:0) | GP | PE-O | 1.583 | 0.002 | 0.023 | 1.865 | 0.899 |
| LIPID-N-1083 | PE(24:0_20:4) | GP | PE | 1.290 | 0.029 | 0.098 | 1.853 | 0.889 |
| LIPID-N-1057 | PE(18:0_22:1) | GP | PE | 1.557 | 0.004 | 0.032 | 1.849 | 0.887 |
| LIPID-N-0749 | PE(O-16:0_18:2) | GP | PE-O | 1.377 | 0.025 | 0.092 | 1.848 | 0.886 |
| LIPID-N-0862 | PE(O-18:3_22:4) | GP | PE-O | 1.433 | 0.021 | 0.079 | 1.845 | 0.884 |
| LIPID-P-2441 | Cer(t18:0/15:1) | SP | Cer-NP | 1.304 | 0.027 | 0.097 | 1.843 | 0.882 |
| LIPID-P-1943 | Cer(t15:0/18:1(2OH)) | SP | Cer-AP | 1.287 | 0.027 | 0.096 | 1.835 | 0.876 |
| LIPID-P-0554 | PC(O-16:0_20:4) | GP | PC-O | 1.389 | 0.021 | 0.079 | 1.830 | 0.872 |
| LIPID-N-1237 | PS(16:0_19:0) | GP | PS | 1.260 | 0.009 | 0.049 | 1.813 | 0.859 |
| LIPID-N-0059 | PGD1 | FA | Eicosanoid | 1.453 | 0.009 | 0.049 | 1.812 | 0.858 |
| LIPID-N-0595 | PS(18:0_22:5) | GP | PS | 1.552 | 0.005 | 0.038 | 1.807 | 0.853 |
| LIPID-P-1929 | LPC(16:2) | GP | LPC | 1.435 | 0.021 | 0.079 | 1.802 | 0.850 |
| LIPID-N-1019 | PC(15:1_18:1) | GP | PC | 1.353 | 0.002 | 0.023 | 1.798 | 0.846 |
| LIPID-P-0037 | Carnitine C14:1 | FA | CAR | 1.273 | 0.024 | 0.088 | 1.783 | 0.835 |
| LIPID-N-0344 | PE(22:1_18:0) | GP | PE | 1.521 | 0.004 | 0.032 | 1.770 | 0.824 |
| LIPID-N-0377 | PE(18:1_22:2) | GP | PE | 1.118 | 0.014 | 0.061 | 1.765 | 0.820 |
| LIPID-N-0458 | PG(16:1_20:4) | GP | PG | 1.251 | 0.027 | 0.097 | 1.764 | 0.819 |
| LIPID-P-0094 | SPH(d18:0) | SP | SPH | 1.377 | 0.018 | 0.074 | 1.761 | 0.817 |
| LIPID-N-0278 | PC(20:1_20:3) | GP | PC | 1.646 | 0.002 | 0.025 | 1.751 | 0.808 |
| LIPID-P-2429 | Cer(t17:2/22:0) | SP | Cer-NP | 1.233 | 0.006 | 0.038 | 1.742 | 0.801 |
| LIPID-N-0730 | PE(O-18:0_16:0) | GP | PE-O | 1.409 | 0.040 | 0.123 | 1.735 | 0.795 |
| LIPID-P-1940 | Cer(t14:0/19:1(2OH)) | SP | Cer-AP | 1.227 | 0.041 | 0.126 | 1.734 | 0.794 |
| LIPID-N-0783 | PE(O-18:1_22:2) | GP | PE-O | 1.415 | 0.006 | 0.038 | 1.732 | 0.793 |
| LIPID-P-0015 | Carnitine C13:0 | FA | CAR | 1.655 | 0.001 | 0.018 | 1.732 | 0.792 |
| LIPID-P-2472 | Cer(d14:1/19:1(2OH)) | SP | Cer-AS | 1.277 | 0.032 | 0.107 | 1.730 | 0.791 |
| LIPID-N-0216 | PC(16:0_16:1) | GP | PC | 1.544 | 0.002 | 0.023 | 1.715 | 0.779 |
| LIPID-P-0406 | LPC(O-20:3) | GP | LPC-O | 1.187 | 0.004 | 0.032 | 1.709 | 0.774 |
| LIPID-N-1012 | PC(15:0_18:0) | GP | PC | 1.353 | 0.021 | 0.080 | 1.707 | 0.771 |
| LIPID-N-1270 | PS(21:0_16:1) | GP | PS | 1.340 | 0.021 | 0.079 | 1.704 | 0.769 |
| LIPID-N-1347 | PS(20:3_20:3) | GP | PS | 1.792 | 0.001 | 0.016 | 1.696 | 0.762 |
| LIPID-N-0243 | PC(18:1_24:1) | GP | PC | 1.416 | 0.040 | 0.123 | 1.692 | 0.758 |
| LIPID-P-0368 | LPC(16:1) | GP | LPC | 1.550 | 0.003 | 0.030 | 1.689 | 0.756 |
| LIPID-N-1357 | PS(18:3_20:4) | GP | PS | 1.354 | 0.009 | 0.049 | 1.685 | 0.753 |
| LIPID-N-0380 | PE(20:4_14:0) | GP | PE | 1.307 | 0.025 | 0.091 | 1.682 | 0.751 |
| LIPID-N-0820 | PE(O-18:1_22:4) | GP | PE-O | 1.406 | 0.014 | 0.062 | 1.678 | 0.747 |
| LIPID-N-0570 | PS(18:0_18:1) | GP | PS | 1.583 | 0.003 | 0.031 | 1.671 | 0.741 |
| LIPID-P-1302 | Carnitine C20:4 | FA | CAR | 1.502 | 0.006 | 0.039 | 1.664 | 0.735 |
| LIPID-P-1297 | Carnitine C22:2 | FA | CAR | 1.405 | 0.011 | 0.054 | 1.661 | 0.732 |
| LIPID-N-0816 | PE(O-17:1_22:4) | GP | PE-O | 1.289 | 0.026 | 0.095 | 1.661 | 0.732 |
| LIPID-N-0363 | PE(18:1_24:1) | GP | PE | 1.296 | 0.040 | 0.123 | 1.660 | 0.731 |
| LIPID-P-1345 | PE(P-16:0_22:3) | GP | PE-P | 1.274 | 0.029 | 0.098 | 1.654 | 0.726 |
| LIPID-N-0359 | PE(22:2_18:0) | GP | PE | 1.702 | 0.004 | 0.032 | 1.650 | 0.722 |
| LIPID-N-1269 | PS(19:0_18:1) | GP | PS | 1.416 | 0.006 | 0.038 | 1.634 | 0.709 |
| LIPID-N-0598 | PS(18:0_22:6) | GP | PS | 1.254 | 0.039 | 0.123 | 1.626 | 0.701 |
| LIPID-N-0738 | PE(O-18:0_18:1) | GP | PE-O | 1.194 | 0.050 | 0.146 | 1.623 | 0.699 |
| LIPID-N-0459 | PG(18:1_20:4) | GP | PG | 1.402 | 0.015 | 0.064 | 1.620 | 0.696 |
| LIPID-N-0609 | CerP(d18:1/18:1) | SP | CerP | 1.539 | 0.004 | 0.034 | 1.619 | 0.695 |
| LIPID-N-0331 | PE(16:1_16:0) | GP | PE | 1.721 | 0.001 | 0.018 | 1.618 | 0.695 |
| LIPID-N-0362 | PE(18:1_22:1) | GP | PE | 1.298 | 0.040 | 0.123 | 1.618 | 0.695 |
| LIPID-P-0036 | Carnitine C12:1-OH | FA | CAR | 1.308 | 0.021 | 0.081 | 1.615 | 0.692 |
| LIPID-P-2412 | Cer(t17:1/16:0) | SP | Cer-NP | 1.511 | 0.007 | 0.043 | 1.612 | 0.689 |
| LIPID-P-0013 | Carnitine C10:0 | FA | CAR | 1.127 | 0.046 | 0.137 | 1.612 | 0.689 |
| LIPID-N-0585 | PS(19:0_20:3) | GP | PS | 1.363 | 0.025 | 0.092 | 1.608 | 0.686 |
| LIPID-N-0041 | 11(S)-HETE | FA | Eicosanoid | 1.232 | 0.035 | 0.115 | 1.602 | 0.680 |
| LIPID-N-0995 | PC(14:0_17:0) | GP | PC | 1.386 | 0.019 | 0.077 | 1.602 | 0.680 |
| LIPID-P-0025 | Carnitine C18-OH | FA | CAR | 1.233 | 0.033 | 0.109 | 1.601 | 0.679 |
| LIPID-P-2431 | Cer(t17:2/23:0) | SP | Cer-NP | 1.384 | 0.016 | 0.068 | 1.589 | 0.668 |
| LIPID-N-1360 | PS(18:2_22:5) | GP | PS | 1.547 | 0.004 | 0.033 | 1.588 | 0.667 |
| LIPID-N-0192 | LPS(18:1) | GP | LPS | 1.397 | 0.006 | 0.038 | 1.587 | 0.666 |
| LIPID-P-2506 | Cer(d18:1/18:0(2OH)) | SP | Cer-AS | 1.173 | 0.049 | 0.144 | 1.581 | 0.661 |
| LIPID-N-0301 | PC(22:5_14:1) | GP | PC | 1.466 | 0.012 | 0.059 | 1.565 | 0.646 |
| LIPID-N-0322 | PE(16:0_14:0) | GP | PE | 1.336 | 0.023 | 0.087 | 1.562 | 0.643 |
| LIPID-P-0736 | SM(d18:1/14:0) | SP | SM | 1.607 | 0.002 | 0.021 | 1.552 | 0.634 |
| LIPID-N-0666 | LPG(20:3) | GP | LPG | 1.296 | 0.028 | 0.098 | 1.551 | 0.633 |
| LIPID-N-0276 | PC(22:4_16:0) | GP | PC | 1.515 | 0.040 | 0.123 | 1.547 | 0.629 |
| LIPID-N-0231 | PC(16:1_18:1) | GP | PC | 1.665 | 0.002 | 0.022 | 1.540 | 0.622 |
| LIPID-N-0369 | PE(16:0_20:3) | GP | PE | 1.633 | 0.003 | 0.030 | 1.536 | 0.619 |
| LIPID-N-1243 | PS(18:0_19:0) | GP | PS | 1.260 | 0.021 | 0.079 | 1.526 | 0.609 |
| LIPID-N-0958 | LNAPE(16:1/N-16:0) | GP | LNAPE | 1.705 | 0.001 | 0.018 | 1.523 | 0.607 |
| LIPID-P-0753 | SM(d18:1/19:1) | SP | SM | 1.229 | 0.006 | 0.038 | 1.521 | 0.605 |
| LIPID-N-0340 | PE(20:0_18:1) | GP | PE | 1.332 | 0.029 | 0.098 | 1.517 | 0.602 |
| LIPID-N-0846 | PE(O-20:0_22:6) | GP | PE-O | 1.009 | 0.029 | 0.098 | 1.516 | 0.600 |
| LIPID-P-0553 | PC(O-14:0_20:4) | GP | PC-O | 1.392 | 0.006 | 0.038 | 1.513 | 0.598 |
| LIPID-N-0403 | PE(18:0_22:5) | GP | PE | 1.528 | 0.006 | 0.038 | 1.508 | 0.592 |
| LIPID-P-1382 | PE(P-18:0_24:5) | GP | PE-P | 1.098 | 0.040 | 0.123 | 1.503 | 0.588 |
| LIPID-N-0268 | PC(14:0_20:4) | GP | PC | 1.176 | 0.046 | 0.137 | 1.496 | 0.581 |
| LIPID-P-2501 | Cer(d18:1/15:1(2OH)) | SP | Cer-AS | 1.278 | 0.034 | 0.112 | 1.491 | 0.576 |
| LIPID-P-0429 | LPE(0:0/20:3) | GP | LPE | 1.433 | 0.004 | 0.032 | 1.490 | 0.575 |
| LIPID-N-0285 | PC(20:4_16:1) | GP | PC | 1.436 | 0.014 | 0.061 | 1.488 | 0.574 |
| LIPID-N-0396 | PE(16:1_20:4) | GP | PE | 1.228 | 0.040 | 0.123 | 1.487 | 0.572 |
| LIPID-P-0006 | Carnitine C4-OH | FA | CAR | 1.303 | 0.026 | 0.094 | 1.479 | 0.565 |
| LIPID-N-1115 | PG(16:0_20:0) | GP | PG | 1.282 | 0.027 | 0.097 | 1.471 | 0.557 |
| LIPID-N-1104 | PE(20:4_20:4) | GP | PE | 1.271 | 0.014 | 0.061 | 1.465 | 0.551 |
| LIPID-N-1206 | PG(19:2_20:3) | GP | PG | 1.473 | 0.010 | 0.053 | 1.460 | 0.546 |
| LIPID-N-0245 | PC(14:1_18:2) | GP | PC | 1.295 | 0.002 | 0.023 | 1.457 | 0.543 |
| LIPID-N-0372 | PE(20:3_18:0) | GP | PE | 1.561 | 0.006 | 0.038 | 1.456 | 0.542 |
| LIPID-P-0765 | SM(d18:2/24:1) | SP | SM | 1.479 | 0.008 | 0.047 | 1.437 | 0.523 |
| LIPID-P-0026 | Carnitine C3:1-2OH | FA | CAR | 1.220 | 0.042 | 0.127 | 1.434 | 0.520 |
| LIPID-N-1191 | PG(18:2_20:2) | GP | PG | 1.360 | 0.015 | 0.064 | 1.415 | 0.501 |
| LIPID-N-0169 | LPG(16:1) | GP | LPG | 1.562 | 0.001 | 0.018 | 1.414 | 0.500 |
| LIPID-P-1567 | SM(d18:2/24:2) | SP | SM | 1.373 | 0.029 | 0.098 | 1.405 | 0.491 |
| LIPID-P-0350 | LPC(14:0) | GP | LPC | 1.194 | 0.034 | 0.112 | 1.401 | 0.487 |
| LIPID-N-1002 | PC(10:0_22:0) | GP | PC | 1.365 | 0.014 | 0.061 | 1.399 | 0.484 |
| LIPID-N-0668 | LPG(22:4) | GP | LPG | 1.177 | 0.045 | 0.134 | 1.398 | 0.484 |
| LIPID-N-1080 | PE(15:1_22:3) | GP | PE | 1.263 | 0.028 | 0.098 | 1.398 | 0.484 |
| LIPID-N-0567 | PS(16:0_18:1) | GP | PS | 1.240 | 0.036 | 0.117 | 1.396 | 0.482 |
| LIPID-N-1088 | PE(16:1_22:4) | GP | PE | 1.259 | 0.024 | 0.090 | 1.395 | 0.480 |
| LIPID-P-0384 | LPC(20:3) | GP | LPC | 1.243 | 0.029 | 0.098 | 1.387 | 0.472 |
| LIPID-N-0789 | PE(O-16:1_20:3) | GP | PE-O | 1.236 | 0.027 | 0.096 | 1.385 | 0.470 |
| LIPID-N-0388 | PE(18:1_20:3) | GP | PE | 1.549 | 0.006 | 0.039 | 1.384 | 0.469 |
| LIPID-P-0024 | Carnitine C20:0 | FA | CAR | 1.190 | 0.036 | 0.117 | 1.379 | 0.464 |
| LIPID-N-0204 | PC(18:0_12:0) | GP | PC | 1.279 | 0.028 | 0.098 | 1.372 | 0.457 |
| LIPID-N-1086 | PE(15:1_22:4) | GP | PE | 1.300 | 0.023 | 0.087 | 1.372 | 0.456 |
| LIPID-N-1196 | PG(20:0_20:4) | GP | PG | 1.211 | 0.040 | 0.123 | 1.354 | 0.437 |
| LIPID-N-0613 | CerP(d18:1/20:3) | SP | CerP | 1.194 | 0.040 | 0.123 | 1.350 | 0.433 |
| LIPID-P-0415 | LPE(16:1) | GP | LPE | 1.206 | 0.035 | 0.115 | 1.336 | 0.418 |
| LIPID-N-0217 | PC(14:0_18:1) | GP | PC | 1.314 | 0.017 | 0.072 | 1.318 | 0.398 |
| LIPID-N-0682 | LPI(18:3) | GP | LPI | 1.217 | 0.036 | 0.117 | 1.316 | 0.396 |
| LIPID-N-0180 | LPI(20:3) | GP | LPI | 1.262 | 0.032 | 0.107 | 1.310 | 0.389 |
| LIPID-N-0274 | PC(20:3_18:1) | GP | PC | 1.693 | 0.002 | 0.023 | 1.295 | 0.373 |
| LIPID-N-0294 | PC(18:2_20:3) | GP | PC | 1.399 | 0.018 | 0.074 | 1.281 | 0.357 |
| LIPID-N-0296 | PC(18:0_22:5) | GP | PC | 1.366 | 0.019 | 0.077 | 1.274 | 0.349 |
| LIPID-N-1092 | PE(20:1_20:4) | GP | PE | 1.515 | 0.004 | 0.032 | 1.263 | 0.336 |
| LIPID-P-0159 | CoenzymeQ8 | PR | CoQ | 1.320 | 0.018 | 0.074 | 1.255 | 0.328 |
| LIPID-N-1033 | PC(18:1_17:2) | GP | PC | 1.192 | 0.041 | 0.124 | 1.184 | 0.244 |
| LIPID-N-0665 | LPG(20:2) | GP | LPG | 1.317 | 0.017 | 0.072 | 0.840 | -0.252 |
| LIPID-N-0235 | PC(18:1_18:1) | GP | PC | 1.428 | 0.011 | 0.056 | 0.833 | -0.264 |
| LIPID-N-0755 | PE(O-16:1_20:1) | GP | PE-O | 1.203 | 0.048 | 0.141 | 0.825 | -0.277 |
| LIPID-N-0336 | PE(17:0_18:1) | GP | PE | 1.216 | 0.038 | 0.122 | 0.823 | -0.282 |
| LIPID-N-0814 | PE(O-18:1_20:4) | GP | PE-O | 1.429 | 0.009 | 0.049 | 0.813 | -0.298 |
| LIPID-N-0809 | PE(O-16:1_20:4) | GP | PE-O | 1.308 | 0.025 | 0.092 | 0.806 | -0.311 |
| LIPID-N-1335 | PS(18:1_22:4) | GP | PS | 1.267 | 0.026 | 0.095 | 0.802 | -0.319 |
| LIPID-N-1120 | PG(16:0_22:0) | GP | PG | 1.518 | 0.007 | 0.043 | 0.800 | -0.322 |
| LIPID-N-0794 | PE(O-18:1_20:3) | GP | PE-O | 1.372 | 0.013 | 0.061 | 0.798 | -0.325 |
| LIPID-N-0760 | PE(O-20:1_18:1) | GP | PE-O | 1.182 | 0.045 | 0.134 | 0.797 | -0.327 |
| LIPID-N-0973 | LNAPE(22:6/N-20:1) | GP | LNAPE | 1.116 | 0.044 | 0.133 | 0.794 | -0.333 |
| LIPID-N-0175 | LPI(16:0) | GP | LPI | 1.418 | 0.013 | 0.061 | 0.792 | -0.337 |
| LIPID-N-1200 | PG(22:0_20:4) | GP | PG | 1.377 | 0.014 | 0.061 | 0.790 | -0.341 |
| LIPID-N-1183 | PG(22:1_18:2) | GP | PG | 1.179 | 0.043 | 0.130 | 0.786 | -0.348 |
| LIPID-N-1220 | PG(20:1_22:6) | GP | PG | 1.267 | 0.029 | 0.098 | 0.784 | -0.351 |
| LIPID-N-0860 | PE(O-20:3_20:4) | GP | PE-O | 1.299 | 0.024 | 0.088 | 0.781 | -0.356 |
| LIPID-N-0399 | PE(18:1_20:4) | GP | PE | 1.192 | 0.042 | 0.127 | 0.777 | -0.365 |
| LIPID-N-0825 | PE(O-22:1_20:4) | GP | PE-O | 1.444 | 0.007 | 0.042 | 0.776 | -0.365 |
| LIPID-N-0960 | LNAPE(18:1/N-17:0) | GP | LNAPE | 1.452 | 0.008 | 0.047 | 0.775 | -0.368 |
| LIPID-P-0535 | PC(O-16:0_22:0) | GP | PC-O | 1.256 | 0.035 | 0.115 | 0.769 | -0.380 |
| LIPID-N-0758 | PE(O-18:1_20:1) | GP | PE-O | 1.421 | 0.011 | 0.055 | 0.764 | -0.389 |
| LIPID-N-0455 | PG(18:0_20:4) | GP | PG | 1.335 | 0.015 | 0.064 | 0.758 | -0.399 |
| LIPID-N-1169 | PG(20:1_22:1) | GP | PG | 1.261 | 0.028 | 0.098 | 0.757 | -0.401 |
| LIPID-N-0386 | PE(17:0_20:4) | GP | PE | 1.340 | 0.016 | 0.066 | 0.755 | -0.406 |
| LIPID-N-0773 | PE(O-18:1_18:2) | GP | PE-O | 1.544 | 0.005 | 0.037 | 0.755 | -0.406 |
| LIPID-P-0030 | Carnitine C5:1-2OH | FA | CAR | 1.382 | 0.009 | 0.049 | 0.754 | -0.407 |
| LIPID-P-0011 | Carnitine C6-2OH | FA | CAR | 1.409 | 0.014 | 0.062 | 0.751 | -0.414 |
| LIPID-N-1148 | PG(22:0_18:1) | GP | PG | 1.602 | 0.003 | 0.030 | 0.750 | -0.416 |
| LIPID-N-0871 | PE(O-18:2_22:6) | GP | PE-O | 1.508 | 0.001 | 0.018 | 0.747 | -0.421 |
| LIPID-N-0524 | PI(18:0_20:4) | GP | PI | 1.330 | 0.019 | 0.077 | 0.746 | -0.424 |
| LIPID-N-1393 | PI(19:0_15:1) | GP | PI | 1.118 | 0.050 | 0.145 | 0.745 | -0.425 |
| LIPID-P-0378 | LPC(0:0/18:2) | GP | LPC | 1.203 | 0.049 | 0.145 | 0.744 | -0.426 |
| LIPID-N-1072 | PE(18:1_20:2) | GP | PE | 1.281 | 0.025 | 0.092 | 0.742 | -0.431 |
| LIPID-N-0787 | PE(O-24:1_18:2) | GP | PE-O | 1.205 | 0.031 | 0.102 | 0.740 | -0.435 |
| LIPID-P-0379 | LPC(18:2/0:0) | GP | LPC | 1.199 | 0.048 | 0.142 | 0.737 | -0.440 |
| LIPID-N-1295 | PS(18:0_19:2) | GP | PS | 1.419 | 0.008 | 0.047 | 0.736 | -0.443 |
| LIPID-P-0745 | SM(d18:1/23:0) | SP | SM | 1.574 | 0.004 | 0.034 | 0.734 | -0.446 |
| LIPID-N-0181 | LPI(20:4) | GP | LPI | 1.388 | 0.010 | 0.050 | 0.731 | -0.453 |
| LIPID-N-1423 | PI(18:0_20:3) | GP | PI | 1.380 | 0.013 | 0.061 | 0.728 | -0.458 |
| LIPID-P-0437 | LPE(22:6) | GP | LPE | 1.194 | 0.035 | 0.114 | 0.727 | -0.461 |
| LIPID-N-0433 | PG(18:0_16:0) | GP | PG | 1.141 | 0.045 | 0.135 | 0.726 | -0.462 |
| LIPID-N-0426 | PE(22:6_18:1) | GP | PE | 1.348 | 0.016 | 0.069 | 0.725 | -0.463 |
| LIPID-P-0096 | SPH(d18:1) | SP | SPH | 1.361 | 0.013 | 0.061 | 0.725 | -0.465 |
| LIPID-N-0368 | PE(18:1_18:2) | GP | PE | 1.317 | 0.023 | 0.087 | 0.724 | -0.466 |
| LIPID-N-0303 | PC(16:0_22:6) | GP | PC | 1.450 | 0.009 | 0.049 | 0.723 | -0.467 |
| LIPID-N-1416 | PI(16:0_18:3) | GP | PI | 1.169 | 0.041 | 0.125 | 0.722 | -0.471 |
| LIPID-N-0502 | PI(18:0_20:2) | GP | PI | 1.301 | 0.022 | 0.085 | 0.721 | -0.471 |
| LIPID-N-0232 | PC(16:0_18:2) | GP | PC | 1.339 | 0.018 | 0.075 | 0.720 | -0.473 |
| LIPID-N-1392 | PI(15:0_19:1) | GP | PI | 1.252 | 0.029 | 0.098 | 0.718 | -0.478 |
| LIPID-N-1303 | PS(23:0_18:2) | GP | PS | 1.514 | 0.005 | 0.038 | 0.718 | -0.479 |
| LIPID-N-0196 | LPS(18:2) | GP | LPS | 1.414 | 0.010 | 0.053 | 0.717 | -0.479 |
| LIPID-P-0652 | PE(P-16:0_20:4) | GP | PE-P | 1.258 | 0.030 | 0.101 | 0.712 | -0.490 |
| LIPID-N-1194 | PG(18:1_22:3) | GP | PG | 1.337 | 0.017 | 0.072 | 0.711 | -0.492 |
| LIPID-N-0968 | LNAPE(20:4/N-18:2) | GP | LNAPE | 1.343 | 0.015 | 0.065 | 0.711 | -0.493 |
| LIPID-N-0469 | PI(16:0_18:0) | GP | PI | 1.202 | 0.038 | 0.123 | 0.711 | -0.493 |
| LIPID-N-0350 | PE(18:2_16:0) | GP | PE | 1.149 | 0.048 | 0.142 | 0.708 | -0.497 |
| LIPID-N-1170 | PG(22:0_20:2) | GP | PG | 1.393 | 0.013 | 0.061 | 0.708 | -0.498 |
| LIPID-N-0413 | PE(22:6_16:0) | GP | PE | 1.427 | 0.009 | 0.049 | 0.708 | -0.498 |
| LIPID-N-1314 | PS(18:1_21:2) | GP | PS | 1.209 | 0.045 | 0.134 | 0.707 | -0.500 |
| LIPID-N-0777 | PE(O-20:1_18:2) | GP | PE-O | 1.531 | 0.004 | 0.034 | 0.706 | -0.503 |
| LIPID-N-1287 | PS(16:0_17:2) | GP | PS | 1.459 | 0.009 | 0.049 | 0.703 | -0.508 |
| LIPID-N-1224 | PG(22:5_22:6) | GP | PG | 1.209 | 0.044 | 0.133 | 0.701 | -0.512 |
| LIPID-N-1007 | PC(17:0_15:1) | GP | PC | 1.159 | 0.040 | 0.123 | 0.700 | -0.514 |
| LIPID-N-1437 | PI(18:2_20:2) | GP | PI | 1.297 | 0.021 | 0.081 | 0.699 | -0.517 |
| LIPID-N-1168 | PG(24:0_18:2) | GP | PG | 1.544 | 0.005 | 0.038 | 0.696 | -0.524 |
| LIPID-N-1407 | PI(15:0_19:2) | GP | PI | 1.242 | 0.034 | 0.111 | 0.696 | -0.524 |
| LIPID-P-0540 | PC(O-16:0_22:1) | GP | PC-O | 1.707 | 0.004 | 0.032 | 0.695 | -0.526 |
| LIPID-N-0815 | PE(O-19:1_20:4) | GP | PE-O | 1.364 | 0.014 | 0.063 | 0.694 | -0.526 |
| LIPID-N-1208 | PG(22:0_20:5) | GP | PG | 1.185 | 0.032 | 0.106 | 0.690 | -0.535 |
| LIPID-N-0837 | PE(O-18:2_20:4) | GP | PE-O | 1.593 | 0.003 | 0.030 | 0.689 | -0.537 |
| LIPID-N-0858 | PE(O-17:1_22:6) | GP | PE-O | 1.391 | 0.009 | 0.049 | 0.686 | -0.544 |
| LIPID-N-0176 | LPI(18:0) | GP | LPI | 1.820 | 0.000 | 0.010 | 0.686 | -0.544 |
| LIPID-N-0857 | PE(O-16:1_22:6) | GP | PE-O | 1.537 | 0.005 | 0.038 | 0.684 | -0.547 |
| LIPID-N-0811 | PE(O-17:1_20:4) | GP | PE-O | 1.278 | 0.024 | 0.088 | 0.683 | -0.550 |
| LIPID-N-0865 | PE(O-20:1_22:6) | GP | PE-O | 1.537 | 0.003 | 0.030 | 0.682 | -0.553 |
| LIPID-N-1267 | PS(16:0_21:1) | GP | PS | 1.436 | 0.009 | 0.049 | 0.681 | -0.555 |
| LIPID-N-0440 | PG(18:0_18:1) | GP | PG | 1.687 | 0.001 | 0.016 | 0.679 | -0.558 |
| LIPID-N-0179 | LPI(18:2) | GP | LPI | 1.533 | 0.004 | 0.033 | 0.679 | -0.559 |
| LIPID-P-0423 | LPE(0:0/18:2) | GP | LPE | 1.199 | 0.045 | 0.135 | 0.678 | -0.561 |
| LIPID-N-0964 | LNAPE(18:1/N-18:2) | GP | LNAPE | 1.271 | 0.027 | 0.097 | 0.677 | -0.563 |
| LIPID-N-0523 | PI(17:0_20.4) | GP | PI | 1.682 | 0.001 | 0.020 | 0.676 | -0.564 |
| LIPID-N-1337 | PS(21:0_20:5) | GP | PS | 1.317 | 0.017 | 0.070 | 0.675 | -0.567 |
| LIPID-N-1216 | PG(22:0_22:6) | GP | PG | 1.217 | 0.039 | 0.123 | 0.675 | -0.567 |
| LIPID-P-0389 | LPC(20:5) | GP | LPC | 1.364 | 0.015 | 0.064 | 0.674 | -0.568 |
| LIPID-N-0478 | PI(16:0_18:1) | GP | PI | 1.563 | 0.004 | 0.034 | 0.674 | -0.569 |
| LIPID-N-1427 | PI(15:1_18:3) | GP | PI | 1.689 | 0.001 | 0.020 | 0.672 | -0.574 |
| LIPID-N-1285 | PS(14:1_19:1) | GP | PS | 1.275 | 0.029 | 0.098 | 0.672 | -0.575 |
| LIPID-N-1232 | PS(15:0_18:0) | GP | PS | 1.363 | 0.014 | 0.061 | 0.670 | -0.577 |
| LIPID-N-1162 | PG(18:0_20:2) | GP | PG | 1.369 | 0.010 | 0.052 | 0.668 | -0.582 |
| LIPID-N-0770 | PE(O-17:1_18:2) | GP | PE-O | 1.285 | 0.029 | 0.098 | 0.665 | -0.588 |
| LIPID-N-0451 | PG(18:2_18:1) | GP | PG | 1.516 | 0.006 | 0.038 | 0.661 | -0.596 |
| LIPID-N-1175 | PG(18:0_16:3) | GP | PG | 1.607 | 0.003 | 0.026 | 0.661 | -0.597 |
| LIPID-N-0681 | LPI(17:0) | GP | LPI | 1.703 | 0.002 | 0.023 | 0.661 | -0.598 |
| LIPID-N-1432 | PI(15:0_22:4) | GP | PI | 1.519 | 0.006 | 0.039 | 0.660 | -0.599 |
| LIPID-N-1312 | PS(18:1_20:2) | GP | PS | 1.452 | 0.006 | 0.039 | 0.657 | -0.605 |
| LIPID-N-1286 | PS(15:0_18:2) | GP | PS | 1.462 | 0.008 | 0.048 | 0.657 | -0.605 |
| LIPID-N-0569 | PS(18:0_17:1) | GP | PS | 1.504 | 0.004 | 0.034 | 0.652 | -0.617 |
| LIPID-N-1114 | PG(17:0_18:0) | GP | PG | 1.647 | 0.001 | 0.018 | 0.650 | -0.620 |
| LIPID-N-0373 | PE(20:1_18:2) | GP | PE | 1.479 | 0.008 | 0.047 | 0.648 | -0.626 |
| LIPID-N-0234 | PC(17:0_18:2) | GP | PC | 1.453 | 0.008 | 0.047 | 0.645 | -0.633 |
| LIPID-P-1322 | PE(P-20:0_18:1) | GP | PE-P | 1.398 | 0.013 | 0.061 | 0.645 | -0.633 |
| LIPID-N-1135 | PG(18:0_16:1) | GP | PG | 1.611 | 0.002 | 0.024 | 0.640 | -0.643 |
| LIPID-N-0782 | PE(O-22:1_18:2) | GP | PE-O | 1.437 | 0.008 | 0.046 | 0.639 | -0.645 |
| LIPID-P-0392 | LPC(22:6) | GP | LPC | 2.047 | 0.000 | 0.002 | 0.638 | -0.647 |
| LIPID-P-0160 | CoenzymeQ9 | PR | CoQ | 1.757 | 0.001 | 0.015 | 0.635 | -0.655 |
| LIPID-N-0774 | PE(O-18:2_18:1) | GP | PE-O | 1.898 | 0.000 | 0.008 | 0.634 | -0.658 |
| LIPID-N-0292 | PC(20:5_18:0) | GP | PC | 1.415 | 0.008 | 0.049 | 0.633 | -0.659 |
| LIPID-N-1380 | PI(16:0_19:0) | GP | PI | 1.601 | 0.003 | 0.026 | 0.630 | -0.666 |
| LIPID-N-0314 | PC(18:2_20:5) | GP | PC | 1.795 | 0.000 | 0.010 | 0.629 | -0.668 |
| LIPID-P-0661 | PE(P-16:0_22:6) | GP | PE-P | 1.349 | 0.016 | 0.068 | 0.629 | -0.668 |
| LIPID-N-1023 | PC(15:1_19:1) | GP | PC | 1.460 | 0.008 | 0.047 | 0.629 | -0.669 |
| LIPID-P-2179 | HexCer(t28:1/18:1(2OH)) | SP | HexCer-AP | 1.352 | 0.020 | 0.079 | 0.629 | -0.669 |
| LIPID-P-1387 | PE(P-18:1_20:5) | GP | PE-P | 1.343 | 0.018 | 0.075 | 0.628 | -0.670 |
| LIPID-N-0418 | PE(19:0_22:6) | GP | PE | 1.613 | 0.002 | 0.024 | 0.625 | -0.678 |
| LIPID-N-0490 | PI(18:2_16:0) | GP | PI | 1.510 | 0.006 | 0.038 | 0.618 | -0.693 |
| LIPID-N-0859 | PE(O-18:1_22:6) | GP | PE-O | 1.757 | 0.000 | 0.010 | 0.618 | -0.694 |
| LIPID-N-0581 | PS(18:2_18:1) | GP | PS | 1.293 | 0.023 | 0.086 | 0.617 | -0.696 |
| LIPID-N-0863 | PE(O-19:1_22:6) | GP | PE-O | 1.284 | 0.014 | 0.061 | 0.617 | -0.697 |
| LIPID-N-0254 | PC(18:1_18:2) | GP | PC | 1.694 | 0.001 | 0.018 | 0.617 | -0.697 |
| LIPID-N-0287 | PC(18:2_18:3) | GP | PC | 1.364 | 0.014 | 0.062 | 0.613 | -0.705 |
| LIPID-N-1140 | PG(16:0_22:1) | GP | PG | 1.626 | 0.002 | 0.023 | 0.612 | -0.709 |
| LIPID-N-1116 | PG(17:0_19:0) | GP | PG | 1.722 | 0.001 | 0.017 | 0.604 | -0.727 |
| LIPID-N-1410 | PI(10:0_26:2) | GP | PI | 1.549 | 0.004 | 0.034 | 0.601 | -0.735 |
| LIPID-N-0190 | LPS(24:0) | GP | LPS | 1.337 | 0.025 | 0.092 | 0.599 | -0.739 |
| LIPID-N-0855 | PE(O-18:3_20:4) | GP | PE-O | 1.503 | 0.006 | 0.039 | 0.597 | -0.743 |
| LIPID-N-1192 | PG(18:2_21:2) | GP | PG | 1.709 | 0.001 | 0.016 | 0.597 | -0.744 |
| LIPID-N-1145 | PG(19:0_20:1) | GP | PG | 1.510 | 0.004 | 0.033 | 0.596 | -0.747 |
| LIPID-P-2531 | Cer(d18:2/22:0(2OH)) | SP | Cer-AS | 1.297 | 0.021 | 0.080 | 0.596 | -0.747 |
| LIPID-N-1381 | PI(17:0_18:0) | GP | PI | 1.493 | 0.005 | 0.038 | 0.593 | -0.753 |
| LIPID-N-0528 | PI(19:0_20:4) | GP | PI | 1.549 | 0.005 | 0.037 | 0.591 | -0.759 |
| LIPID-N-1358 | PS(17:1_22:6) | GP | PS | 1.323 | 0.014 | 0.061 | 0.591 | -0.760 |
| LIPID-P-1386 | PE(P-14:0_22:6) | GP | PE-P | 1.389 | 0.014 | 0.061 | 0.588 | -0.767 |
| LIPID-P-1331 | PE(P-18:0_18:2) | GP | PE-P | 1.417 | 0.010 | 0.052 | 0.588 | -0.767 |
| LIPID-N-0353 | PE(18:0_18:2) | GP | PE | 1.749 | 0.001 | 0.013 | 0.584 | -0.775 |
| LIPID-P-0390 | LPC(0:0/22:5) | GP | LPC | 2.126 | 0.000 | 0.010 | 0.583 | -0.778 |
| LIPID-N-0236 | PC(18:0_18:2) | GP | PC | 1.702 | 0.001 | 0.017 | 0.583 | -0.779 |
| LIPID-P-0559 | PC(O-16:1_20:4) | GP | PC-O | 1.712 | 0.001 | 0.018 | 0.582 | -0.781 |
| LIPID-N-1134 | PG(19:0_15:1) | GP | PG | 1.630 | 0.002 | 0.023 | 0.581 | -0.784 |
| LIPID-N-1085 | PE(18:2_18:3) | GP | PE | 1.423 | 0.011 | 0.055 | 0.576 | -0.796 |
| LIPID-P-0658 | PE(P-18:1_20:4) | GP | PE-P | 1.486 | 0.007 | 0.041 | 0.571 | -0.809 |
| LIPID-P-1375 | PE(P-18:0_20:5) | GP | PE-P | 1.283 | 0.022 | 0.083 | 0.569 | -0.814 |
| LIPID-N-0881 | PMeOH(16:0_18:2) | GP | PMeOH | 1.614 | 0.002 | 0.023 | 0.569 | -0.814 |
| LIPID-P-0115 | Cer(d18:1/22:0) | SP | Cer-NS | 1.303 | 0.040 | 0.123 | 0.568 | -0.815 |
| LIPID-N-0308 | PC(18:0_22:6) | GP | PC | 1.733 | 0.001 | 0.015 | 0.568 | -0.816 |
| LIPID-N-0257 | PC(19:1_18:2) | GP | PC | 1.798 | 0.000 | 0.010 | 0.567 | -0.819 |
| LIPID-N-1275 | PS(18:0_21:1) | GP | PS | 1.624 | 0.002 | 0.023 | 0.566 | -0.821 |
| LIPID-N-0961 | LNAPE(22:6/N-20:4) | GP | LNAPE | 1.215 | 0.040 | 0.123 | 0.566 | -0.822 |
| LIPID-N-0834 | PE(O-17:1_20:5) | GP | PE-O | 1.635 | 0.002 | 0.023 | 0.564 | -0.826 |
| LIPID-N-0270 | PC(18:2_18:2) | GP | PC | 1.606 | 0.003 | 0.027 | 0.564 | -0.827 |
| LIPID-N-0549 | PI(18:1_20:5) | GP | PI | 1.394 | 0.010 | 0.051 | 0.563 | -0.830 |
| LIPID-P-0161 | CoenzymeQ10 | PR | CoQ | 1.780 | 0.000 | 0.012 | 0.562 | -0.832 |
| LIPID-N-1063 | PE(17:0_18:2) | GP | PE | 1.622 | 0.002 | 0.022 | 0.559 | -0.840 |
| LIPID-N-0494 | PI(17:0_18:2) | GP | PI | 1.859 | 0.000 | 0.008 | 0.558 | -0.841 |
| LIPID-P-0662 | PE(P-18:0_22:6) | GP | PE-P | 1.675 | 0.001 | 0.018 | 0.557 | -0.845 |
| LIPID-N-1330 | PS(17:1_20:4) | GP | PS | 1.553 | 0.004 | 0.033 | 0.556 | -0.846 |
| LIPID-N-0590 | PS(19:0_20:4) | GP | PS | 1.686 | 0.001 | 0.018 | 0.556 | -0.848 |
| LIPID-N-1041 | PC(18:2_19:2) | GP | PC | 1.631 | 0.002 | 0.023 | 0.553 | -0.855 |
| LIPID-P-0539 | PC(O-18:0_18:1) | GP | PC-O | 1.909 | 0.000 | 0.007 | 0.551 | -0.860 |
| LIPID-N-0589 | PS(18:0_20:4) | GP | PS | 1.678 | 0.001 | 0.019 | 0.550 | -0.863 |
| LIPID-N-0836 | PE(O-17:2_20:4) | GP | PE-O | 1.803 | 0.000 | 0.010 | 0.550 | -0.864 |
| LIPID-N-0382 | PE(18:2_18:2) | GP | PE | 1.457 | 0.009 | 0.049 | 0.549 | -0.864 |
| LIPID-N-0508 | PI(18:1_18:2) | GP | PI | 1.611 | 0.003 | 0.026 | 0.549 | -0.864 |
| LIPID-P-1323 | PE(P-18:0_20:1) | GP | PE-P | 1.570 | 0.003 | 0.030 | 0.549 | -0.864 |
| LIPID-N-0778 | PE(O-18:2_20:1) | GP | PE-O | 1.843 | 0.000 | 0.010 | 0.548 | -0.868 |
| LIPID-N-1173 | PG(16:0_18:3) | GP | PG | 1.772 | 0.000 | 0.010 | 0.547 | -0.872 |
| LIPID-P-0564 | PC(O-16:1_20:5) | GP | PC-O | 1.733 | 0.001 | 0.015 | 0.541 | -0.888 |
| LIPID-N-1193 | PG(19:0_20:4) | GP | PG | 1.787 | 0.000 | 0.012 | 0.540 | -0.889 |
| LIPID-N-0965 | LNAPE(18:2/N-18:2) | GP | LNAPE | 1.338 | 0.017 | 0.072 | 0.539 | -0.892 |
| LIPID-N-1404 | PI(14:1_18:1) | GP | PI | 1.680 | 0.001 | 0.018 | 0.539 | -0.893 |
| LIPID-N-1278 | PS(18:0_22:1) | GP | PS | 1.564 | 0.003 | 0.030 | 0.535 | -0.902 |
| LIPID-N-1090 | PE(20:2_18:3) | GP | PE | 1.716 | 0.001 | 0.018 | 0.535 | -0.902 |
| LIPID-N-0495 | PI(18:0_18:2) | GP | PI | 1.778 | 0.001 | 0.013 | 0.532 | -0.910 |
| LIPID-P-2230 | HexCer(d18:1/25:1) | SP | HexCer-NS | 1.672 | 0.001 | 0.018 | 0.532 | -0.911 |
| LIPID-N-1068 | PE(18:1_17:2) | GP | PE | 1.863 | 0.001 | 0.015 | 0.531 | -0.912 |
| LIPID-N-0873 | PE(O-20:2_22:6) | GP | PE-O | 2.079 | 0.000 | 0.010 | 0.529 | -0.918 |
| LIPID-N-0447 | PG(18:0_18:2) | GP | PG | 1.784 | 0.000 | 0.010 | 0.529 | -0.919 |
| LIPID-P-1407 | PE(P-20:2_22:6) | GP | PE-P | 1.717 | 0.001 | 0.018 | 0.528 | -0.923 |
| LIPID-N-1383 | PI(18:0_19:0) | GP | PI | 1.715 | 0.001 | 0.015 | 0.527 | -0.923 |
| LIPID-N-0430 | PE(22:6_18:2) | GP | PE | 1.922 | 0.000 | 0.007 | 0.527 | -0.923 |
| LIPID-N-1189 | PG(18:1_18:3) | GP | PG | 1.897 | 0.000 | 0.008 | 0.527 | -0.923 |
| LIPID-N-0791 | PE(O-18:2_18:2) | GP | PE-O | 1.691 | 0.001 | 0.018 | 0.524 | -0.933 |
| LIPID-P-0664 | PE(P-18:1_22:6) | GP | PE-P | 1.795 | 0.000 | 0.010 | 0.524 | -0.933 |
| LIPID-N-0252 | PC(17:1_18:2) | GP | PC | 1.638 | 0.002 | 0.023 | 0.522 | -0.937 |
| LIPID-N-0195 | LPS(24:1) | GP | LPS | 1.284 | 0.033 | 0.109 | 0.521 | -0.942 |
| LIPID-N-1342 | PS(18:2_20:4) | GP | PS | 1.442 | 0.008 | 0.048 | 0.518 | -0.949 |
| LIPID-N-1411 | PI(19:0_17:2) | GP | PI | 1.810 | 0.000 | 0.010 | 0.518 | -0.950 |
| LIPID-N-1021 | PC(15:1_18:2) | GP | PC | 1.820 | 0.000 | 0.010 | 0.515 | -0.958 |
| LIPID-P-1339 | PE(P-18:2_16:1) | GP | PE-P | 1.478 | 0.006 | 0.038 | 0.513 | -0.962 |
| LIPID-P-1362 | PE(P-19:0_20:4) | GP | PE-P | 1.547 | 0.004 | 0.033 | 0.512 | -0.966 |
| LIPID-P-1333 | PE(P-20:0_18:2) | GP | PE-P | 1.634 | 0.001 | 0.020 | 0.509 | -0.974 |
| LIPID-N-0355 | PE(19:0_18:2) | GP | PE | 1.824 | 0.000 | 0.010 | 0.509 | -0.974 |
| LIPID-P-2199 | HexCer(d16:1/26:1) | SP | HexCer-NS | 1.240 | 0.006 | 0.038 | 0.507 | -0.979 |
| LIPID-N-0869 | PE(O-18:3_20:5) | GP | PE-O | 1.502 | 0.006 | 0.038 | 0.505 | -0.986 |
| LIPID-P-2198 | HexCer(d16:1/26:0) | SP | HexCer-NS | 1.107 | 0.009 | 0.049 | 0.504 | -0.990 |
| LIPID-N-0466 | PG(18:2_22:6) | GP | PG | 1.674 | 0.000 | 0.010 | 0.501 | -0.998 |
| LIPID-N-1147 | PG(18:0_22:1) | GP | PG | 1.881 | 0.000 | 0.008 | 0.491 | -1.027 |
| LIPID-N-0551 | PI(18:0_22:6) | GP | PI | 2.064 | 0.000 | 0.002 | 0.479 | -1.062 |
| LIPID-P-1396 | PE(P-18:2_20:5) | GP | PE-P | 1.409 | 0.011 | 0.055 | 0.477 | -1.069 |
| LIPID-N-0790 | PE(O-18:3_18:1) | GP | PE-O | 1.953 | 0.000 | 0.006 | 0.474 | -1.076 |
| LIPID-N-0167 | LPG(18:0) | GP | LPG | 1.072 | 0.009 | 0.049 | 0.473 | -1.081 |
| LIPID-P-0647 | PE(P-18:1_18:2) | GP | PE-P | 1.504 | 0.006 | 0.038 | 0.472 | -1.084 |
| LIPID-P-0565 | PC(O-18:2_20:4) | GP | PC-O | 1.911 | 0.000 | 0.006 | 0.467 | -1.099 |
| LIPID-N-1336 | PS(20:1_20:4) | GP | PS | 1.840 | 0.000 | 0.010 | 0.464 | -1.107 |
| LIPID-P-1389 | PE(P-17:0_22:6) | GP | PE-P | 1.770 | 0.000 | 0.010 | 0.463 | -1.110 |
| LIPID-N-1011 | PC(15:1_17:1) | GP | PC | 1.650 | 0.001 | 0.019 | 0.457 | -1.129 |
| LIPID-P-2231 | HexCer(d18:1/26:2) | SP | HexCer-NS | 1.540 | 0.001 | 0.018 | 0.456 | -1.133 |
| LIPID-N-0556 | PI(18:1_22:6) | GP | PI | 2.000 | 0.000 | 0.003 | 0.451 | -1.148 |
| LIPID-N-1075 | PE(16:2_18:2) | GP | PE | 1.636 | 0.002 | 0.022 | 0.451 | -1.148 |
| LIPID-N-1271 | PS(17:0_21:1) | GP | PS | 1.698 | 0.001 | 0.017 | 0.450 | -1.152 |
| LIPID-P-1353 | PE(P-18:2_18:2) | GP | PE-P | 1.586 | 0.002 | 0.025 | 0.448 | -1.158 |
| LIPID-N-0370 | PE(18:2_19:1) | GP | PE | 1.467 | 0.008 | 0.047 | 0.447 | -1.161 |
| LIPID-P-1388 | PE(P-18:2_20:4) | GP | PE-P | 1.652 | 0.002 | 0.021 | 0.443 | -1.174 |
| LIPID-N-0657 | LPA(18:4) | GP | LPA | 1.292 | 0.021 | 0.079 | 0.443 | -1.174 |
| LIPID-N-0975 | LNAPE(22:6/N-18:2) | GP | LNAPE | 1.903 | 0.000 | 0.007 | 0.439 | -1.187 |
| LIPID-N-0548 | PI(16:0_22:6) | GP | PI | 1.857 | 0.000 | 0.010 | 0.436 | -1.197 |
| LIPID-N-0237 | PC(19:0_18:2) | GP | PC | 1.855 | 0.000 | 0.010 | 0.436 | -1.199 |
| LIPID-P-1397 | PE(P-18:3_20:4) | GP | PE-P | 1.552 | 0.004 | 0.032 | 0.419 | -1.256 |
| LIPID-N-0808 | PE(O-18:3_18:2) | GP | PE-O | 1.697 | 0.001 | 0.018 | 0.415 | -1.268 |
| LIPID-P-2241 | HexCer(d18:2/25:0) | SP | HexCer-NS | 1.667 | 0.000 | 0.010 | 0.413 | -1.274 |
| LIPID-N-1143 | PG(17:0_22:1) | GP | PG | 1.895 | 0.000 | 0.007 | 0.409 | -1.291 |
| LIPID-P-1400 | PE(P-18:3_22:4) | GP | PE-P | 1.637 | 0.002 | 0.023 | 0.397 | -1.334 |
| LIPID-N-1207 | PG(21:1_20:4) | GP | PG | 2.006 | 0.000 | 0.003 | 0.394 | -1.342 |
| LIPID-N-0453 | PG(18:2_18:2) | GP | PG | 1.851 | 0.000 | 0.010 | 0.392 | -1.352 |
| LIPID-P-0663 | PE(P-20:0_22:6) | GP | PE-P | 1.789 | 0.000 | 0.010 | 0.371 | -1.431 |
| LIPID-P-2082 | Cer(d18:2/19:0) | SP | Cer-NS | 1.624 | 0.006 | 0.038 | 0.359 | -1.478 |
| LIPID-P-0568 | PC(O-16:1_22:6) | GP | PC-O | 1.799 | 0.000 | 0.010 | 0.358 | -1.480 |
| LIPID-N-1159 | PG(18:0_19:2) | GP | PG | 1.797 | 0.000 | 0.010 | 0.348 | -1.523 |
| LIPID-P-1405 | PE(P-18:2_22:6) | GP | PE-P | 1.798 | 0.000 | 0.010 | 0.340 | -1.557 |
| LIPID-N-0105 | FFA(24:6) | FA | FFA | 1.076 | 0.014 | 0.061 | 0.321 | -1.638 |

| **Supplemental Table S6** | | |  | |  | |  | |  | |  | |  | |  | |  | |  | |  | |  | |
| --- | --- | --- | --- | --- | --- | --- | --- | --- | --- | --- | --- | --- | --- | --- | --- | --- | --- | --- | --- | --- | --- | --- | --- | --- |
| **Differentially Expressed Lipids Common to both 1 and 5 ppm PFOA vs. Control KC mice** | | | | | | | | | | | | | | | | |  | |  | |  | |  | |
|  |  |  | |  | |  | |  | |  | |  | |  | |  | |  | |  | |  | |  |
|  |  | **Class** | | **Class** | | **Control** | | | | **P1** | | | | **P5** | | | |  | |  | |  | |  |
| **Index** | **Compounds** | **I** | | **II** | | **AVE** | | **SD** | | **AVE** | | **SD** | | **AVE** | | **SD** | |  | |  | |  | |  |
| LIPID-N-0041 | 11(S)-HETE | FA | | Eicosanoid | | 0.246 | | 0.073 | | 0.507 | | 0.214 | | 0.395 | | 0.152 | | Up | |  | |  | |  |
| LIPID-P-0013 | Carnitine C10:0 | FA | | CAR | | 0.009 | | 0.003 | | 0.021 | | 0.008 | | 0.014 | | 0.006 | | Up | |  | |  | |  |
| LIPID-P-0014 | Carnitine C12:0 | FA | | CAR | | 0.015 | | 0.005 | | 0.034 | | 0.013 | | 0.028 | | 0.008 | | Up | |  | |  | |  |
| LIPID-P-0036 | Carnitine C12:1-OH | FA | | CAR | | 0.010 | | 0.003 | | 0.018 | | 0.008 | | 0.016 | | 0.005 | | Up | |  | |  | |  |
| LIPID-P-0015 | Carnitine C13:0 | FA | | CAR | | 0.004 | | 0.001 | | 0.008 | | 0.002 | | 0.007 | | 0.002 | | Up | |  | |  | |  |
| LIPID-P-0017 | Carnitine C14:0 | FA | | CAR | | 0.270 | | 0.103 | | 0.567 | | 0.206 | | 0.568 | | 0.149 | | Up | |  | |  | |  |
| LIPID-P-0037 | Carnitine C14:1 | FA | | CAR | | 0.033 | | 0.012 | | 0.088 | | 0.041 | | 0.059 | | 0.024 | | Up | |  | |  | |  |
| LIPID-P-0038 | Carnitine C14:1-OH | FA | | CAR | | 0.023 | | 0.008 | | 0.058 | | 0.031 | | 0.047 | | 0.019 | | Up | |  | |  | |  |
| LIPID-P-0020 | Carnitine C14-OH | FA | | CAR | | 0.047 | | 0.013 | | 0.098 | | 0.040 | | 0.092 | | 0.028 | | Up | |  | |  | |  |
| LIPID-P-0018 | Carnitine C15:0 | FA | | CAR | | 0.044 | | 0.013 | | 0.108 | | 0.026 | | 0.108 | | 0.047 | | Up | |  | |  | |  |
| LIPID-P-0021 | Carnitine C16:0 | FA | | CAR | | 3.778 | | 1.065 | | 6.954 | | 1.282 | | 7.630 | | 2.297 | | Up | |  | |  | |  |
| LIPID-P-0039 | Carnitine C16:1 | FA | | CAR | | 0.064 | | 0.030 | | 0.157 | | 0.046 | | 0.163 | | 0.075 | | Up | |  | |  | |  |
| LIPID-P-0040 | Carnitine C16:1-OH | FA | | CAR | | 0.041 | | 0.014 | | 0.114 | | 0.054 | | 0.110 | | 0.050 | | Up | |  | |  | |  |
| LIPID-P-1298 | Carnitine C16:3 | FA | | CAR | | 0.002 | | 0.001 | | 0.006 | | 0.003 | | 0.004 | | 0.002 | | Up | |  | |  | |  |
| LIPID-P-0022 | Carnitine C16-OH | FA | | CAR | | 0.211 | | 0.046 | | 0.527 | | 0.221 | | 0.469 | | 0.191 | | Up | |  | |  | |  |
| LIPID-P-1282 | Carnitine C17:0 | FA | | CAR | | 0.054 | | 0.018 | | 0.145 | | 0.040 | | 0.118 | | 0.058 | | Up | |  | |  | |  |
| LIPID-P-1290 | Carnitine C17:1 | FA | | CAR | | 0.001 | | 0.002 | | 0.006 | | 0.002 | | 0.005 | | 0.002 | | Up | |  | |  | |  |
| LIPID-P-0042 | Carnitine C18:1 | FA | | CAR | | 0.607 | | 0.231 | | 1.556 | | 0.403 | | 1.512 | | 0.700 | | Up | |  | |  | |  |
| LIPID-P-0045 | Carnitine C18:1-OH | FA | | CAR | | 0.105 | | 0.035 | | 0.371 | | 0.185 | | 0.277 | | 0.154 | | Up | |  | |  | |  |
| LIPID-P-0051 | Carnitine C18:2-OH | FA | | CAR | | 0.050 | | 0.012 | | 0.168 | | 0.079 | | 0.124 | | 0.061 | | Up | |  | |  | |  |
| LIPID-P-0025 | Carnitine C18-OH | FA | | CAR | | 0.098 | | 0.031 | | 0.189 | | 0.059 | | 0.157 | | 0.058 | | Up | |  | |  | |  |
| LIPID-P-0024 | Carnitine C20:0 | FA | | CAR | | 0.050 | | 0.016 | | 0.082 | | 0.025 | | 0.068 | | 0.015 | | Up | |  | |  | |  |
| LIPID-P-0043 | Carnitine C20:1 | FA | | CAR | | 0.004 | | 0.002 | | 0.009 | | 0.002 | | 0.008 | | 0.003 | | Up | |  | |  | |  |
| LIPID-P-0050 | Carnitine C20:2 | FA | | CAR | | 0.142 | | 0.058 | | 0.319 | | 0.067 | | 0.295 | | 0.103 | | Up | |  | |  | |  |
| LIPID-P-1300 | Carnitine C20:3 | FA | | CAR | | 0.014 | | 0.002 | | 0.029 | | 0.009 | | 0.036 | | 0.014 | | Up | |  | |  | |  |
| LIPID-P-1302 | Carnitine C20:4 | FA | | CAR | | 0.143 | | 0.022 | | 0.244 | | 0.083 | | 0.238 | | 0.074 | | Up | |  | |  | |  |
| LIPID-P-1297 | Carnitine C22:2 | FA | | CAR | | 0.023 | | 0.009 | | 0.044 | | 0.011 | | 0.039 | | 0.011 | | Up | |  | |  | |  |
| LIPID-P-1301 | Carnitine C22:3 | FA | | CAR | | 0.003 | | 0.002 | | 0.009 | | 0.004 | | 0.010 | | 0.005 | | Up | |  | |  | |  |
| LIPID-P-1303 | Carnitine C22:4 | FA | | CAR | | 0.010 | | 0.002 | | 0.028 | | 0.012 | | 0.029 | | 0.013 | | Up | |  | |  | |  |
| LIPID-P-1305 | Carnitine C22:5 | FA | | CAR | | 0.001 | | 0.001 | | 0.003 | | 0.001 | | 0.003 | | 0.002 | | Up | |  | |  | |  |
| LIPID-P-0115 | Cer(d18:1/22:0) | SP | | Cer-NS | | 31.191 | | 14.184 | | 12.142 | | 6.025 | | 17.725 | | 5.397 | | Down | |  | |  | |  |
| LIPID-P-2099 | Cer(d19:2/24:0) | SP | | Cer-NS | | 35.669 | | 13.777 | | 90.149 | | 53.130 | | 98.806 | | 62.605 | | Up | |  | |  | |  |
| LIPID-P-2552 | Cer(d20:1/22:0(2OH)) | SP | | Cer-AS | | 16.237 | | 3.746 | | 55.001 | | 32.693 | | 39.791 | | 19.735 | | Up | |  | |  | |  |
| LIPID-P-2553 | Cer(d20:1/23:0(2OH)) | SP | | Cer-AS | | 11.079 | | 2.738 | | 34.987 | | 23.795 | | 25.162 | | 12.649 | | Up | |  | |  | |  |
| LIPID-P-2102 | Cer(d20:1/23:1) | SP | | Cer-NS | | 31.762 | | 18.172 | | 69.957 | | 38.951 | | 83.202 | | 38.789 | | Up | |  | |  | |  |
| LIPID-P-2555 | Cer(d20:1/24:0(2OH)) | SP | | Cer-AS | | 43.643 | | 14.838 | | 173.591 | | 103.927 | | 128.492 | | 68.662 | | Up | |  | |  | |  |
| LIPID-P-2556 | Cer(d20:1/24:1(2OH)) | SP | | Cer-AS | | 2.640 | | 0.993 | | 11.022 | | 6.812 | | 9.842 | | 5.581 | | Up | |  | |  | |  |
| LIPID-P-2557 | Cer(d20:1/24:2(2OH)) | SP | | Cer-AS | | 5.939 | | 3.403 | | 19.029 | | 14.947 | | 18.963 | | 14.629 | | Up | |  | |  | |  |
| LIPID-P-2113 | Cer(d20:2/24:0) | SP | | Cer-NS | | 178.362 | | 54.018 | | 516.852 | | 316.041 | | 600.891 | | 402.987 | | Up | |  | |  | |  |
| LIPID-P-2422 | Cer(t17:2/16:0) | SP | | Cer-NP | | 86.879 | | 29.157 | | 272.593 | | 176.122 | | 193.167 | | 84.901 | | Up | |  | |  | |  |
| LIPID-P-2423 | Cer(t17:2/16:1) | SP | | Cer-NP | | 8.446 | | 3.954 | | 24.218 | | 13.746 | | 19.551 | | 12.405 | | Up | |  | |  | |  |
| LIPID-P-2433 | Cer(t17:2/24:1) | SP | | Cer-NP | | 150.621 | | 44.291 | | 551.242 | | 359.710 | | 345.123 | | 142.011 | | Up | |  | |  | |  |
| LIPID-P-2442 | Cer(t18:0/16:0) | SP | | Cer-NP | | 1.938 | | 0.893 | | 5.386 | | 3.377 | | 3.725 | | 1.588 | | Up | |  | |  | |  |
| LIPID-P-1513 | Cer(t18:0/24:0(2OH)) | SP | | Cer-AP | | 1.995 | | 0.404 | | 4.580 | | 2.717 | | 3.859 | | 2.342 | | Up | |  | |  | |  |
| LIPID-P-2453 | Cer(t18:1/25:0) | SP | | Cer-NP | | 2.426 | | 0.524 | | 8.171 | | 4.985 | | 7.125 | | 4.098 | | Up | |  | |  | |  |
| LIPID-N-0609 | CerP(d18:1/18:1) | SP | | CerP | | 293.560 | | 55.998 | | 427.528 | | 87.867 | | 475.308 | | 129.107 | | Up | |  | |  | |  |
| LIPID-P-0161 | CoenzymeQ10 | PR | | CoQ | | 27.853 | | 6.414 | | 15.288 | | 3.964 | | 15.649 | | 3.458 | | Down | |  | |  | |  |
| LIPID-P-0160 | CoenzymeQ9 | PR | | CoQ | | 115.815 | | 20.359 | | 87.595 | | 23.267 | | 73.557 | | 15.980 | | Down | |  | |  | |  |
| LIPID-N-0964 | LNAPE(18:1/N-18:2) | GP | | LNAPE | | 140.899 | | 19.675 | | 67.384 | | 37.019 | | 95.404 | | 44.484 | | Down | |  | |  | |  |
| LIPID-N-0965 | LNAPE(18:2/N-18:2) | GP | | LNAPE | | 30.783 | | 8.557 | | 11.712 | | 8.540 | | 16.586 | | 11.175 | | Down | |  | |  | |  |
| LIPID-N-0968 | LNAPE(20:4/N-18:2) | GP | | LNAPE | | 6.074 | | 0.972 | | 3.753 | | 2.128 | | 4.317 | | 1.391 | | Down | |  | |  | |  |
| LIPID-N-0975 | LNAPE(22:6/N-18:2) | GP | | LNAPE | | 0.725 | | 0.174 | | 0.401 | | 0.169 | | 0.319 | | 0.080 | | Down | |  | |  | |  |
| LIPID-N-0657 | LPA(18:4) | GP | | LPA | | 0.569 | | 0.316 | | 0.196 | | 0.113 | | 0.252 | | 0.149 | | Down | |  | |  | |  |
| LIPID-P-0378 | LPC(0:0/18:2) | GP | | LPC | | 7.625 | | 1.230 | | 4.301 | | 1.646 | | 5.674 | | 2.079 | | Down | |  | |  | |  |
| LIPID-P-0390 | LPC(0:0/22:5) | GP | | LPC | | 1.769 | | 0.114 | | 1.217 | | 0.200 | | 1.032 | | 0.146 | | Down | |  | |  | |  |
| LIPID-P-1929 | LPC(16:2) | GP | | LPC | | 2.048 | | 0.313 | | 2.708 | | 0.403 | | 3.692 | | 1.397 | | Up | |  | |  | |  |
| LIPID-P-1931 | LPC(16:3) | GP | | LPC | | 0.157 | | 0.152 | | 0.561 | | 0.154 | | 0.629 | | 0.381 | | Up | |  | |  | |  |
| LIPID-P-0379 | LPC(18:2/0:0) | GP | | LPC | | 8.078 | | 1.252 | | 4.516 | | 1.731 | | 5.956 | | 2.285 | | Down | |  | |  | |  |
| LIPID-P-0389 | LPC(20:5) | GP | | LPC | | 35.405 | | 8.683 | | 19.372 | | 11.900 | | 23.876 | | 7.278 | | Down | |  | |  | |  |
| LIPID-P-0392 | LPC(22:6) | GP | | LPC | | 55.972 | | 4.813 | | 41.829 | | 4.459 | | 35.733 | | 5.019 | | Down | |  | |  | |  |
| LIPID-P-1930 | LPC(24:2) | GP | | LPC | | 0.158 | | 0.031 | | 0.303 | | 0.092 | | 0.298 | | 0.143 | | Up | |  | |  | |  |
| LIPID-P-0365 | LPC(26:0) | GP | | LPC | | 0.040 | | 0.019 | | 0.115 | | 0.040 | | 0.125 | | 0.102 | | Up | |  | |  | |  |
| LIPID-P-0393 | LPC(O-14:0) | GP | | LPC-O | | 0.062 | | 0.026 | | 0.134 | | 0.033 | | 0.143 | | 0.084 | | Up | |  | |  | |  |
| LIPID-P-0394 | LPC(O-16:0) | GP | | LPC-O | | 3.279 | | 0.967 | | 7.112 | | 2.101 | | 6.766 | | 2.604 | | Up | |  | |  | |  |
| LIPID-P-0406 | LPC(O-20:3) | GP | | LPC-O | | 0.024 | | 0.005 | | 0.046 | | 0.017 | | 0.042 | | 0.020 | | Up | |  | |  | |  |
| LIPID-P-0423 | LPE(0:0/18:2) | GP | | LPE | | 626.060 | | 115.300 | | 326.593 | | 166.411 | | 424.322 | | 214.906 | | Down | |  | |  | |  |
| LIPID-P-0431 | LPE(22:3) | GP | | LPE | | 18.898 | | 4.281 | | 39.617 | | 9.694 | | 36.660 | | 18.334 | | Up | |  | |  | |  |
| LIPID-P-0433 | LPE(22:4) | GP | | LPE | | 435.230 | | 84.324 | | 934.088 | | 229.035 | | 855.186 | | 443.079 | | Up | |  | |  | |  |
| LIPID-N-0175 | LPI(16:0) | GP | | LPI | | 25.261 | | 3.173 | | 17.088 | | 8.934 | | 19.996 | | 3.830 | | Down | |  | |  | |  |
| LIPID-N-0176 | LPI(18:0) | GP | | LPI | | 160.673 | | 14.154 | | 101.703 | | 40.009 | | 110.165 | | 24.590 | | Down | |  | |  | |  |
| LIPID-N-0192 | LPS(18:1) | GP | | LPS | | 0.856 | | 0.134 | | 1.412 | | 0.407 | | 1.358 | | 0.448 | | Up | |  | |  | |  |
| LIPID-N-0196 | LPS(18:2) | GP | | LPS | | 1.191 | | 0.148 | | 0.815 | | 0.406 | | 0.855 | | 0.263 | | Down | |  | |  | |  |
| LIPID-N-0197 | LPS(20:3) | GP | | LPS | | 0.151 | | 0.034 | | 0.231 | | 0.057 | | 0.365 | | 0.103 | | Up | |  | |  | |  |
| LIPID-N-0200 | LPS(22:5) | GP | | LPS | | 0.077 | | 0.034 | | 0.164 | | 0.038 | | 0.190 | | 0.067 | | Up | |  | |  | |  |
| LIPID-N-0190 | LPS(24:0) | GP | | LPS | | 0.428 | | 0.108 | | 0.273 | | 0.143 | | 0.256 | | 0.148 | | Down | |  | |  | |  |
| LIPID-N-0195 | LPS(24:1) | GP | | LPS | | 0.309 | | 0.065 | | 0.135 | | 0.096 | | 0.161 | | 0.152 | | Down | |  | |  | |  |
| LIPID-N-0202 | PC(14:0_14:0) | GP | | PC | | 0.433 | | 0.174 | | 0.971 | | 0.461 | | 1.318 | | 0.600 | | Up | |  | |  | |  |
| LIPID-N-0214 | PC(14:0_16:1) | GP | | PC | | 1.041 | | 0.467 | | 1.956 | | 0.763 | | 2.870 | | 1.196 | | Up | |  | |  | |  |
| LIPID-N-0995 | PC(14:0_17:0) | GP | | PC | | 1.633 | | 0.471 | | 2.274 | | 0.613 | | 2.616 | | 0.862 | | Up | |  | |  | |  |
| LIPID-N-0217 | PC(14:0_18:1) | GP | | PC | | 12.307 | | 1.210 | | 14.785 | | 1.846 | | 16.216 | | 3.608 | | Up | |  | |  | |  |
| LIPID-N-1028 | PC(14:1_20:4) | GP | | PC | | 1.164 | | 0.382 | | 1.977 | | 0.509 | | 2.386 | | 0.913 | | Up | |  | |  | |  |
| LIPID-N-1011 | PC(15:1_17:1) | GP | | PC | | 0.849 | | 0.265 | | 0.532 | | 0.226 | | 0.388 | | 0.172 | | Down | |  | |  | |  |
| LIPID-N-1019 | PC(15:1_18:1) | GP | | PC | | 15.873 | | 3.399 | | 27.827 | | 11.123 | | 28.541 | | 11.552 | | Up | |  | |  | |  |
| LIPID-N-1021 | PC(15:1_18:2) | GP | | PC | | 13.818 | | 3.052 | | 8.312 | | 2.458 | | 7.113 | | 1.900 | | Down | |  | |  | |  |
| LIPID-N-1042 | PC(15:1_22:4) | GP | | PC | | 5.272 | | 1.168 | | 12.497 | | 5.659 | | 11.889 | | 4.965 | | Up | |  | |  | |  |
| LIPID-N-0203 | PC(16:0_14:0) | GP | | PC | | 20.020 | | 4.654 | | 33.468 | | 11.144 | | 38.915 | | 13.973 | | Up | |  | |  | |  |
| LIPID-N-0216 | PC(16:0_16:1) | GP | | PC | | 65.114 | | 9.869 | | 85.192 | | 19.737 | | 111.694 | | 33.844 | | Up | |  | |  | |  |
| LIPID-N-0232 | PC(16:0_18:2) | GP | | PC | | 3420.87 | | 377.73 | | 1842.14 | | 797.31 | | 2463.87 | | 867.89 | | Down | |  | |  | |  |
| LIPID-N-0303 | PC(16:0_22:6) | GP | | PC | | 58.428 | | 12.685 | | 43.839 | | 10.022 | | 42.268 | | 7.293 | | Down | |  | |  | |  |
| LIPID-N-0228 | PC(16:1_16:1) | GP | | PC | | 2.391 | | 0.765 | | 4.259 | | 1.544 | | 6.807 | | 2.498 | | Up | |  | |  | |  |
| LIPID-N-0234 | PC(17:0_18:2) | GP | | PC | | 227.503 | | 36.884 | | 110.835 | | 59.691 | | 146.733 | | 58.436 | | Down | |  | |  | |  |
| LIPID-N-0252 | PC(17:1_18:2) | GP | | PC | | 7.851 | | 2.217 | | 4.219 | | 1.924 | | 4.100 | | 1.493 | | Down | |  | |  | |  |
| LIPID-N-0236 | PC(18:0_18:2) | GP | | PC | | 1588.927 | | 335.037 | | 787.627 | | 277.470 | | 925.686 | | 261.090 | | Down | |  | |  | |  |
| LIPID-N-0235 | PC(18:1_18:1) | GP | | PC | | 216.485 | | 25.945 | | 146.807 | | 36.139 | | 180.274 | | 21.496 | | Down | |  | |  | |  |
| LIPID-N-0254 | PC(18:1_18:2) | GP | | PC | | 801.563 | | 106.555 | | 381.512 | | 166.556 | | 494.312 | | 167.920 | | Down | |  | |  | |  |
| LIPID-N-0243 | PC(18:1_24:1) | GP | | PC | | 1.103 | | 0.107 | | 1.773 | | 0.487 | | 1.865 | | 0.685 | | Up | |  | |  | |  |
| LIPID-N-0270 | PC(18:2_18:2) | GP | | PC | | 351.425 | | 80.054 | | 173.969 | | 91.721 | | 198.075 | | 80.899 | | Down | |  | |  | |  |
| LIPID-N-0287 | PC(18:2_18:3) | GP | | PC | | 8.499 | | 2.558 | | 4.480 | | 2.306 | | 5.214 | | 1.934 | | Down | |  | |  | |  |
| LIPID-N-1041 | PC(18:2_19:2) | GP | | PC | | 16.274 | | 3.990 | | 7.738 | | 4.838 | | 8.995 | | 3.382 | | Down | |  | |  | |  |
| LIPID-N-0314 | PC(18:2_20:5) | GP | | PC | | 1.660 | | 0.139 | | 0.783 | | 0.555 | | 1.045 | | 0.310 | | Down | |  | |  | |  |
| LIPID-N-0237 | PC(19:0_18:2) | GP | | PC | | 11.201 | | 2.849 | | 6.286 | | 2.033 | | 4.879 | | 1.845 | | Down | |  | |  | |  |
| LIPID-N-0257 | PC(19:1_18:2) | GP | | PC | | 42.291 | | 4.911 | | 18.848 | | 8.947 | | 23.964 | | 8.889 | | Down | |  | |  | |  |
| LIPID-N-0316 | PC(20:3_20:4) | GP | | PC | | 1.457 | | 0.415 | | 2.515 | | 0.484 | | 3.371 | | 1.116 | | Up | |  | |  | |  |
| LIPID-N-0292 | PC(20:5_18:0) | GP | | PC | | 11.054 | | 2.335 | | 5.755 | | 2.783 | | 7.001 | | 2.680 | | Down | |  | |  | |  |
| LIPID-N-0276 | PC(22:4_16:0) | GP | | PC | | 37.860 | | 4.570 | | 53.775 | | 11.264 | | 58.570 | | 16.456 | | Up | |  | |  | |  |
| LIPID-P-0531 | PC(O-16:0_14:0) | GP | | PC-O | | 2.041 | | 0.609 | | 3.950 | | 0.899 | | 4.919 | | 1.954 | | Up | |  | |  | |  |
| LIPID-P-0532 | PC(O-16:0_16:0) | GP | | PC-O | | 19.101 | | 4.345 | | 39.970 | | 13.076 | | 40.962 | | 24.163 | | Up | |  | |  | |  |
| LIPID-P-0554 | PC(O-16:0_20:4) | GP | | PC-O | | 15.215 | | 3.356 | | 30.838 | | 9.072 | | 27.837 | | 11.563 | | Up | |  | |  | |  |
| LIPID-P-0559 | PC(O-16:1_20:4) | GP | | PC-O | | 124.726 | | 20.441 | | 64.260 | | 29.465 | | 72.610 | | 26.397 | | Down | |  | |  | |  |
| LIPID-P-0564 | PC(O-16:1_20:5) | GP | | PC-O | | 5.136 | | 0.819 | | 2.298 | | 1.386 | | 2.776 | | 1.175 | | Down | |  | |  | |  |
| LIPID-P-0568 | PC(O-16:1_22:6) | GP | | PC-O | | 11.210 | | 2.920 | | 4.281 | | 4.351 | | 4.019 | | 2.614 | | Down | |  | |  | |  |
| LIPID-P-0539 | PC(O-18:0_18:1) | GP | | PC-O | | 0.925 | | 0.164 | | 0.589 | | 0.259 | | 0.510 | | 0.096 | | Down | |  | |  | |  |
| LIPID-P-0566 | PC(O-18:0_22:6) | GP | | PC-O | | 0.643 | | 0.171 | | 1.367 | | 0.461 | | 1.348 | | 0.629 | | Up | |  | |  | |  |
| LIPID-P-0550 | PC(O-18:1_18:2) | GP | | PC-O | | 0.465 | | 0.197 | | 1.498 | | 0.378 | | 2.090 | | 1.137 | | Up | |  | |  | |  |
| LIPID-P-0565 | PC(O-18:2_20:4) | GP | | PC-O | | 28.596 | | 5.297 | | 14.308 | | 6.316 | | 13.354 | | 4.076 | | Down | |  | |  | |  |
| LIPID-N-1080 | PE(15:1_22:3) | GP | | PE | | 2.297 | | 0.188 | | 3.101 | | 0.657 | | 3.212 | | 0.961 | | Up | |  | |  | |  |
| LIPID-N-1086 | PE(15:1_22:4) | GP | | PE | | 70.232 | | 5.796 | | 93.623 | | 19.729 | | 96.348 | | 26.179 | | Up | |  | |  | |  |
| LIPID-N-0389 | PE(16:0_22:4) | GP | | PE | | 21.651 | | 2.107 | | 34.767 | | 8.944 | | 42.488 | | 12.894 | | Up | |  | |  | |  |
| LIPID-N-1075 | PE(16:2_18:2) | GP | | PE | | 0.424 | | 0.114 | | 0.175 | | 0.107 | | 0.191 | | 0.113 | | Down | |  | |  | |  |
| LIPID-N-0336 | PE(17:0_18:1) | GP | | PE | | 2.896 | | 0.339 | | 2.496 | | 0.289 | | 2.383 | | 0.494 | | Down | |  | |  | |  |
| LIPID-N-1063 | PE(17:0_18:2) | GP | | PE | | 2.740 | | 0.526 | | 1.598 | | 0.597 | | 1.531 | | 0.647 | | Down | |  | |  | |  |
| LIPID-N-0353 | PE(18:0_18:2) | GP | | PE | | 613.768 | | 78.842 | | 310.883 | | 106.085 | | 358.598 | | 126.818 | | Down | |  | |  | |  |
| LIPID-N-1057 | PE(18:0_22:1) | GP | | PE | | 0.075 | | 0.013 | | 0.143 | | 0.051 | | 0.138 | | 0.047 | | Up | |  | |  | |  |
| LIPID-N-0392 | PE(18:0_22:4) | GP | | PE | | 36.011 | | 6.395 | | 93.130 | | 40.193 | | 80.876 | | 40.288 | | Up | |  | |  | |  |
| LIPID-N-0403 | PE(18:0_22:5) | GP | | PE | | 24.972 | | 4.700 | | 38.534 | | 13.433 | | 37.647 | | 9.113 | | Up | |  | |  | |  |
| LIPID-N-1068 | PE(18:1_17:2) | GP | | PE | | 2.259 | | 0.266 | | 1.162 | | 0.608 | | 1.200 | | 0.443 | | Down | |  | |  | |  |
| LIPID-N-0368 | PE(18:1_18:2) | GP | | PE | | 505.490 | | 71.600 | | 271.895 | | 127.979 | | 366.078 | | 126.175 | | Down | |  | |  | |  |
| LIPID-N-1072 | PE(18:1_20:2) | GP | | PE | | 7.874 | | 1.523 | | 4.277 | | 1.173 | | 5.839 | | 1.585 | | Down | |  | |  | |  |
| LIPID-N-0399 | PE(18:1_20:4) | GP | | PE | | 26.122 | | 4.193 | | 15.205 | | 7.189 | | 20.289 | | 5.585 | | Down | |  | |  | |  |
| LIPID-N-0362 | PE(18:1_22:1) | GP | | PE | | 0.282 | | 0.062 | | 0.408 | | 0.098 | | 0.457 | | 0.170 | | Up | |  | |  | |  |
| LIPID-N-0350 | PE(18:2_16:0) | GP | | PE | | 269.765 | | 38.259 | | 138.645 | | 71.168 | | 191.122 | | 88.039 | | Down | |  | |  | |  |
| LIPID-N-0382 | PE(18:2_18:2) | GP | | PE | | 56.625 | | 12.650 | | 22.469 | | 14.518 | | 31.106 | | 18.354 | | Down | |  | |  | |  |
| LIPID-N-1085 | PE(18:2_18:3) | GP | | PE | | 1.326 | | 0.334 | | 0.570 | | 0.294 | | 0.764 | | 0.393 | | Down | |  | |  | |  |
| LIPID-N-0370 | PE(18:2_19:1) | GP | | PE | | 0.751 | | 0.269 | | 0.326 | | 0.177 | | 0.336 | | 0.244 | | Down | |  | |  | |  |
| LIPID-N-0340 | PE(20:0_18:1) | GP | | PE | | 0.478 | | 0.087 | | 0.616 | | 0.128 | | 0.725 | | 0.229 | | Up | |  | |  | |  |
| LIPID-N-0373 | PE(20:1_18:2) | GP | | PE | | 10.676 | | 1.382 | | 4.756 | | 1.425 | | 6.917 | | 2.889 | | Down | |  | |  | |  |
| LIPID-N-1090 | PE(20:2_18:3) | GP | | PE | | 1.511 | | 0.311 | | 0.773 | | 0.432 | | 0.808 | | 0.322 | | Down | |  | |  | |  |
| LIPID-N-0343 | PE(22:0_18:1) | GP | | PE | | 0.159 | | 0.042 | | 0.291 | | 0.111 | | 0.299 | | 0.139 | | Up | |  | |  | |  |
| LIPID-N-0344 | PE(22:1_18:0) | GP | | PE | | 0.064 | | 0.013 | | 0.122 | | 0.043 | | 0.113 | | 0.036 | | Up | |  | |  | |  |
| LIPID-N-0359 | PE(22:2_18:0) | GP | | PE | | 0.136 | | 0.023 | | 0.193 | | 0.057 | | 0.224 | | 0.052 | | Up | |  | |  | |  |
| LIPID-N-1108 | PE(22:5_22:6) | GP | | PE | | 0.252 | | 0.055 | | 0.513 | | 0.228 | | 0.653 | | 0.244 | | Up | |  | |  | |  |
| LIPID-N-0413 | PE(22:6_16:0) | GP | | PE | | 89.600 | | 7.916 | | 73.402 | | 14.466 | | 63.450 | | 21.395 | | Down | |  | |  | |  |
| LIPID-N-0426 | PE(22:6_18:1) | GP | | PE | | 39.892 | | 5.685 | | 29.875 | | 9.377 | | 28.939 | | 9.046 | | Down | |  | |  | |  |
| LIPID-N-0430 | PE(22:6_18:2) | GP | | PE | | 2.562 | | 0.463 | | 1.588 | | 0.449 | | 1.351 | | 0.299 | | Down | |  | |  | |  |
| LIPID-N-0342 | PE(24:0_16:1) | GP | | PE | | 0.085 | | 0.026 | | 0.182 | | 0.074 | | 0.234 | | 0.128 | | Up | |  | |  | |  |
| LIPID-N-0345 | PE(24:0_18:1) | GP | | PE | | 0.981 | | 0.385 | | 2.095 | | 0.851 | | 2.120 | | 1.142 | | Up | |  | |  | |  |
| LIPID-N-1083 | PE(24:0_20:4) | GP | | PE | | 0.529 | | 0.123 | | 0.902 | | 0.423 | | 0.980 | | 0.462 | | Up | |  | |  | |  |
| LIPID-N-0729 | PE(O-16:0_16:0) | GP | | PE-O | | 0.150 | | 0.068 | | 0.352 | | 0.181 | | 0.413 | | 0.246 | | Up | |  | |  | |  |
| LIPID-N-0749 | PE(O-16:0_18:2) | GP | | PE-O | | 0.178 | | 0.127 | | 0.370 | | 0.142 | | 0.329 | | 0.104 | | Up | |  | |  | |  |
| LIPID-N-0732 | PE(O-16:0_20:0) | GP | | PE-O | | 0.026 | | 0.015 | | 0.063 | | 0.010 | | 0.064 | | 0.043 | | Up | |  | |  | |  |
| LIPID-N-0788 | PE(O-16:0_20:4) | GP | | PE-O | | 2.835 | | 1.934 | | 6.455 | | 3.321 | | 6.408 | | 2.923 | | Up | |  | |  | |  |
| LIPID-N-0792 | PE(O-16:0_22:4) | GP | | PE-O | | 2.065 | | 0.310 | | 4.707 | | 1.608 | | 5.037 | | 2.150 | | Up | |  | |  | |  |
| LIPID-N-0812 | PE(O-16:0_22:5) | GP | | PE-O | | 0.127 | | 0.034 | | 0.387 | | 0.284 | | 0.323 | | 0.137 | | Up | |  | |  | |  |
| LIPID-N-0755 | PE(O-16:1_20:1) | GP | | PE-O | | 0.906 | | 0.134 | | 0.583 | | 0.152 | | 0.747 | | 0.144 | | Down | |  | |  | |  |
| LIPID-N-0809 | PE(O-16:1_20:4) | GP | | PE-O | | 256.838 | | 25.214 | | 173.208 | | 34.577 | | 207.025 | | 46.314 | | Down | |  | |  | |  |
| LIPID-N-0759 | PE(O-16:1_22:1) | GP | | PE-O | | 0.081 | | 0.023 | | 0.167 | | 0.062 | | 0.212 | | 0.125 | | Up | |  | |  | |  |
| LIPID-N-0823 | PE(O-16:1_24:4) | GP | | PE-O | | 0.763 | | 0.206 | | 1.842 | | 0.650 | | 2.204 | | 0.920 | | Up | |  | |  | |  |
| LIPID-N-0751 | PE(O-17:1_18:1) | GP | | PE-O | | 0.626 | | 0.119 | | 1.228 | | 0.570 | | 1.331 | | 0.819 | | Up | |  | |  | |  |
| LIPID-N-0770 | PE(O-17:1_18:2) | GP | | PE-O | | 0.857 | | 0.210 | | 0.521 | | 0.158 | | 0.570 | | 0.239 | | Down | |  | |  | |  |
| LIPID-N-0834 | PE(O-17:1_20:5) | GP | | PE-O | | 0.408 | | 0.088 | | 0.198 | | 0.094 | | 0.230 | | 0.092 | | Down | |  | |  | |  |
| LIPID-N-0816 | PE(O-17:1_22:4) | GP | | PE-O | | 0.391 | | 0.089 | | 0.914 | | 0.411 | | 0.649 | | 0.259 | | Up | |  | |  | |  |
| LIPID-N-0836 | PE(O-17:2_20:4) | GP | | PE-O | | 2.485 | | 0.420 | | 1.263 | | 0.591 | | 1.366 | | 0.468 | | Down | |  | |  | |  |
| LIPID-N-0730 | PE(O-18:0_16:0) | GP | | PE-O | | 0.196 | | 0.065 | | 0.353 | | 0.074 | | 0.341 | | 0.114 | | Up | |  | |  | |  |
| LIPID-N-0738 | PE(O-18:0_18:1) | GP | | PE-O | | 0.246 | | 0.088 | | 0.407 | | 0.162 | | 0.399 | | 0.168 | | Up | |  | |  | |  |
| LIPID-N-0742 | PE(O-18:0_20:1) | GP | | PE-O | | 0.022 | | 0.007 | | 0.047 | | 0.020 | | 0.042 | | 0.022 | | Up | |  | |  | |  |
| LIPID-N-0793 | PE(O-18:0_20:4) | GP | | PE-O | | 3.526 | | 1.558 | | 10.069 | | 6.980 | | 7.644 | | 4.409 | | Up | |  | |  | |  |
| LIPID-N-0796 | PE(O-18:0_22:4) | GP | | PE-O | | 0.778 | | 0.155 | | 1.850 | | 0.804 | | 1.809 | | 1.114 | | Up | |  | |  | |  |
| LIPID-N-0817 | PE(O-18:0_22:5) | GP | | PE-O | | 0.045 | | 0.008 | | 0.094 | | 0.035 | | 0.120 | | 0.066 | | Up | |  | |  | |  |
| LIPID-N-0773 | PE(O-18:1_18:2) | GP | | PE-O | | 11.238 | | 1.124 | | 8.447 | | 2.076 | | 8.481 | | 1.870 | | Down | |  | |  | |  |
| LIPID-N-0758 | PE(O-18:1_20:1) | GP | | PE-O | | 0.272 | | 0.049 | | 0.205 | | 0.053 | | 0.208 | | 0.034 | | Down | |  | |  | |  |
| LIPID-N-0794 | PE(O-18:1_20:3) | GP | | PE-O | | 3.724 | | 0.471 | | 2.952 | | 0.283 | | 2.973 | | 0.532 | | Down | |  | |  | |  |
| LIPID-N-0814 | PE(O-18:1_20:4) | GP | | PE-O | | 125.682 | | 13.479 | | 100.968 | | 10.164 | | 102.204 | | 15.771 | | Down | |  | |  | |  |
| LIPID-N-0763 | PE(O-18:1_22:1) | GP | | PE-O | | 0.017 | | 0.004 | | 0.039 | | 0.017 | | 0.045 | | 0.025 | | Up | |  | |  | |  |
| LIPID-N-0783 | PE(O-18:1_22:2) | GP | | PE-O | | 0.034 | | 0.016 | | 0.057 | | 0.022 | | 0.059 | | 0.018 | | Up | |  | |  | |  |
| LIPID-N-0820 | PE(O-18:1_22:4) | GP | | PE-O | | 5.213 | | 1.205 | | 9.040 | | 3.647 | | 8.749 | | 3.092 | | Up | |  | |  | |  |
| LIPID-N-0774 | PE(O-18:2_18:1) | GP | | PE-O | | 14.609 | | 2.013 | | 8.504 | | 2.633 | | 9.257 | | 1.604 | | Down | |  | |  | |  |
| LIPID-N-0791 | PE(O-18:2_18:2) | GP | | PE-O | | 29.989 | | 4.941 | | 12.306 | | 7.638 | | 15.711 | | 7.818 | | Down | |  | |  | |  |
| LIPID-N-0778 | PE(O-18:2_20:1) | GP | | PE-O | | 0.587 | | 0.100 | | 0.245 | | 0.115 | | 0.322 | | 0.102 | | Down | |  | |  | |  |
| LIPID-N-0837 | PE(O-18:2_20:4) | GP | | PE-O | | 162.563 | | 15.953 | | 92.850 | | 44.886 | | 112.039 | | 33.724 | | Down | |  | |  | |  |
| LIPID-N-0790 | PE(O-18:3_18:1) | GP | | PE-O | | 7.087 | | 0.950 | | 3.711 | | 1.992 | | 3.362 | | 1.230 | | Down | |  | |  | |  |
| LIPID-N-0808 | PE(O-18:3_18:2) | GP | | PE-O | | 18.733 | | 4.086 | | 6.478 | | 5.884 | | 7.778 | | 5.685 | | Down | |  | |  | |  |
| LIPID-N-0855 | PE(O-18:3_20:4) | GP | | PE-O | | 55.044 | | 8.558 | | 28.958 | | 17.912 | | 32.888 | | 15.981 | | Down | |  | |  | |  |
| LIPID-N-0869 | PE(O-18:3_20:5) | GP | | PE-O | | 1.239 | | 0.353 | | 0.489 | | 0.480 | | 0.625 | | 0.368 | | Down | |  | |  | |  |
| LIPID-N-0862 | PE(O-18:3_22:4) | GP | | PE-O | | 0.744 | | 0.173 | | 1.369 | | 0.383 | | 1.373 | | 0.531 | | Up | |  | |  | |  |
| LIPID-N-0731 | PE(O-20:0_16:0) | GP | | PE-O | | 0.050 | | 0.012 | | 0.083 | | 0.026 | | 0.093 | | 0.027 | | Up | |  | |  | |  |
| LIPID-N-0797 | PE(O-20:0_20:4) | GP | | PE-O | | 0.281 | | 0.065 | | 0.674 | | 0.358 | | 0.805 | | 0.194 | | Up | |  | |  | |  |
| LIPID-N-0760 | PE(O-20:1_18:1) | GP | | PE-O | | 0.363 | | 0.064 | | 0.271 | | 0.088 | | 0.289 | | 0.064 | | Down | |  | |  | |  |
| LIPID-N-0777 | PE(O-20:1_18:2) | GP | | PE-O | | 0.641 | | 0.102 | | 0.410 | | 0.202 | | 0.452 | | 0.109 | | Down | |  | |  | |  |
| LIPID-N-0873 | PE(O-20:2_22:6) | GP | | PE-O | | 0.234 | | 0.009 | | 0.162 | | 0.041 | | 0.124 | | 0.027 | | Down | |  | |  | |  |
| LIPID-N-0860 | PE(O-20:3_20:4) | GP | | PE-O | | 8.713 | | 1.210 | | 5.515 | | 1.502 | | 6.808 | | 1.608 | | Down | |  | |  | |  |
| LIPID-N-0764 | PE(O-22:0_18:2) | GP | | PE-O | | 0.013 | | 0.003 | | 0.017 | | 0.003 | | 0.024 | | 0.015 | | Up | |  | |  | |  |
| LIPID-N-0782 | PE(O-22:1_18:2) | GP | | PE-O | | 0.107 | | 0.023 | | 0.058 | | 0.028 | | 0.068 | | 0.024 | | Down | |  | |  | |  |
| LIPID-P-0646 | PE(P-15:0_20:3) | GP | | PE-P | | 0.107 | | 0.111 | | 0.365 | | 0.164 | | 0.326 | | 0.165 | | Up | |  | |  | |  |
| LIPID-P-0652 | PE(P-16:0_20:4) | GP | | PE-P | | 1617.882 | | 353.875 | | 1039.418 | | 309.505 | | 1151.797 | | 384.121 | | Down | |  | |  | |  |
| LIPID-P-0638 | PE(P-16:0_22:1) | GP | | PE-P | | 0.274 | | 0.079 | | 0.467 | | 0.198 | | 0.557 | | 0.368 | | Up | |  | |  | |  |
| LIPID-P-1376 | PE(P-16:0_24:5) | GP | | PE-P | | 1.983 | | 0.399 | | 3.973 | | 2.092 | | 3.806 | | 1.512 | | Up | |  | |  | |  |
| LIPID-P-1331 | PE(P-18:0_18:2) | GP | | PE-P | | 49.061 | | 14.103 | | 25.317 | | 9.911 | | 28.837 | | 11.918 | | Down | |  | |  | |  |
| LIPID-P-1323 | PE(P-18:0_20:1) | GP | | PE-P | | 0.865 | | 0.275 | | 0.521 | | 0.256 | | 0.475 | | 0.123 | | Down | |  | |  | |  |
| LIPID-P-1375 | PE(P-18:0_20:5) | GP | | PE-P | | 11.719 | | 4.602 | | 5.348 | | 2.281 | | 6.668 | | 2.823 | | Down | |  | |  | |  |
| LIPID-P-0647 | PE(P-18:1_18:2) | GP | | PE-P | | 75.429 | | 23.656 | | 25.828 | | 18.392 | | 35.592 | | 23.272 | | Down | |  | |  | |  |
| LIPID-P-0658 | PE(P-18:1_20:4) | GP | | PE-P | | 885.051 | | 269.961 | | 427.777 | | 185.880 | | 505.341 | | 183.056 | | Down | |  | |  | |  |
| LIPID-P-1387 | PE(P-18:1_20:5) | GP | | PE-P | | 18.102 | | 4.752 | | 7.614 | | 4.663 | | 11.374 | | 4.874 | | Down | |  | |  | |  |
| LIPID-P-0664 | PE(P-18:1_22:6) | GP | | PE-P | | 130.241 | | 31.825 | | 93.386 | | 23.321 | | 68.193 | | 17.192 | | Down | |  | |  | |  |
| LIPID-P-1339 | PE(P-18:2_16:1) | GP | | PE-P | | 1.737 | | 0.594 | | 0.679 | | 0.358 | | 0.892 | | 0.390 | | Down | |  | |  | |  |
| LIPID-P-1353 | PE(P-18:2_18:2) | GP | | PE-P | | 17.338 | | 4.704 | | 6.113 | | 4.395 | | 7.767 | | 5.139 | | Down | |  | |  | |  |
| LIPID-P-1388 | PE(P-18:2_20:4) | GP | | PE-P | | 301.848 | | 95.233 | | 133.149 | | 90.348 | | 133.805 | | 67.738 | | Down | |  | |  | |  |
| LIPID-P-1396 | PE(P-18:2_20:5) | GP | | PE-P | | 11.492 | | 4.334 | | 4.343 | | 4.206 | | 5.479 | | 3.512 | | Down | |  | |  | |  |
| LIPID-P-1405 | PE(P-18:2_22:6) | GP | | PE-P | | 39.705 | | 13.733 | | 19.522 | | 5.845 | | 13.490 | | 5.434 | | Down | |  | |  | |  |
| LIPID-P-1397 | PE(P-18:3_20:4) | GP | | PE-P | | 11.712 | | 4.545 | | 4.453 | | 3.245 | | 4.905 | | 2.839 | | Down | |  | |  | |  |
| LIPID-P-1400 | PE(P-18:3_22:4) | GP | | PE-P | | 1.303 | | 0.519 | | 0.619 | | 0.213 | | 0.517 | | 0.228 | | Down | |  | |  | |  |
| LIPID-P-1362 | PE(P-19:0_20:4) | GP | | PE-P | | 3.975 | | 1.400 | | 2.367 | | 0.773 | | 2.034 | | 0.669 | | Down | |  | |  | |  |
| LIPID-P-1322 | PE(P-20:0_18:1) | GP | | PE-P | | 0.884 | | 0.264 | | 0.565 | | 0.195 | | 0.570 | | 0.148 | | Down | |  | |  | |  |
| LIPID-P-1333 | PE(P-20:0_18:2) | GP | | PE-P | | 2.583 | | 0.759 | | 1.445 | | 0.842 | | 1.315 | | 0.447 | | Down | |  | |  | |  |
| LIPID-P-0663 | PE(P-20:0_22:6) | GP | | PE-P | | 3.796 | | 1.200 | | 2.388 | | 1.276 | | 1.408 | | 0.618 | | Down | |  | |  | |  |
| LIPID-N-1173 | PG(16:0_18:3) | GP | | PG | | 38.988 | | 4.912 | | 18.574 | | 9.448 | | 21.310 | | 8.627 | | Down | |  | |  | |  |
| LIPID-N-1120 | PG(16:0_22:0) | GP | | PG | | 15.161 | | 1.634 | | 10.654 | | 1.854 | | 12.126 | | 1.993 | | Down | |  | |  | |  |
| LIPID-N-1140 | PG(16:0_22:1) | GP | | PG | | 41.979 | | 5.911 | | 20.019 | | 10.356 | | 25.686 | | 9.807 | | Down | |  | |  | |  |
| LIPID-N-1114 | PG(17:0_18:0) | GP | | PG | | 6.057 | | 0.749 | | 4.526 | | 0.859 | | 3.940 | | 1.147 | | Down | |  | |  | |  |
| LIPID-N-1143 | PG(17:0_22:1) | GP | | PG | | 0.383 | | 0.062 | | 0.194 | | 0.119 | | 0.157 | | 0.081 | | Down | |  | |  | |  |
| LIPID-N-1175 | PG(18:0_16:3) | GP | | PG | | 0.654 | | 0.061 | | 0.326 | | 0.155 | | 0.432 | | 0.147 | | Down | |  | |  | |  |
| LIPID-N-0440 | PG(18:0_18:1) | GP | | PG | | 6.322 | | 0.653 | | 4.473 | | 1.339 | | 4.295 | | 1.056 | | Down | |  | |  | |  |
| LIPID-N-0447 | PG(18:0_18:2) | GP | | PG | | 8.655 | | 1.836 | | 4.285 | | 2.283 | | 4.577 | | 1.393 | | Down | |  | |  | |  |
| LIPID-N-1159 | PG(18:0_19:2) | GP | | PG | | 0.250 | | 0.083 | | 0.149 | | 0.041 | | 0.087 | | 0.038 | | Down | |  | |  | |  |
| LIPID-N-1162 | PG(18:0_20:2) | GP | | PG | | 0.294 | | 0.067 | | 0.219 | | 0.066 | | 0.197 | | 0.059 | | Down | |  | |  | |  |
| LIPID-N-0455 | PG(18:0_20:4) | GP | | PG | | 1.126 | | 0.132 | | 0.853 | | 0.235 | | 0.854 | | 0.224 | | Down | |  | |  | |  |
| LIPID-N-1147 | PG(18:0_22:1) | GP | | PG | | 13.955 | | 2.444 | | 5.706 | | 2.121 | | 6.846 | | 2.411 | | Down | |  | |  | |  |
| LIPID-N-1189 | PG(18:1_18:3) | GP | | PG | | 6.971 | | 1.061 | | 3.452 | | 1.480 | | 3.676 | | 1.176 | | Down | |  | |  | |  |
| LIPID-N-0459 | PG(18:1_20:4) | GP | | PG | | 0.495 | | 0.080 | | 0.689 | | 0.187 | | 0.802 | | 0.278 | | Up | |  | |  | |  |
| LIPID-N-1194 | PG(18:1_22:3) | GP | | PG | | 0.121 | | 0.021 | | 0.082 | | 0.032 | | 0.086 | | 0.028 | | Down | |  | |  | |  |
| LIPID-N-0451 | PG(18:2_18:1) | GP | | PG | | 1.135 | | 0.194 | | 0.719 | | 0.186 | | 0.751 | | 0.251 | | Down | |  | |  | |  |
| LIPID-N-0453 | PG(18:2_18:2) | GP | | PG | | 1.127 | | 0.323 | | 0.509 | | 0.214 | | 0.442 | | 0.136 | | Down | |  | |  | |  |
| LIPID-N-1192 | PG(18:2_21:2) | GP | | PG | | 7.480 | | 0.626 | | 3.743 | | 1.457 | | 4.465 | | 1.726 | | Down | |  | |  | |  |
| LIPID-N-0466 | PG(18:2_22:6) | GP | | PG | | 1.078 | | 0.395 | | 0.690 | | 0.174 | | 0.539 | | 0.076 | | Down | |  | |  | |  |
| LIPID-N-1145 | PG(19:0_20:1) | GP | | PG | | 0.135 | | 0.025 | | 0.097 | | 0.020 | | 0.080 | | 0.034 | | Down | |  | |  | |  |
| LIPID-N-1181 | PG(19:0_20:3) | GP | | PG | | 0.089 | | 0.025 | | 0.191 | | 0.087 | | 0.249 | | 0.162 | | Up | |  | |  | |  |
| LIPID-N-1193 | PG(19:0_20:4) | GP | | PG | | 3.073 | | 0.561 | | 1.560 | | 0.689 | | 1.659 | | 0.610 | | Down | |  | |  | |  |
| LIPID-N-1169 | PG(20:1_22:1) | GP | | PG | | 0.158 | | 0.016 | | 0.092 | | 0.032 | | 0.120 | | 0.038 | | Down | |  | |  | |  |
| LIPID-N-1207 | PG(21:1_20:4) | GP | | PG | | 0.550 | | 0.069 | | 0.228 | | 0.168 | | 0.217 | | 0.103 | | Down | |  | |  | |  |
| LIPID-N-1148 | PG(22:0_18:1) | GP | | PG | | 4.770 | | 0.667 | | 2.852 | | 0.621 | | 3.576 | | 0.608 | | Down | |  | |  | |  |
| LIPID-N-1170 | PG(22:0_20:2) | GP | | PG | | 0.154 | | 0.034 | | 0.089 | | 0.017 | | 0.109 | | 0.027 | | Down | |  | |  | |  |
| LIPID-N-1200 | PG(22:0_20:4) | GP | | PG | | 5.875 | | 0.926 | | 4.428 | | 1.252 | | 4.639 | | 0.755 | | Down | |  | |  | |  |
| LIPID-N-1208 | PG(22:0_20:5) | GP | | PG | | 0.211 | | 0.050 | | 0.132 | | 0.065 | | 0.146 | | 0.055 | | Down | |  | |  | |  |
| LIPID-N-1183 | PG(22:1_18:2) | GP | | PG | | 0.559 | | 0.042 | | 0.379 | | 0.091 | | 0.439 | | 0.135 | | Down | |  | |  | |  |
| LIPID-N-1168 | PG(24:0_18:2) | GP | | PG | | 0.646 | | 0.076 | | 0.313 | | 0.100 | | 0.449 | | 0.138 | | Down | |  | |  | |  |
| LIPID-N-0055 | PGD2 | FA | | Eicosanoid | | 0.050 | | 0.024 | | 0.156 | | 0.106 | | 0.109 | | 0.066 | | Up | |  | |  | |  |
| LIPID-N-1410 | PI(10:0_26:2) | GP | | PI | | 0.798 | | 0.076 | | 0.370 | | 0.218 | | 0.479 | | 0.233 | | Down | |  | |  | |  |
| LIPID-N-1404 | PI(14:1_18:1) | GP | | PI | | 13.500 | | 2.983 | | 5.897 | | 1.732 | | 7.271 | | 2.854 | | Down | |  | |  | |  |
| LIPID-N-1392 | PI(15:0_19:1) | GP | | PI | | 14.540 | | 2.373 | | 8.455 | | 2.415 | | 10.441 | | 3.799 | | Down | |  | |  | |  |
| LIPID-N-1407 | PI(15:0_19:2) | GP | | PI | | 8.131 | | 0.890 | | 3.881 | | 1.872 | | 5.656 | | 2.618 | | Down | |  | |  | |  |
| LIPID-N-0469 | PI(16:0_18:0) | GP | | PI | | 1.546 | | 0.305 | | 0.903 | | 0.331 | | 1.099 | | 0.426 | | Down | |  | |  | |  |
| LIPID-N-0478 | PI(16:0_18:1) | GP | | PI | | 25.645 | | 3.328 | | 13.560 | | 4.956 | | 17.287 | | 5.554 | | Down | |  | |  | |  |
| LIPID-N-1380 | PI(16:0_19:0) | GP | | PI | | 9.891 | | 2.094 | | 7.295 | | 0.720 | | 6.235 | | 1.717 | | Down | |  | |  | |  |
| LIPID-N-0548 | PI(16:0_22:6) | GP | | PI | | 0.527 | | 0.107 | | 0.233 | | 0.044 | | 0.230 | | 0.105 | | Down | |  | |  | |  |
| LIPID-N-1381 | PI(17:0_18:0) | GP | | PI | | 1.877 | | 0.469 | | 1.237 | | 0.373 | | 1.114 | | 0.417 | | Down | |  | |  | |  |
| LIPID-N-0494 | PI(17:0_18:2) | GP | | PI | | 1.173 | | 0.205 | | 0.691 | | 0.358 | | 0.655 | | 0.146 | | Down | |  | |  | |  |
| LIPID-N-0495 | PI(18:0_18:2) | GP | | PI | | 217.745 | | 28.893 | | 95.562 | | 49.206 | | 115.881 | | 51.898 | | Down | |  | |  | |  |
| LIPID-N-0502 | PI(18:0_20:2) | GP | | PI | | 1.464 | | 0.332 | | 0.933 | | 0.175 | | 1.056 | | 0.278 | | Down | |  | |  | |  |
| LIPID-N-1423 | PI(18:0_20:3) | GP | | PI | | 60.171 | | 9.054 | | 38.879 | | 12.698 | | 43.812 | | 12.420 | | Down | |  | |  | |  |
| LIPID-N-0524 | PI(18:0_20:4) | GP | | PI | | 449.947 | | 68.527 | | 301.469 | | 98.081 | | 335.479 | | 92.888 | | Down | |  | |  | |  |
| LIPID-N-0551 | PI(18:0_22:6) | GP | | PI | | 2.361 | | 0.299 | | 1.153 | | 0.300 | | 1.131 | | 0.263 | | Down | |  | |  | |  |
| LIPID-N-0508 | PI(18:1_18:2) | GP | | PI | | 24.754 | | 2.778 | | 9.572 | | 4.889 | | 13.597 | | 7.480 | | Down | |  | |  | |  |
| LIPID-N-0549 | PI(18:1_20:5) | GP | | PI | | 0.450 | | 0.128 | | 0.165 | | 0.094 | | 0.253 | | 0.123 | | Down | |  | |  | |  |
| LIPID-N-0556 | PI(18:1_22:6) | GP | | PI | | 0.758 | | 0.114 | | 0.318 | | 0.121 | | 0.342 | | 0.116 | | Down | |  | |  | |  |
| LIPID-N-0490 | PI(18:2_16:0) | GP | | PI | | 14.653 | | 1.227 | | 7.229 | | 3.247 | | 9.061 | | 4.292 | | Down | |  | |  | |  |
| LIPID-N-1437 | PI(18:2_20:2) | GP | | PI | | 0.576 | | 0.136 | | 0.241 | | 0.127 | | 0.403 | | 0.120 | | Down | |  | |  | |  |
| LIPID-N-1393 | PI(19:0_15:1) | GP | | PI | | 2.170 | | 0.370 | | 1.186 | | 0.397 | | 1.617 | | 0.580 | | Down | |  | |  | |  |
| LIPID-N-1411 | PI(19:0_17:2) | GP | | PI | | 16.444 | | 1.603 | | 7.443 | | 4.467 | | 8.514 | | 4.023 | | Down | |  | |  | |  |
| LIPID-N-0881 | PMeOH(16:0_18:2) | GP | | PMeOH | | 0.044 | | 0.010 | | 0.020 | | 0.016 | | 0.025 | | 0.009 | | Down | |  | |  | |  |
| LIPID-N-1231 | PS(14:0_19:0) | GP | | PS | | 5.865 | | 1.463 | | 10.091 | | 3.637 | | 15.491 | | 10.206 | | Up | |  | |  | |  |
| LIPID-N-1285 | PS(14:1_19:1) | GP | | PS | | 10.359 | | 2.581 | | 4.480 | | 2.619 | | 6.956 | | 2.758 | | Down | |  | |  | |  |
| LIPID-N-1286 | PS(15:0_18:2) | GP | | PS | | 532.081 | | 91.981 | | 265.635 | | 139.379 | | 349.751 | | 128.849 | | Down | |  | |  | |  |
| LIPID-N-0560 | PS(16:0_16:0) | GP | | PS | | 0.277 | | 0.175 | | 1.287 | | 0.672 | | 0.927 | | 0.608 | | Up | |  | |  | |  |
| LIPID-N-1287 | PS(16:0_17:2) | GP | | PS | | 230.276 | | 32.053 | | 120.843 | | 53.263 | | 161.902 | | 50.229 | | Down | |  | |  | |  |
| LIPID-N-1267 | PS(16:0_21:1) | GP | | PS | | 6757.60 | | 1115.43 | | 3621.12 | | 1458.74 | | 4599.85 | | 1527.70 | | Down | |  | |  | |  |
| LIPID-N-1271 | PS(17:0_21:1) | GP | | PS | | 74.450 | | 22.574 | | 36.802 | | 12.288 | | 33.499 | | 13.612 | | Down | |  | |  | |  |
| LIPID-N-1330 | PS(17:1_20:4) | GP | | PS | | 92.057 | | 28.895 | | 50.044 | | 22.294 | | 51.217 | | 15.451 | | Down | |  | |  | |  |
| LIPID-N-0569 | PS(18:0_17:1) | GP | | PS | | 58.786 | | 11.483 | | 41.778 | | 6.844 | | 38.321 | | 11.466 | | Down | |  | |  | |  |
| LIPID-N-0570 | PS(18:0_18:1) | GP | | PS | | 150.888 | | 23.980 | | 263.420 | | 61.444 | | 252.129 | | 71.046 | | Up | |  | |  | |  |
| LIPID-N-1295 | PS(18:0_19:2) | GP | | PS | | 9.994 | | 1.825 | | 6.711 | | 2.727 | | 7.351 | | 1.456 | | Down | |  | |  | |  |
| LIPID-N-0589 | PS(18:0_20:4) | GP | | PS | | 397.139 | | 100.856 | | 213.140 | | 67.515 | | 218.307 | | 67.150 | | Down | |  | |  | |  |
| LIPID-N-1275 | PS(18:0_21:1) | GP | | PS | | 3190.051 | | 811.751 | | 1543.574 | | 469.881 | | 1805.644 | | 556.271 | | Down | |  | |  | |  |
| LIPID-N-1278 | PS(18:0_22:1) | GP | | PS | | 150.227 | | 44.881 | | 65.264 | | 23.756 | | 80.408 | | 29.717 | | Down | |  | |  | |  |
| LIPID-N-0595 | PS(18:0_22:5) | GP | | PS | | 53.904 | | 13.728 | | 92.149 | | 27.510 | | 97.388 | | 31.386 | | Up | |  | |  | |  |
| LIPID-N-0598 | PS(18:0_22:6) | GP | | PS | | 105.443 | | 12.436 | | 211.951 | | 73.813 | | 171.454 | | 74.766 | | Up | |  | |  | |  |
| LIPID-N-1252 | PS(18:0_24:0) | GP | | PS | | 0.994 | | 0.170 | | 1.822 | | 0.513 | | 1.934 | | 0.841 | | Up | |  | |  | |  |
| LIPID-N-1312 | PS(18:1_20:2) | GP | | PS | | 32.166 | | 7.222 | | 17.353 | | 6.679 | | 21.147 | | 5.827 | | Down | |  | |  | |  |
| LIPID-N-1314 | PS(18:1_21:2) | GP | | PS | | 5.720 | | 1.483 | | 2.900 | | 1.458 | | 4.044 | | 1.436 | | Down | |  | |  | |  |
| LIPID-N-1335 | PS(18:1_22:4) | GP | | PS | | 31.407 | | 4.808 | | 19.952 | | 7.199 | | 25.181 | | 4.789 | | Down | |  | |  | |  |
| LIPID-N-0581 | PS(18:2_18:1) | GP | | PS | | 9.004 | | 2.158 | | 5.058 | | 2.547 | | 5.559 | | 2.896 | | Down | |  | |  | |  |
| LIPID-N-1342 | PS(18:2_20:4) | GP | | PS | | 161.692 | | 39.631 | | 57.501 | | 40.395 | | 83.734 | | 54.821 | | Down | |  | |  | |  |
| LIPID-N-1262 | PS(19:0_16:1) | GP | | PS | | 11.841 | | 3.234 | | 22.425 | | 8.862 | | 37.779 | | 23.441 | | Up | |  | |  | |  |
| LIPID-N-0590 | PS(19:0_20:4) | GP | | PS | | 21.206 | | 5.976 | | 12.938 | | 4.960 | | 11.785 | | 1.917 | | Down | |  | |  | |  |
| LIPID-N-1336 | PS(20:1_20:4) | GP | | PS | | 104.032 | | 22.898 | | 45.738 | | 24.151 | | 48.301 | | 17.543 | | Down | |  | |  | |  |
| LIPID-N-1337 | PS(21:0_20:5) | GP | | PS | | 28.727 | | 4.946 | | 15.951 | | 10.131 | | 19.395 | | 7.679 | | Down | |  | |  | |  |
| LIPID-N-1283 | PS(21:0_24:1) | GP | | PS | | 1.726 | | 0.477 | | 2.989 | | 0.746 | | 3.237 | | 1.355 | | Up | |  | |  | |  |
| LIPID-N-1303 | PS(23:0_18:2) | GP | | PS | | 183.226 | | 29.095 | | 103.430 | | 27.639 | | 131.480 | | 30.382 | | Down | |  | |  | |  |
| LIPID-P-0730 | SM(d18:0/16:0) | SP | | SM | | 0.031 | | 0.009 | | 0.102 | | 0.033 | | 0.062 | | 0.023 | | Up | |  | |  | |  |
| LIPID-P-0731 | SM(d18:0/17:0) | SP | | SM | | 0.341 | | 0.100 | | 0.872 | | 0.363 | | 0.779 | | 0.407 | | Up | |  | |  | |  |
| LIPID-P-0732 | SM(d18:0/18:0) | SP | | SM | | 0.947 | | 0.366 | | 2.482 | | 1.111 | | 2.025 | | 1.075 | | Up | |  | |  | |  |
| LIPID-P-0736 | SM(d18:1/14:0) | SP | | SM | | 0.006 | | 0.002 | | 0.010 | | 0.003 | | 0.009 | | 0.002 | | Up | |  | |  | |  |
| LIPID-P-0753 | SM(d18:1/19:1) | SP | | SM | | 0.341 | | 0.089 | | 0.470 | | 0.080 | | 0.519 | | 0.168 | | Up | |  | |  | |  |
| LIPID-P-0765 | SM(d18:2/24:1) | SP | | SM | | 1.145 | | 0.253 | | 1.730 | | 0.228 | | 1.646 | | 0.348 | | Up | |  | |  | |  |
| LIPID-P-1567 | SM(d18:2/24:2) | SP | | SM | | 6.120 | | 1.873 | | 9.043 | | 1.327 | | 8.600 | | 1.595 | | Up | |  | |  | |  |
| LIPID-P-0748 | SM(d20:0/24:1) | SP | | SM | | 2.563 | | 0.962 | | 6.515 | | 1.754 | | 5.700 | | 2.720 | | Up | |  | |  | |  |
| LIPID-P-0094 | SPH(d18:0) | SP | | SPH | | 0.020 | | 0.006 | | 0.035 | | 0.011 | | 0.036 | | 0.014 | | Up | |  | |  | |  |
| LIPID-P-0096 | SPH(d18:1) | SP | | SPH | | 2.320 | | 0.497 | | 1.621 | | 0.180 | | 1.681 | | 0.357 | | Down | |  | |  | |  |
| LIPID-N-0063 | TXB2 | FA | | Eicosanoid | | 0.319 | | 0.097 | | 3.873 | | 2.204 | | 9.804 | | 3.079 | | Up | |  | |  | |  |
|  |  |  | |  | |  | |  | |  | |  | |  | |  | |  | |  | |  | |  |

Units are expressed as nmol/g

**Supplemental Data Figure S1: Quality control (QC) metrics.**

**A.**

**(A)** Pearson correlation analysis was performed on QC samples. The closer the | r | to 1, the higher the correlation between two samples. Diagonal squares represent individual QC samples; Left diagonal box represents scatter diagram of QC samples. Both x-axis and y-axis represent lipid content. Each dot in the diagram represents an individual lipid. The right diagonal box represents correlation coefficients of the QC samples. All QC samples exhibited an r^2^ >0.995.

**B.**

**(B)** Coefficient of variation (CV) plot for QC and sample groups. Horizontal axis represents CV value and vertical axis represents the percent of peaks (proportion of metabolites). The Coefficient of Variation (CV) value is the ratio between the standard deviation of the original data and the mean of the original data, which can reflect the degree of data dispersion. The Empirical Cumulative Distribution Function (ECDF) was used to analyze the frequency of compound CVs that is smaller than the reference value. The higher the proportion of compounds with low CV value in the QC samples, the more stable the experimental data. As a rule of thumb, the proportion of compounds with CV value less than 0.5 in the QC samples is higher than 85% indicates that the experimental data is relatively stable. The proportion of compounds with CV value less than 0.3 in the QC samples is higher than 75% indicates that the experimental data is very stable.

**C.**

**(C)** Principal component 1 (PC1) variation of all samples. Horizontal axis represents injection order of samples and vertical axis represents standard deviation (SD) of PC1 score.

**Supplemental Data Figure S2: OPLS-DA model validation.**

B.**1ppm PFOA vs CTL**
C. **5ppm PFOA vs CTL**

The OPLS-DA model was used to analyze metabolome data and draw score charts for each group to further show the differences between each group (Thevenot et al., 2015). The prediction parameters of the evaluation model are R^2^X, R^2^Y and Q^2^, where R^2^X and R^2^Y represent the explanatory rate of the model to X and Y matrix respectively, and Q^2^ represents the prediction ability of the model. The closer these three indicators are to 1, the more stable and reliable the model is. Q^2^> 0.5 can be considered as effective model, and Q^2^> 0.9 can be considered an excellent model. The horizontal coY-axis represents the model accuracy, and the vertical coY-axis is the frequency of the model classification effect. The model performs bootstrapping 200 times and if Q^2^’s P = 0.02, it indicates that the prediction ability of four random grouping models is better than that of the OPLS-DA model in the Permutation detection. If R^2^Y’s P = 0.545, it indicated that there were 109 random grouping models in the Permutation detection, whose explanation rate of Y matrix was better than that of the OPLS-DA model. In general, p < 0.05 is the best model.
